# Mapping Genetic Variants Associated with *Beta*-Adrenergic Responses in Inbred Mice

**DOI:** 10.1371/journal.pone.0041032

**Published:** 2012-07-31

**Authors:** Micha Hersch, Bastian Peter, Hyun Min Kang, Fanny Schüpfer, Hugues Abriel, Thierry Pedrazzini, Eleazar Eskin, Jacques S. Beckmann, Sven Bergmann, Fabienne Maurer

**Affiliations:** 1 Department of Medical Genetics, University of Lausanne, Lausanne, Switzerland; 2 Swiss Institute of Bioinformatics, Lausanne, Switzerland; 3 Department of Computer Science and Department of Human Genetics, University of California Los Angeles, Los Angeles, California, United States of America; 4 Department of Biostatistics, Center for Statistical Genetics, University of Michigan, Ann Arbor, Michigan, United States of America; 5 Service of Medical Genetics, Centre Hospitalier Universitaire Vaudois and University of Lausanne, Lausanne, Switzerland; 6 Department of Clinical Research, University of Bern, Bern, Switzerland; 7 Department of Medicine, Centre Hospitalier Universitaire Vaudois and University of Lausanne, Lausanne, Switzerland; University of Minnesota Medical School, United States of America

## Abstract

*β*-blockers and *β*-agonists are primarily used to treat cardiovascular diseases. Inter-individual variability in response to both drug classes is well recognized, yet the identity and relative contribution of the genetic players involved are poorly understood. This work is the first genome-wide association study (GWAS) addressing the values and susceptibility of cardiovascular-related traits to a selective *β*_1_-blocker, Atenolol (*ate*), and a *β*-agonist, Isoproterenol (*iso*). The phenotypic dataset consisted of 27 highly heritable traits, each measured across 22 inbred mouse strains and four pharmacological conditions. The genotypic panel comprised 79922 informative SNPs of the mouse HapMap resource. Associations were mapped by Efficient Mixed Model Association (EMMA), a method that corrects for the population structure and genetic relatedness of the various strains. A total of 205 separate genome-wide scans were analyzed. The most significant hits include three candidate loci related to cardiac and body weight, three loci for electrocardiographic (ECG) values, two loci for the susceptibility of atrial weight index to *iso*, four loci for the susceptibility of systolic blood pressure (SBP) to perturbations of the *β*-adrenergic system, and one locus for the responsiveness of QTc (*p*<10^−8^). An additional 60 loci were suggestive for one or the other of the 27 traits, while 46 others were suggestive for one or the other drug effects (*p*<10^−6^). Most hits tagged unexpected regions, yet at least two loci for the susceptibility of SBP to *β*-adrenergic drugs pointed at members of the hypothalamic-pituitary-thyroid axis. Loci for cardiac-related traits were preferentially enriched in genes expressed in the heart, while 23% of the testable loci were replicated with datasets of the Mouse Phenome Database (MPD). Altogether these data and validation tests indicate that the mapped loci are relevant to the traits and responses studied.

## Introduction

The *β*-adrenergic system controls cardiac contractility and excitability, heart rate and vascular tone. Atenolol (*ate*), a selective *β*_1_ receptor antagonist, belongs to the preferred *β*-blocking therapies for the management of heart failure, myocardial infarction, angina and atrial fibrillation, and to the acceptable first-line therapies for hypertension [1], [2]. In contrast, Isoproterenol (*iso*) is a *β*-agonist that may be administered in cases of bradycardia, heart block and asthma [3], [4]. In experimental settings, long-term administration of *iso* to rodents and other mammals is also used to model left ventricular hypertrophy (LVH), independently of blood pressure [5]. This is of important clinical relevance because unless being a consequence of chronic exercise, LVH is considered as a pathological manifestation in man. When persisting, this insult may ultimately lead to heart failure.

Inter-individual variability in response to either drug is well recognized. In humans, associated clinical concerns pertain to variance in drug efficacy and occasional adverse side-effects [6], [7], [8]. In inbred mice, extensive inter-strain variability of cardiovascular-related trait values and responsiveness to either drug was demonstrated earlier [9]. For these latter phenotypes, heritabilities were typically higher than 0.5 (Table S2), indicating that a large fraction of the phenotypic variance was under genetic control.

Until recently, the search for pharmacogenetic determinants primarily addressed the effects of single genes and variants on drug disposition and drug response through candidate gene studies. For *ate* and *iso*, the focus was put on genes coding for *β*-adrenergic receptors [10], [11], [12], [13] and members of the downstream signaling pathways [6], [14], [15], [16], [17]. While convincing evidence indicated that variation within some of these candidates had an impact on therapy, it was acknowledged that the underlying genetic architecture involved additional, yet to be found determinants [6].

A more detailed understanding of these determinants and associated pathways may ultimately lead to new strategies for better tailored treatment in patients. Our aim was therefore to provide the scientific community with a set of complementary biological hypotheses regarding the genetic determinants of the cardiovascular response to *ate* and *iso*. By generating candidate loci related to a given phenotype in an unbiased manner, genome-wide association studies (GWAS) offer a powerful tool to address this goal.

So far, a small number of GWAS have successfully addressed drug responses or (adverse) effects in humans [18] but none of them was centered on *β*-blockers or *β*-agonists [19]. Specific difficulties, pertaining to sample size, replication of findings, phenotype heterogeneity and effect size, make that these investigations are particularly challenging to implement in humans [18]. Moreover, controlling for environmental confounders such as compliance to drug prescription, poly-medication, placebo effect or diet, to name a few, can be difficult.

To overcome these limitations, our experiments were performed in a genetic reference population (GRP) of 22 inbred mouse strains. The benefit of using such a GRP is that because it is genetically stable and reproducible, it allows data integration across time and (independent) studies and creates the possibility to uncover relationships among genes, pathways, and diseases in well controlled conditions. The phenotypic dataset consisted of the 27 cardiovascular-related traits measured earlier across the various strains and conditions of treatment [9]. To screen for candidate loci, we used efficient mixed model association (EMMA) mapping, a method that corrects for the complex population structure and genetic relatedness of the inbred strains [20], and applied conservative adjustment for multiple hypotheses testing.

In a first series of 105 analyses, we searched for associations between the non-transformed values of the 27 traits and ca 80000 informative SNPs of the mouse HapMap resource [21]. In the second part of the study, we probed 100 genome-wide scans for markers associated with pharmacological effects, using approximated values of trait responsiveness. To substantiate the mapped loci further, we investigated potential overlaps with independent functional or genetic evidence obtained in the literature and various databases of linkage studies, animal mutants and human GWAS [19], [22], [23]. We then show that the loci mapped for cardiac traits are enriched for genes expressed in the heart, while a significant fraction (23%) of the testable candidates are replicated in independent cohorts of the MPD [24].

## Results

### 1. GWAS of 27 trait values measured across 22 mouse strains and four drug conditions

The dataset of 27 phenotypes measured across 22 inbred mouse strains and four drug conditions was described earlier (Table S1 and [9]). On average, eight to ten biological replicates were characterized in each strain and treatment condition. To probe for associations between these values and the informative SNPs of the mouse HapMap [21], we generated a separate genome-wide analysis for each trait and treatment condition (*ctr*, *ate*, *iso1* or *iso10* [9]). With the exception of normalized cardiac indices (AWI, VWI), phenotypes were not adjusted or transformed prior to searching for associations. In total, 105 EMMA scans were produced (Figures S1, S2, S3 and S4). Quantile-quantile plots (QQ-plots) were used to monitor the quality of the GWAS. Hits were declared nominally significant for *p*-values ≤10^−8^ and suggestive for *p*-values ranging from 10^−6^ to 10^−8^ (Material and Methods, Figure S11).

Based on these filters, 50 genome-wide scans produced at least one suggestive association, 39 had no hit and 16 were excluded because they demonstrated an inflation of *p*-values of low to moderate significance (Figures S1, S2, S3 and S4, Table S3). Careful examination of the allelic patterns underlying the significant SNPs, followed by manual imputation of those with missing alleles, indicated that nine loci were likely candidates, two of which shared identical strain distribution patterns (SDPs) on separate chromosomes (Table 1). In addition, 60 other loci were suggestive for one or the other of the 27 traits (Table S3).

**Table 1:** Top hits for the set of 27 cardiovascular-related traits.

Loc	Chr	Pos	Trait	Treatmt	Majallele	Minallele	SDP	Top *p*-val	Corr. *p*-val	Effect size	Corr. Effect size	Tagged genes	Summary
1	5	89981612–91491392	AW	*ate*	12	10	full	**1.27×10** ^−**16**^		2.06 mg		*Npffr2, Adamts3, Ankrd17, Afp, Afm*	candidate
2	5	97755919–98799267	AW	*ctr*	13	9	full	**8.16×10** ^−**10**^		1.96 mg		*1700007G1Rik*	candidate
3	15	96603063–96941901	AW/BWS	*iso 1*	12	8	incomplete	9.79×10^−9^		0.057 mg/g		*intergenic*	candidate
4*	6	78327461–78433200	VW/AW	*iso 10*	15	5	incomplete	4.66×10^−10^	8.87×10^−11^	3.91	3.85	*Reg3d*	candidate
5	7	129765856	VW/AW	*ctr*	16	6	full	**4.65×10** ^−**9**^		3.77		*Prkcb*	candidate
6*	9	115307951–115706566	VW/AW	*iso 10*	17	5	full	**8.87×10^−11^**		3.85		*intergenic*	candidate
7^#^	2	18754798–26103095	BWE/BWS	*iso 10*	17	4	incomplete	3.77×10^−10^	2.39×10^−8^	0.125	0.121	*many*	suggestive
9	6	143980402–144448909	QRS	*ctr*	14	8	full	**3.76×10** ^−**9**^		2.06 ms		*Sox5*	candidate
10	4	123753350	QTc	*iso 10*	14	8	full	**2.24×10** ^−**9**^		9.46 ms		*intergenic*	candidate
11	14	83530089	QTc	*ate*	14	8	full	**4.28×10** ^−**10**^		5.71 ms		*intergenic*	candidate
17	1	40082962	SBP	*iso 1*	16	5	incomplete	7.11×10^−8^		19.36 mmHg		*intergenic (Map4k4, Il1r2)*	suggestive
-	17	33222672	HR (TC)	*ate*	12	10	full	**5.4×10** ^−**9**^		67.85 bpm		*intergenic (Zfp563)*	candidate ?

For each locus, the *p*-value of the most significant hit is given as calculated in EMMA. Scores are highlighted in bold letters when they were obtained at SNPs with fully documented allelic information. When this information was incomplete, the corrected score was imputed manually (Material and Methods). Corr. Effect size: effect size at the surrogate SNP used for imputation; *: loci 4 and 6 share identical SDPs on separate chromosomes; ^#^: the SDP of locus 7 is compatible with that of suggestive locus 32 for the same trait (Table S3); bpm: beats per min; nf: not found.

**Figure 1:**
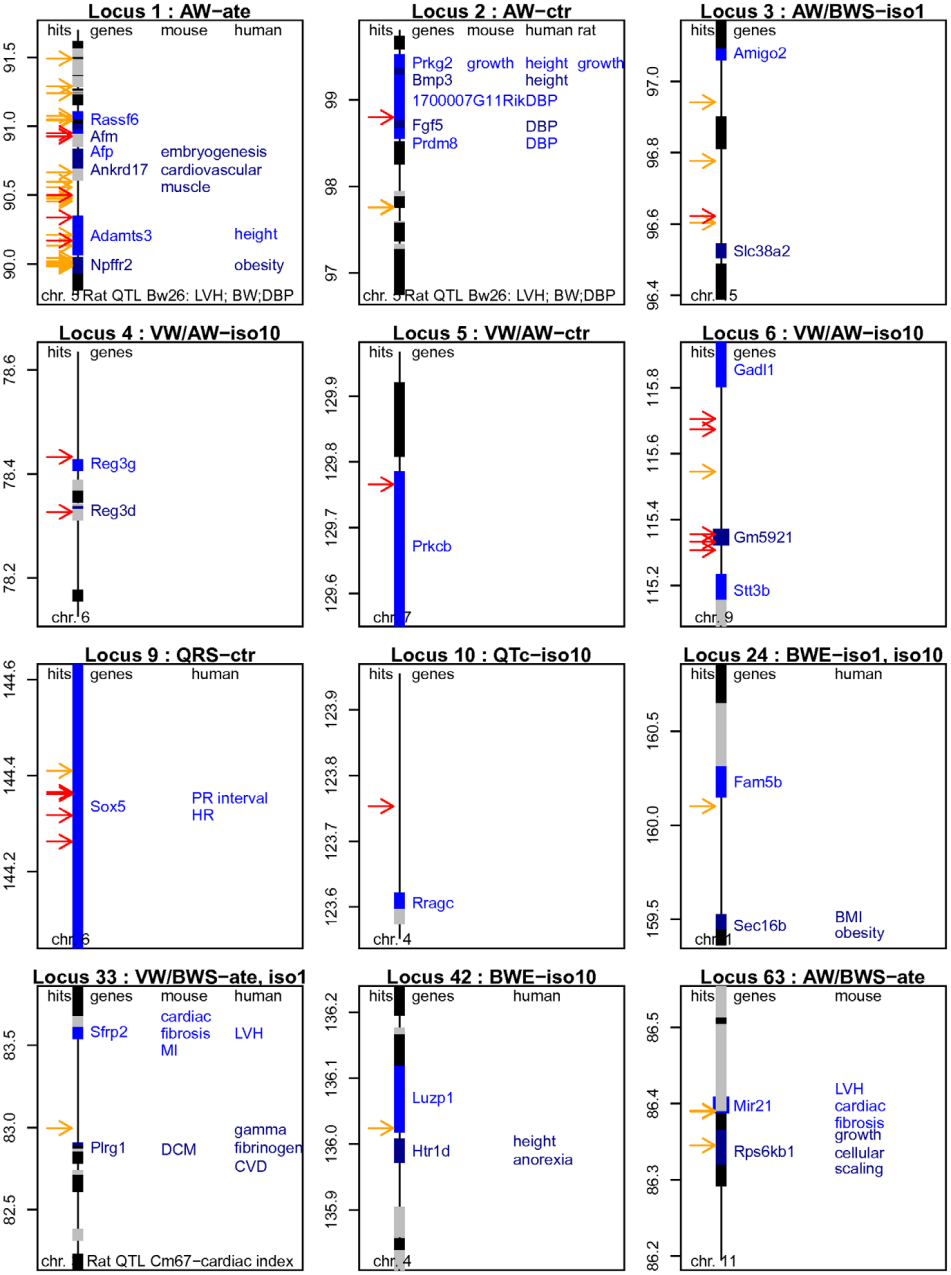
Synoptic maps of the top loci for cardiovascular-related traits. The genetic maps of twelve loci are presented. Chromosomal positions are given on the vertical axis (in Mb). Colored boxes are used to differentiate contiguous genes. The “trait plus treatment” combinations producing the strongest associations are indicated in each plot’s title. The positions of the significant and suggestive hits are indicated by red and orange arrows, respectively (all hits are detailed in Table S3). Potential overlaps linking these loci with independent evidence obtained from functional or genetic studies performed in mice, humans and rats are annotated in the respective vertical tracks (see main text for details). For instance, variants of *ADAMTS3* are associated with human height (locus 1). Overlaps with rat QTLs are indicated at the bottom of the maps for loci 1, 2 and 33. DBP: diastolic blood pressure; MI: myocardial infarction; DCM: dilated cardiomyopathy; LVH: left ventricular hypertrophy; CVD: cardiovascular disease.

In the paragraphs below, we provide detailed descriptions of the mapping results for the traits related to body weight (BWS, BWE), cardiac weight (HW, VW and AW), cardiac indices (VWI and AWI), systolic blood pressure (SBP), heart rate (HR) and ECG values. Readers less interested in these details are referred to Figure 1 for a synoptic view and to Discussion for a general perspective of these results.

### Non-adjusted weight of the heart and cardiac compartments (HW, VW, AW)

The morphological phenotypes were robust and highly correlated [9]. Of all the EMMA scans obtained with these traits, those for non-adjusted weight of the cardiac atria (AW) produced the strongest hits (−log_10_ *p* = 15.9; Table 1). While no hit passed the threshold of suggestive significance for non-adjusted HW and VW (Figures S1, S2, S3 and S4), a small number of additional candidates were mapped for body weight and cardiac indices.

**Figure 2:**
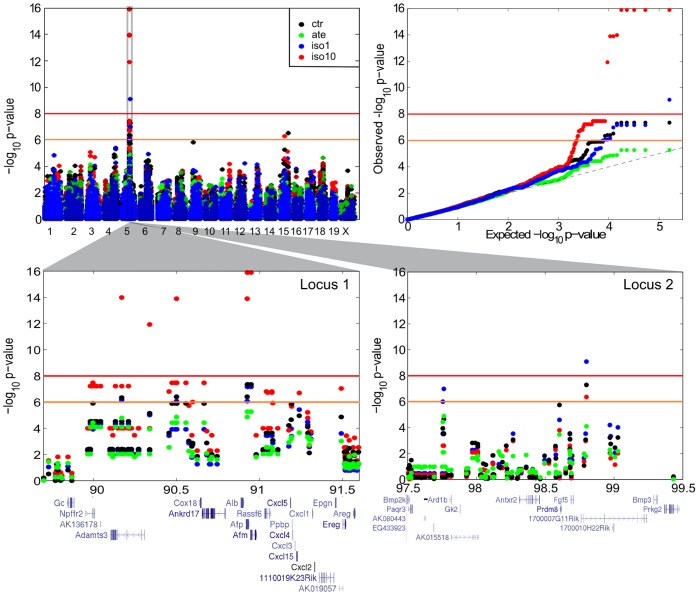
GWAS of non-adjusted AW. A. Composite Manhattan plot superimposing the four EMMA scans for non-adjusted AW. Association scores (-log_10_ *p*-value; y-axis) are shown for each chromosomal position (x-axis) tested by EMMA. Suggestive and significant thresholds of association are indicated by orange and red lines, respectively. B. QQ-plots reporting the -log_10_ of *p*-values as obtained by EMMA. The null hypothesis (*H_0_*) is indicated by the dotted line. A strong upper deviation from this line in the region of the lowest *p*-values is indicative of a true association. C, D. Zoom-in on loci 1 and 2. Genes are displayed as indicated in the NCBI37/mm9 assembly of the UCSC genome browser [86].

In the panel A of Figure 2, the four Manhattan plots for AW - one per treatment condition - are superimposed. Highly significant hits mapped onto two ca 8 Mb-apart regions of chr 5, called loci 1 and 2 (Table 1, Figure 2). The number of SNPs with low genome-wide *p*-values was well above that expected by chance, in particular for values measured in *ctr* and *ate*-treated mice (Figure 2B).

Locus 1 is a 1.5 Mb segment spanning 18 genes. It is delineated by nine highly significant and 24 suggestive markers preferentially associated with AW of *ate*-treated mice (Table 1). The top positions were replicated in the *ctr* and *iso1*-treated cohorts, albeit at slightly reduced significance (Figures S1, S2, S3 and S4, Table S3).

Locus 2 encompasses a 1 Mb region tagged by one significant and one suggestive SNPs associated with AW of *ctr* mice. Similarly to locus 1, the top hit was suggestively replicated with AW of *ate*- and *iso1*-treated animals.

Both loci were also significant when considering AW of all mice, using treatment as a covariate in EMMA (data not shown). They further overlapped with some of the very few suggestive SNPs retrieved with body weight (BWS for both loci and BWE for locus 2; Figures S1, S2, S3 and S4, Table S3). In contrast, their association scores with the other phenotypes, in particular normalized cardiac indices (AWI and VWI or HWI), were low.

Both intervals include several genes, of which *Npffr2* (neuropeptide FF receptor 2), *Adamts3* (a metalloproteinase of the ECM), *Alb* (serum albumin), *Afp* (á-feto-protein), *Afm* (afamin), and *1700007G11Rik* carry the most significant hits (Figure 2). Our microarrays indicated that *Cox18* and *Ankrd17* were expressed in mouse cardiac ventricles, while the other transcripts were either low (*Adamts3*) or absent (Table S5). Functional studies in animal models [22] pointed at possible roles in cardiovascular- and/or growth-related phenotypes for *Ankrd17* (locus 1) and *Prkg2* (which resides ca 0.56 Mb downstream of locus 2). Specifically, *Ankrd17*-deficiency led to serious hemorrhages and embryonic lethality in the mouse [25]. Secondary perturbations included arrest of the endocardium development, thinner myocardium and poor trabeculation in the cardiac ventricle. As of *Prkg2*, several mutant forms cause mammalian dwarfism [26], [27]. Similar functional approaches seem to exclude *Alb, Afp*, *Fgf5* and *Bmp3* from the list of putative candidates, while mutants of the remaining genes have not been described [22].

Our results seem to intersect with data obtained by others (Figure 1). First, the syntenic segments of loci 1 and 2 on rat chr 14 are encompassed by *Bw26*, a 28 Mb QTL that peaks in the direct vicinity of *Alb* (Figure 2) and is associated with hypertrophy of the left cardiac ventricle, body weight, and systemic arterial diastolic blood pressure [23], [28]. Second, human variants of *ADAMTS3* (locus 1) and *PRKG2/BMP3* (i.e. immediately downstream of locus 2) are associated with adult height [29], [30], [31], a particular haplotype of *NPFFR2* (locus 1) confers protection against obesity in Scandinavians [32] and the syntenic human segment overlapping locus 2 is associated with diastolic blood pressure [33]. The low scores for cardiac indices and SBP in our analyses suggest that loci 1 and 2 might be preferentially involved in the control of organ size and/or weight and/or scaling.

### Adjusted weight of the cardiac compartments (VWI, VW/BWS, AWI, AW/BWS)

We mapped two, respectively seven suggestive loci for ventricular (VWI and VW/BWS) and atrial (AWI and AW/BWS) weight indices. These regions did not overlap. As left ventricular hypertrophy, such as that induced by *iso*, is a major risk factor for ventricular dysfunction and heart failure [5], we paid particular attention to all SNPs associated with cardiac indices.

The most significant region for ventricular indices was tagged by one intergenic SNP of chr 3 (locus 33, *rs*31137966; Figure 1 and Figures S1, S2, S3 and S4, Table S3). This marker mapped on a 0.75 Mb segment located between *Plrg1* and *Sfrp2*. Plrg1 is a ubiquitous and evolutionarily conserved component of the spliceosome, while Sfrp2 is an enhancer of procollagen C proteinase. Both genes may be relevant candidates. First, they are both expressed in cardiac tissues (Table S5). Second, in the rat, they belong to *Cm67* (*CakmC2QTL*), a 25 Mb QTL associated with cardiac index [34]. Third, the human orthologues belong to segments mapped for left ventricular mass index in a linkage study of human siblings and for ã-fibrinogen, a marker of cardiovascular disease, in a GWAS [35], [36]. Fourth, in rodents, Sfrp2 is involved in fibrosis associated with myocardial infarction [37], [38]. And fifth, in mice, cardio-specific inactivation of *Plrg1* induces massive dilatation and atrophy of both cardiac ventricles characteristic of severe dilated cardiomyopathy, further leading to post-natal lethality [39].

Locus 43 was apparently also suggestively associated with the ventricular indices of *iso*-treated mice (locus 43, *rs*6339927 and *rs*32349124; Figures S1, S2, S3 and S4, Table S3). Yet, manual imputation of the underlying markers, followed by score correction, suggested that this locus was a false positive. Specifically, -log_10_ *p_iso1_* at *rs*32838436 was 3.89, while -log_10_ *p_iso10_* at *rs*33032029 was 2.78.

The strongest signals for atrial indices hit locus 3, a 0.35 Mb segment of chr 15 defined by four variants of interest (Figure 1). The top score was retrieved with AW/BWS of *iso1*-treated mice (Table 1), while three nearby SNPs were suggestively replicated across several scans for AWI, AW/BWS and non-adjusted AW (Figures S1, S2, S3 and S4, Table S3). All four SNPs lacked genotypic information in one or two of the 22 strains (Table 1). In the absence of surrogate variants with full allelic information, the corrected scores could not be imputed. Locus 3 is located in the proximity of two genes coding for ion transporters, *Slc38a2* and *Slc38a4*. The product of *Slc38a2* is a ubiquitous sodium-dependent neutral amino acid transporter, called SNAT2 in humans. Given its high expression level in cardiac tissues (Table S5) and its role in the regulation of cell volume [40], *Slc38a2* might be relevant to the morphology of cardiac atria.

At least three of the six additional suggestive loci for atrial indices were of potential interest: loci 20, 59, 63. Locus 20 was defined by a single variant tagging a 1.8 Mb gene desert between *Slc4a3* and *Epha4* on chr 1 (locus 20, *rs*31792085; Figures S1, S2, S3 and S4, Table S3). Interestingly, a variant of the syntenic human segment on chr 2q36.1 is suggestively associated with left ventricular mass [41]. Moreover, *Slc4a3* codes for a Cl^−^/HCO_3_^−^ anion exchanger that is highly expressed in the heart (Table S5). While disruption of *Slc4a3* alone has no adverse effects on cardiac performance, functional investigations in mouse mutants suggest that it is involved in cardiac contractility, *β*-adrenergic responses and cardiac Ca^2+^ handling [42], [43].

Locus 59, a 0.16 Mb region defined by three SNPs with identical SDPs, was associated with atrial indices of *ate*-treated individuals (Figures S1, S2, S3 and S4, Table S3). Scores in the other scans were much reduced, suggesting that the underlying variants were preferentially associated with *β*-blockade. These markers tagged *Tbk1* (TANK-binding kinase 1, a signaling molecule of immune responses and inflammation), *Xpot* (exportin t, a t-RNA nuclear exporting factor), and *Rassf3* (a member of Ras effectors and tumor suppressor genes). Microarrays indicated that both *Xpot* and *Rassf3* were expressed in cardiac tissues (Table S5) but their potential functions in the heart are not known.

Locus 63, a 45 kb interval defined by four suggestive SNPs sharing identical SDPs, was associated with the AW/BWS ratio of *ate*-treated mice (Figure 1 and Figures S1, S2, S3 and S4, Table S3). Scores were only slightly reduced in the *ctr* and *iso1*-treated cohorts. Interestingly, the syntenic rat segment on chr 10 is encompassed by *Cm51*, a 43 Mb QTL associated with cardiac mass and hypertension that peaks in close vicinity of these markers [44]. Tagged genes include *Rps6kb1* (ribosomal S6 kinase), *Tubd1* (tubulin delta 1), *Tmem49* (a trans-membrane protein) and *miR21*. All of them are expressed in cardiac tissues and thus might be relevant for the physiology of cardiac atria. S6 kinase in particular is a pivotal member of the mTOR/p70(S6K) signaling cascade that is involved in the regulation of cell size and scaling. Homozygous disruption of mouse *Rps6kb1* does not affect viability or fertility, but induces a significant decrease of body and organ growth, especially during embryogenesis [45], [46]. Equally interesting, *miR-21* is up-regulated in several models mimicking cardiac hypertrophy, such as neonatal cardiomyocytes treated with *iso* [47] or mice subjected to trans-aortic banding [48], [49], [50]. Functional investigations further suggest that *miR-21* contributes to myocardial disease by activating molecular mechanisms of cardiac fibrosis [51], [52].

### Body weight (BWS and BWE)

Five loci were suggestively associated with body weight, of which at least four tagged seemingly relevant candidate regions and/or overlapped hits mapped for related traits. In addition to the two suggestive regions mentioned above (see chapter on scans for non-adjusted AW), an intergenic SNP of chr 1 located ca 0.6 Mb downstream of *Sec16b* was suggestively associated with BWE of *iso*-treated animals (locus 24, *rs*32046817; Figure 1 and Figures S1, S2, S3 and S4, Table S3). This region appeared just below threshold in the remaining analyses (*ctr*, *ate*) and was suggestive when considering BWE of all mice, using treatment as a covariate (data not shown). Interestingly, human variants of and around *SEC16B* have been repeatedly associated with body mass index and obesity [53], [54].

Another intergenic marker, located between *Luzp1* and *Htr1d* on chr 4, was suggestively associated with BWE of *iso10*-treated mice (locus 42, *rs*32851583; Figure 1 and Figures S1, S2, S3 and S4, Table S3). Genome-wide significance was only slightly reduced in the remaining analyses (*ctr*, *ate*, *iso1*). Polymorphisms around human *HTR1D* (serotonin receptor 1D) are associated with adult height [30] and, suggestively, appetite control and anorexia [55], which may all be relevant to BW.

### Body weight gain (BWE/BWS)

Significant associations for body weight gain were specifically identified using trait values of *iso10*-treated mice, mapping on a ca 7.5 Mb segment of chr 2 (locus7, Table 1). Closer inspection of the underlying SDPs indicated that the most significant scores were slightly inflated. Moreover, these genotypes shared SDPs compatible with those of suggestive locus 32 on chr 3 (Table S3), which complicates data interpretation. The mechanisms behind differential body weight gain upon *iso10* stimulation are not known. Locus 7 contains more than 100 genes, while locus 32 best tags *Fstl5* (follistatin-like 5) and *Golim4* (a membrane protein of the Golgi).

### Systolic blood pressure (SBP)

The identification of genetic variants affecting human blood pressure and hypertension has been particularly challenging [33], [56], [57] and our results are in line with this trend. Thus, a single signal passed the suggestive filter for SBP (locus 17, Table 1). This hit consisted in an isolated intergenic SNP on chr 1, with associations at nearby markers well below thresholds. Evidence supporting a possible link between this region and blood pressure, whether from human GWAS, mutant mice, other animal models or functional studies, is currently lacking. Yet, as summarized in Table 2 and discussed below, this hit was replicated with the independent values of the Sugiyama1 cohort of the MPD [24], [58].

**Table 2:** Top hits for the set of approximated drug effects.

Loc	Chr	Pos	Trait	Effect	Maj allele	Min allele	SDP	Top*p*-val	Corr. *p*-val	Effectsize	Corr.Effectsize	Taggedgenes	Summary
AE1	1	34187903	AWI	*iso10 vs ctr*	12	8	incomplete	**3.33×10** ^−**12**^	1.76×10^−6^	0.14 mg/g	0.12 mg/g	*Dst*	inflated
AE2	3	109575377–111099257	AWI	*iso10 vs ctr*	12	10	full	**4.36×10** ^−**9**^		0.13 mg/g		*Ntng1*	candidate
AE3	5	63633898–64902978	QTc	*iso10 vs ate*	13	9	full	**9.96×10** ^−**9**^		0.18 ms		*intergenic*	candidate
AE4	6	53371578–54166499	SBP	*iso10 vs ate*	16	6	full	**5.59×10** ^−**12**^		0.11 mmHg		*Creb5, Chn2*	candidate
AE4	6	53371578–54166499	SBP	*ate vs iso10*	16	6	full	**1.23×10** ^−**9**^		0.1 mmHg		*Creb5, Chn2*	candidate
AE6*	10	113842415–114024166	SBP	*iso10 vs ate*	15	7	full	**2.88×10** ^−**12**^		0.11 mmHg		*Trhde*	candidate
AE6*	10	113842415–114024166	SBP	*ate vs iso10*	15	7	full	**8.48×10^−11^**		0.1 mmHg		*Trhde*	candidate
AE7	11	107858635–108750692	SBP	*iso10 vs ate*	14	7	incomplete	8.95×10**^−^** ^11^	4.36×10**^−^** ^6^	0.1 mmHg	0.09 mmHg	*Prkca*	inflated
AE7	11	107858635–108750692	SBP	*ate vs iso10*	14	7	incomplete	4.33×10^−9^	9.07×10**^−^** ^5^	0.09 mmHg	0.08 mmHg	*Prkca*	inflated
AE8	11	112024896–112221449	AWI	*iso10 vs ctr*	13	9	full	**4.78×10^−9^**		0.13 mg/g		*intergenic (Kcnj2, Sox9)*	candidate
AE9	14	22803665–23286950	SBP	*iso10 vs ate*	15	5	incomplete	3.04×10^−10^	9.65×10^−5^	0.1 mmHg	0.09 mmHg	*1700112E06Rik*	inflated
AE9	14	22803665–23286950	SBP	*ate vs iso10*	15	5	incomplete	8.37×10^−9^	6.7×10^−6^	0.1 mmHg	0.09 mmHg	*1700112E06Rik*	inflated
AE10	15	43683377–44902768	SBP	*iso10 vs ate*	14	8	full	**6.58×10** ^−**12**^		0.1 mmHg		*Trhr, Tmem74*	candidate
AE10	15	43683377–44902768	SBP	*ate vs iso10*	14	8	full	**4.75×10^−10^**		0.09 mmHg		*Trhr, Tmem74*	candidate
AE11*	18	68266538–68314407	SBP	*iso10 vs ate*	15	7	full	**2.88×10^−12^**		0.11 mmHg		*D18Ertd653e*	candidate
AE11*	18	68266538–68314407	SBP	*ate vs iso10*	15	7	full	**8.48×10^−11^**		0.1 mmHg		*D18Ertd653e*	candidate

See Table 1 for the details of the abbreviations.

### Heart rate in conscious mice (HR)

Inspection of the Manhattan and QQ-plots for HR (TC) indicated that either no hit passed the suggestive threshold (*ctr* and *iso1*) or the EMMA scans were inflated with *p*-values of low to moderate significance (*ate* and *iso10*; Figures S1, S2, S3 and S4). A candidate region that might survive correction for inflation was defined by a single significant SNP located immediately upstream of *Zfp563* on chr 17 (chr 17, *rs*33155499; Table 1). This hit was the strongest with the values of *ate*-treated mice but markedly less significant in *ctr* condition or under *β*-adrenergic stimulation. *Zfp563* encodes a transcription factor of unknown function. Yet, in the rat, *Zfp563* is encompassed within *Hrtrt10*, a 27 Mb QTL associated with HR of females placed under low-salt diet and volume depletion with furosemide [59].

### ECG traits

A small number of candidate loci were obtained with ECG trait values, in particular QRS interval and QTc (Table 1). Significant associations were also mapped for Qamp and Ramp (Figures S1, S2, S3 and S4, Table S3). Yet, since amplitudes and areas of ECG waves are composite parameters of little clinical use, these data are not discussed further. For the other traits, the scores did not go beyond suggestive significance (Figures S1, S2, S3 and S4, Table S3).

Four significant and two suggestive hits of chr 6 were specifically associated with QRS interval of *ctr* mice (locus 9, Figure 1 and Table 1). The underlying 0.5 Mb candidate segment tagged *Sox5*, a transcription factor involved in embryonic development and chondrogenesis [60]. Functional evidence linking *Sox5* with cardiac electrical activity is lacking but variation in human *SOX5* is associated with PR interval [61] and resting HR [62]. Our results might be in line with these data, even though the scores for PR or HR were low in this region (Figures S1, S2, S3 and S4, Table S3).

Two isolated intergenic SNPs were significantly associated with QTc (loci 10 and 11, Table 1). The strongest one, located in a gene desert of chr 14, was specific of QTc of *ate*-treated mice. The second SNP hit an independent intergenic segment between *Rragc* and *Pou3f1* on chr 4 and was specific of QTc measured under *iso10* stimulation (Figure 1). The same position was suggestive in the scan of non-corrected QT values. Independent evidence supporting a possible relationship between these hits and ECG values, whether from human GWAs, mutant mice, other animal models or functional studies is lacking but microarrays detected *Rragc* expression in cardiac tissue.

### 2. GWAS of drug effects

With the exception of BW, all phenotypes were measured in separate animals for each drug treatment. Thus the values of the pharmacological effects of *ate* or *iso* on a particular trait were not available per individual mouse. Since EMMA works best when using trait values of several (typically five to ten) replicates per strain but has less power to analyze strain means [20], we scanned for variants underlying trait responsiveness using inferred individual values of *ate-* and *iso10*-mediated effects. These values were generated by computing, for each trait and treated mouse, the difference or the fold change between the value measured in that individual and the average value measured in the *ctr* mice of the matching strain. The effects of *iso10 vs ate* were computed following a similar scheme.

**Figure 3:**
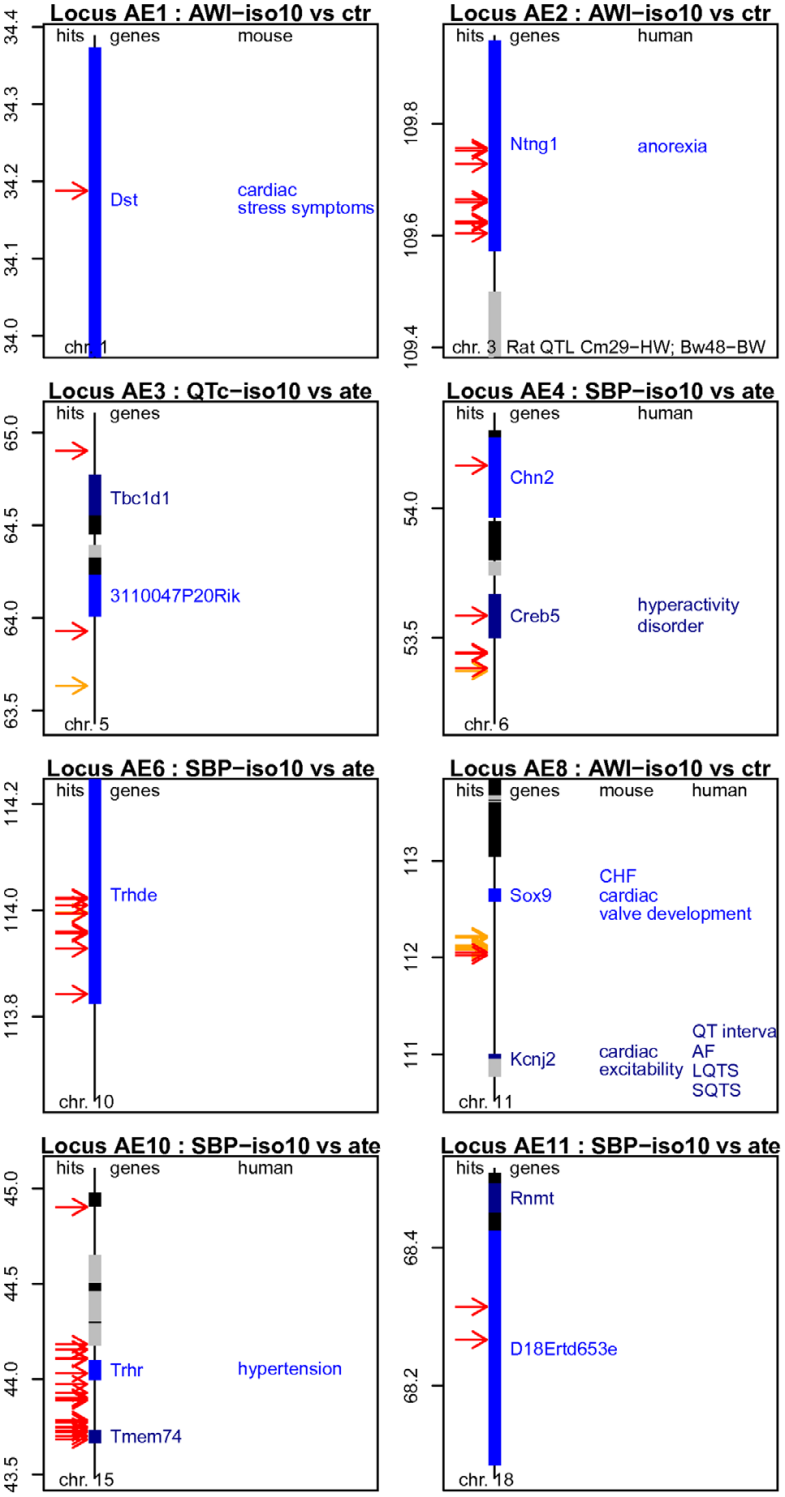
Synoptic maps of the top loci associated with the effects of *iso* and *ate*. The genetic maps of eight loci are presented and annotated as described in the legend of Figure 1. CHF: congestive heart failure; AF: atrial fibrillation; LQTS: long QT syndrome.

Altogether, 100 genome-wide scans were analyzed for the pharmacological effects (Figures S5, S6, S7 and S8): 42 produced no hit above suggestive significance and 25 were rejected upon inspections of the QQ-plots and/or phenotypic variances (Material and Methods). The remaining 33 experiments pointed at 11 significant and 46 suggestive loci associated with trait responsiveness (Table 2, Table S4). A selection of the most significant results is presented below. These results are described in the paragraphs below, while a summary is presented in Figure 3.

### Responsiveness of traits related to cardiac indices and body weight

Three significant loci, all specifically associated with the responsiveness of cardiac atria to *iso10,* were identified (loci AE1, AE2, AE8, Table 2). For the effects on the other traits, in particular VWI, only a small number of suggestive hits were obtained (Figures S5, S6, S7 and S8, Table S4).

Locus AE1 consisted in a single SNP of slightly inflated significance on chr 1 (Table 2). The primary variant tagged *Dst*, a gene coding for a giant cytoskeletal protein called dystonin. A member of the family of plakin proteins, dystonin is capable of crosslinking the major filament systems in contractile cells. Alternatively spliced and tissue-specific isoforms are expressed in mammalian epithelia, neurons and skeletal muscle [63], [64]. In the heart, *Dst* expression is high (Table S5), the major muscle isoform localizing at the Z-disc, H-zone, sarcolemma and intercalated discs [65]. In mice, dystonin deficiency is associated with *dystonia musculorum*, an inherited sensory neuropathy that often leads to pre-weaning death [66]. In some models, these symptoms are accompanied by increased expression of cardiac stress markers similar to those induced by prolonged stimulation with *iso*, in particular *NppA*, *Myh7* and *Serca2a* [5], [65]. However, it is not known whether *β*-adrenergic stimulation is capable of regulating *Dst* or whether similar pathways affect the physiology of cardiac atria.

Locus AE2 tagged *Ntng1* on chr 3 (Table 2). This 1.2 Mb region was delineated by eight SNPs sharing an identical SDP. *Ntng1* codes for netrin G1, a non-essential protein involved in axon guidance during nervous system development in vertebrates [22], [67]. In adult tissues, *Ntng1* is essentially expressed in the brain, with little to no expression in the heart, as confirmed in the microarrays (Table S5). Variants of human *NTNG1* have been suggestively associated with anorexia [55], while the orthologous rat gene is encompassed within four QTLs for cardiac mass and several others for body and organ weight [23]. Possible relationships between *Ntng1* and trait responses to pharmacological treatments have not been documented.

Locus AE8 spans a ca 1.7 Mb intergenic segment between *Kcnj2* and *Sox9* on chr 11 (Table 2). Based on their respective functions in the heart, both genes appear as attractive candidates. *Kcnj2* codes for Kir2.1, an inwardly rectifying potassium channel expressed in tissues such as skeletal muscle and heart, while *Sox9* codes for a transcription factor involved in chondrogenesis and cardiac valve development [22], [68]. Homozygous disruption of *Kcnj2* demonstrated that Kir2.1 was essential for *I*_K1_, a cardiac current involved in the maintenance of the negative resting potential and excitability of cardiac myocytes [69]. Associated phenotypes included bradycardia and alterations of the waveform of action potentials and the firing rate of myocytes [69]. In cultured cardiac myocytes, *I*_K1_ is inhibited by *iso* [70], [71]. Co-immunoprecipitation assays further showed that Kir2.1, *β*_1_-adrenergic receptors and at least two other proteins were organized in a macromolecular complex in cardiac myocytes [70]. Further interesting, familial mutations of human *KCNJ2* are linked with atrial fibrillation, long QT syndrome and short QT syndrome, while nearby variants on chr 17 are associated with QT interval [72].

### Responsiveness of systolic blood pressure

Six loci were significantly associated with the responsiveness of SBP to perturbations of the *β*-adrenergic system. These regions were all identified when analyzing the fold changes between values measured under stimulation with *iso10* and those recorded under *ate* (Table 2). Loci AE7 and AE9 were each supported by single markers with inflated *p*-values (Table 2). Of the remaining four segments, loci AE6 and AE11 were tagged by markers with identical SDPs, suggesting that one or the other locus might be a false positive.

Notwithstanding these considerations, at least two loci specifically pointed at genes belonging to the same functional pathway: locus AE6 for *Trhde* on chr 10 and locus AE10 for *Trhr* on chr 15. Locus AE6 spans 1.8 Mb, with all underlying markers residing within introns of *Trhde*. Locus AE10 covers eight genes within a 1.2 Mb segment; two of the underlying variants are located in introns of *Trhr*, while the remaining 25 SNPs are intergenic (Figures S5, S6, S7 and S8, Table S4). Of the seven, respectively eight strains carrying the minor allele at each locus, five are identical, indicating that both regions are in partial but not complete linkage disequilibrium (data not shown). Mice with the minor alleles tended to exhibit distinct blood pressure values between the *ate* and *iso10* treatments, while the differences were more marginal in the other strains (Figure S10 and [9]).

*Trhr* codes for the thyrotropin-releasing hormone (Trh) receptor, while *Trhde* codes for the Trh-degrading enzyme. Trh represents the most proximal member of the hypothalamic-pituitary-thyroid axis. It is primarily synthesized in the hypothalamus but is also produced in other areas of the brain and in non-neuronal tissues such as the gastrointestinal tract, the heart and reproductive organs. Trh functions as a tripeptide hormone that maintains thyroid hormone homeostasis via regulation of thyroid-stimulating hormone production and secretion. It further controls the release of other hormones, such as prolactin, growth hormone, vasopressin, and insulin. Trh exerts direct roles in cardiovascular physiology in general, and on blood pressure in particular [73]. Thus, administration of Trh to rats triggers dose-dependent increase of SBP, HR and cardiac contractility [74], [75]. The preoptic area (POA) of the brain, a region crucial for the central regulation of arterial blood pressure, is one of the non-hypothalamic areas that produces Trh [73]. In spontaneously hypertensive rats (SHR), the Trh content of the POA is approximately doubled when compared to normotensive controls. Also, injection of antisense *Trh* oligonucleotide in SHRs decreased Trh levels and normalized SBP [76], establishing Trh of the POA as a key regulator of blood pressure. Trh is also produced locally in the heart, primarily by fibroblasts [75], although in our case, transcript levels detected in cardiac ventricles were low (Table S5). Interestingly, stimulation of cultured cardiac fibroblasts with *iso* enhances *Trh* expression [75], indicating that *Trh* is able to respond to *β*-adrenergic activation.

At this stage, it is well conceivable that genetic variation of *Trhr* and *Trhde* modulates the action of Trh on SBP. In humans, a case-control study of 120 hypertensives and 63 normotensives indeed suggested that polymorphisms of the *TRHR* promoter were associated with essential hypertension [77]. Yet, the mechanisms that link pharmacological perturbations of the *β*-adrenergic system with the Trh pathway remain to be established.

### 3. Validation tests

To substantiate the relevance of the mapped loci further, we explored two complementary strategies. First, we tested whether loci for cardiac traits were enriched in genes expressed in the heart. Second, we searched for replication of the hits mapped by EMMA, using independent datasets of the MPD.

### Cardiac transcriptomics

Cardiac transcript levels were measured in untreated ventricles of C57BL/6J and KK/HlJ hearts by microarrays. Our working hypothesis was that the loci associated with cardiac traits contained more genes expressed in the heart than the rest of the genome. To test for this, we considered the 587 genes located within 500 kb of the SNPs suggestive for cardiac-related traits (i.e. cardiac geometry and indices, HR and ECG values), that were probed by the arrays. 354 (60%) of these genes were expressed in the heart. This percentage was significantly higher than the fraction (55%) of all genes expressed in the same samples (*p* = 0.004, binomial test), indicating that the candidate loci were enriched for genes expressed in cardiac tissues.

### Replication in independent cohorts

The MPD provides a repository of phenotypic traits measured in numerous mouse cohorts [24] that can be used to probe for replication of association hits. For each phenotype with suggestive hits and testable replication cohorts, a dataset was selected within the MPD, with the restriction that it included a minimum of 19 strains with documented mouse HapMap genotypes. When multiple such datasets were available, the one with the closest age match and largest number of strains was chosen. Each dataset selected was submitted to the EMMA server [21], excluding values measured in females and/or in wild-derived strains. It was then checked whether the primary hits passed the 5% significance threshold in the replication study (with corrected inflated/deflated *p*-values). Of the 35 testable loci, eight (23%) were replicated at least once (Table 3). A binomial test showed that this fraction represented a significant enrichment (*p* = 0.0003), as only 5% replication would have been expected by chance.

**Table 3:** Replication studies.

Trait	RD (MPD)	RD Trait	RD ID	RD strainsin EMMA	Strains incommon	Loci, this study		Loci, replicated	
BWS	Lightfoot 1	BW	7553	36	22	2	L1, L2	2	L1, L2
BWE	Tordoff 3	BW	7564	31	21	4	L2, L22, L24, L42	3	L22, L24, L42
AW	Reed 1	HW	7580	24	17	4	L1, L2, L3, L68	2	L1, L2
AWI	Reed 1	%HW/BW	7582	24	17	5	L3, L28, L59, L61, L71	0	
AW/BWS	Reed 1	%HW/BW	7582	24	17	3	L3, L20, L63	0	
VWI	Reed 1	%HW/BW	7582	24	17	1	L43	0	
VW/BWS	Reed 1	%HW/BW	7582	24	17	2	L33, L43	1	L33
SBP	Sugiyama 1	SBP	7205	19	14	1	L17	1	L17
PR	Xing 1	PR	7588	26	16	3	L18, L54, L65	0	
QRS	Xing 1	QRS	7589	26	16	2	L9, L45	0	
QT	Xing 1	QT	7590	26	16	8	L10, L15, L16, L21, L30, L60, L62, L67	1	L16
ST	Xing 1	ST	7591	26	16	9	L16, L19, L40, L52, L57, L62, L64, L67, L69	0	

For each trait listed, the suggestive hits mapped by EMMA were tested using the indicated replication dataset (RD) of the MPD. RD ID: MPD ID number of the trait considered for the replication test; RD strains in EMMA: number of strains of the RD used in EMMA; strains in common: number of strains in common between this study and the RD.

Six of the replicated loci relate to body weight and cardiac indices (Table 3), which further reflects the marked robustness of these traits. Even though the number of testable datasets was more limited for the remaining traits, two other loci could be replicated. The first one consisted in the single marker mapped for SBP (locus 17), which was replicated with the values of Sugiyama1 (Table 3). This variant was immediately adjacent to another two markers, *rs*31667929 and *rs*31572758, that both reached genome-wide significance in the replication analysis but were absent from our set of informative SNPs (data not shown). The other replicated region was locus 16, which was defined by a single marker suggestively associated with the non-corrected QT intervals of *ctr* mice (Table S3). This SNP was located within *Khdrbs2*, a gene coding for an RNA-binding protein of poorly understood function.

## Discussion

The most significant hits identified in this study consist in three candidate loci related to cardiac and body weight, three loci for electrocardiographic (ECG) parameters, two loci for the susceptibility of atrial weight index to *iso*, four loci for the susceptibility of SBP to perturbations of the *β*-adrenergic system, and one locus for the responsiveness of QTc. An additional 60 loci were suggestive for one or the other phenotype, while 46 were suggestive for one or the other effect of the treatments. Apart from the many apparent correlations with data extracted from the literature and other databases, we are confident that the mapped loci are reliable candidates for two other reasons: first, the hits suggestively associated with cardiac-related traits tagged genomic regions preferentially enriched in genes expressed in the heart (*p* = 0.004); second, a significant fraction (23%; *p* = 0.0003) of the testable candidate markers was replicated with independent publicly-available datasets. To discuss these results further, the following points need to be considered.

One of the major challenges of association studies in general and of *in silico* mapping in particular is to discern true from false positives without losing (too many) false negatives. By using trait values of eight to ten biological replicates for each phenotype as well as applying a stringent filter to control for missing alleles, conservative thresholds to control for multiple hypotheses testing, and EMMA to correct for population structure and genetic relatedness of the mouse strains, we greatly reduced the number of spurious positive associations. Quality controls, in particular QQ-plots and careful inspections of the patterns of allelic distributions at the top hits further restricted the number of likely false positives.

A significant fraction of the loci identified for the 27 phenotypes was not specific of trait values measured under a single drug treatment but often overlapped across several if not all scans produced for the given trait. This was concordant with our previous observation that robust phenotypes (i.e. those relating to body and to a lesser extent cardiac weight) were marginally affected by *ate* and *iso* [9]. In contrast, most of the loci for ECG values were significant only with respect to a specific drug treatment.

Since the phenotypes were measured in separate cohorts for each drug treatment, individual values of the pharmacological effects of *ate* or *iso* were not available but had to be inferred. This said, it is important to emphasize that measuring pharmacological effects in hundreds of individuals may not be easily implementable for invasive phenotypes such as heart weight. One may argue that the approximations are adding experimental noise to the mapped loci, potentially increasing the number of false positives. However, we note that the vast majority of the hits associated with the effects of *iso10 vs ate* treatments (i.e. those for which the values of trait variance were often the largest) were also retrieved when analyzing the reverse *ate vs iso10* combinations. Had the computed values been noisy, then these results would have been difficult to replicate. Notwithstanding these limitations, our design produced an attractive list of candidate genes. The identification of at least two members of the hypothalamic-pituitary-thyroid axis as putative modulators of the responsiveness of SBP to perturbations of the *β*-adrenergic system appears particularly relevant. Indeed it seems rather unlikely that such a result would emerge by pure chance. As mentioned in the corresponding chapter, the underlying markers seem to discriminate between “SBP-responsive” and “SBP non-responsive” strains for the considered *ate* and *iso10* treatments. However, we reported earlier that most of the 22 mouse lines reacted to a ten-fold lower dose of *iso* (i.e. *iso1*) by reducing SBP [9]. Therefore, none of these strains can be considered as a general non-responder. Additional investigations will be required to dissect the underlying mechanisms further.

Unlike other *β*-blockers, *ate* undergoes little or no metabolism in the liver and is primarily eliminated unchanged by the kidneys [78], [79]. In contrast, *iso* is metabolized within minutes into inactive metabolites by the liver catechol-O-methyltransferase (COMT) [80]. Since the 22 strains carry a single, identical *Comt* haplotype, the pharmacokinetics of both *ate* and *iso* is expected to be only marginally influenced by genetic variation of metabolizing enzymes. This seems well supported by the absence of genes involved in drug metabolism in our list of candidates. Along similar lines, we note that the 22 strains carry the same *Adrb1* haplotype, *Adrb1* coding for the main subtype of *β*-adrenergic receptors in the heart.

On the principle, it is well acknowledged that computational association mapping in inbred mice provides high-resolution mapping. However, the power of this strategy greatly depends on the size and genetic diversity (recombination) of the chosen strains, the complexity of the trait studied, and the frequency of the underlying variants [20], [21], [81]. A panel such as our GRP is well suited to detect loci of strong genetic effects for traits of moderate complexity but has little power to uncover variants with small effect size. This is well illustrated by the relatively modest number of hits identified, that rarely exceeded a single putative locus in any genome-wide scan. Our attempts at analyzing modular phenotypes, that combined multiple traits and/or treatment conditions, did not improve this general outcome (data not shown). Thus, not only the measured traits but also the effects of the drugs are likely governed by a complex architecture of genetic variants. Our results also fit with the notion that the whole strategy is well designed to map common variants, with MAFs of the top markers typically ranging from 0.3 to 0.5.

High-resolution mapping of the mouse phylogeny recently established that the genomes of classical inbred lines were overwhelmingly of *Mus musculus domesticus* origin, with limited contributions from *Mus musculus musculus* and *Mus musculus castaneus* subspecies [82], [83]. In the present set of strains, this translates into genomes being on average of 94.5% *M. m. domesticus* origin, while the remaining fraction is composed of an estimated 5.2% *M. m. musculus* and 0.3% *M. m. castaneus* haplotypes, respectively. There is also a strong bias toward multiple lines sharing the same *M. m. musculus* haplotype in some regions [82]. Reduced genetic diversity precludes the identification of the majority of the genetic determinants existing in natural populations and likely accounts for a large part to the moderate number of loci identified. Conversely, it may further explain the apparent large portion of the phenotypic variance explained by our candidate loci, even for otherwise naturally complex traits.

At this stage, it would be unreasonable to expect that the identified loci are the causal variants and thus should be confirmed by genotyping. Indeed, the mouse HapMap (ca 80¢000 SNPs) does not contain all of the genetic variation between the strains, which, according to the latest figures, amounts to ca 4 mio SNPs per classical inbred genome [83]. Because linkage disequilibrium (LD) regions in these mice span in the range of a megabase or more [21], [83], it is likely that hundreds of linked variants exist in any implicated region. For similar reasons, discussing the identity of putative candidate genes in more details than what is provided with the results would be too premature and clearly speculative.

Even in inbred mice, mutations naturally occur at a certain rate. For the reasons mentioned above, we did not perform confirmatory genotyping and thus cannot formally exclude that one or the other marker mapped in the phenotyped animals differs from that reported in the HapMap database. While chances that such events would precisely affect the reported candidates are low, we also reasoned that the amount of genetic differences between a phenotyped animal and the genotyped animal of the same strain is anyway several orders of magnitude smaller than the genetic differences between the strains. Moreover, since many of the reported loci are supported by more than a single SNP, it seems unlikely that multiple mutation events (or genotyping errors) would simultaneously affect multiple consecutive variants in any given strain. For these reasons, we believe that natural mutation events have little impact on our general conclusions.

In summary, we have three complementary pieces of evidence in favor of the correctness of our results: first, looking at external data overlapping with our studies, we generally observed consistent behavior. Second, among the candidate genes associated with the cardiac-related traits, there is a significant enrichment for genes expressed in the heart. Third, we have accumulated a number of associations pointing to genes that have previously been implicated in cardiac functioning or whose molecular function makes such a role very plausible. Nevertheless, it is important to emphasize that further independent studies will be needed to replicate and refine the loci we identified, to map the underlying causative genes and variants, and to functionally validate them. Future experiments may include more comprehensive expression profiling across the strains and treatments, as well as properly designed replication studies. To improve statistical power and mapping resolution, one may also consider complementing the panel of inbred strains with well-chosen informative lines such as those of the Hybrid Mouse Diversity Panel [81], the Collaborative Cross [84] or wild-derived strains [82].

Knowing which genes are involved in drug response is a key step to understand the biology of the measured traits and associated diseases. Translating these findings into humans can potentially provide valuable insights into the nature of the genes and pathways that modulate inter-individual response variability to therapies with *β*-blockers or *β*-agonists. A more detailed understanding of these determinants and their relationships will ultimately lead to new or improved strategies for personalized treatment in patients suffering from cardiovascular disorders.

## Materials and Methods

### Ethics Statement

All animal procedures described previously [9] had been approved by the "Service de la consommation et des affaires vétérinaires du canton de Vaud" (authorization n° 1649; http://www.vd.ch/fr/autorites/departements/dse/consommation-et-affaires-veterinaires/) and were performed in accordance with the National Institutes of Health (NIH) guidelines for the care and use of laboratory animals.

### Phenotypes

The details of the phenotypic dataset were described previously [9]. The measured trait values are also available through the Mouse Phenome Database [24], a web portal that provides access to a large collection of datasets and online tools to visualize and investigate mouse phenotypes. To generate the individual values of trait responsiveness to *ate* and *iso*, we computed for each trait and treated mouse (*ate*, *iso10*), the difference (for Qamp, QRSarea, Samp) or the fold change (FC; all other phenotypes) between the value measured in that individual and the average value measured in the *ctr* mice of the matching strain.

### EMMA

EMMA scans [20] were produced using non-transformed trait values and 79922 informative SNPs of the mouse HapMap resource [21]. Strain C3H/HeOuJ was excluded from the original dataset due to low genetic coverage. SNPs were declared informative if allele information was known in at least 20 of the 22 strains and a minimum of four strains carried the minor allele at the specified position. SNPs with lower minor allele frequencies (MAFs) and/or missing genotypes were indeed likely to generate spurious associations, in particular since, for rare variants, identical SDPs could often be attributed to more than one chromosomal location.

For each phenotype, four EMMA scans were generated, each one using trait values measured in a single treatment condition (*ctr*, *ate*, *iso1* and *iso10*). An exception pertains to the analysis of BWS for which a single scan integrated the values of all mice.

The significance of the associations was evaluated with an *F*-test (REMLt) and conservative thresholds of significance were set using the Bonferroni correction for multiple hypotheses testing. The distributions of the obtained *versus* expected genome-wide *p*-values were assessed by QQ-plots. Hits were declared nominally significant for *p*-values≤10^−8^ and suggestive for *p*-values ranging from 10^−6^ to 10^−8^. The former corresponds to a strict Bonferroni threshold, as -log_10_(0.05/(47999*89)) = 7.9, where 47999 is the number of SDPs covered by the 79922 informative SNPs and 89 is the number of GWAS that passed QQ-plot inspection. Since such a stringent correction is likely to miss many true positives, we also reported the results at an intermediate significance range. Its upper bound of 10^−6^ was motivated by estimating the number of truly independent tests. To this end we used the number of eigenvectors required to explain 99.5% of the variation of the data [85], which turned out to be 16 for our phenotypes. In order to estimate the number of independent genotypes we ran EMMA on the permuted phenotypes and looking at the smallest *p*-value (median *p*-value across the permutations = 4.914×10^−4^), we inferred the number of independent tests per phenotype to be its inverse (i.e. 2035). The effective total number of tests is thus equal to 16×2035, while the corresponding threshold is -log_10_(0.05/(16×2035)) = 5.8.

Whenever possible, the associations obtained as significant at positions with incomplete SDPs were manually imputed and corrected, using information of a compatible nearby variant with a fully known allele set.

The percentage of phenotypic variance explained at each locus was calculated with EMMA. Approximated values of the effects of the treatments on 25 of the 27 traits were analyzed using the same settings and filters, with the added condition that for each strain *s*, measured phenotype *p_m_* and approximated phenotype *p_a_*, 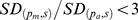. Differences were analyzed as non-transformed values, while FCs were log-transformed prior to running EMMA.

### Microarrays

The 30 to 40% most apical fractions of the cardiac ventricles were excised from C57BL/6J and KK/HlJ mouse hearts (n = 4 per strain), snap-frozen in liquid nitrogen, and stored at −70°C. Tissues were homogenized with a Tissue Lyzer system in the presence of metal beads (Qiagen). Total RNA was extracted using the mirVana isolation kit of Ambion. RNA quality was confirmed on a Bioanalyzer system (Agilent Technologies). Poly-A^+^ RNA purification, cDNA libraries preparation, labeling and hybridization onto Affymetrix Mouse Gene 1.0 ST arrays, as well as washing and scanning of the arrays were performed at the Lausanne Genomics Technologies Facility (LGTF) following standard protocols. Each sample was analyzed on a separate array. All arrays passed standard quality controls. Statistical analyses were performed using the statistical language R (http://www.R-project.org) and the Limma Bioconductor package (http://www.Bioconductor.org). Log_2_ normalized expression signals were calculated from Affymetrix CEL files using the RMA algorithm.

To compute the set of expressed genes, the mixture of two Gaussian functions was fitted to the log expression values, which was bimodal (Figure S9). The first component, peaking at around 2, fitted the non-expressed genes, whereas the second component, peaking at around 6.5, fitted the expressed genes. Expressed genes are those which belong to the second component with at least 95% confidence, i.e., genes that have a log expression value higher than 3.55.

## Supporting Information

Figure S1 **Manhattan and QQ-plots for 27 traits measured in** ***ctr*** **mice.** QQ-plot-based quality control is indicated as “passed” or “failed”.
(PDF)

Figure S2 **Manhattan and QQ-plots for 26 traits measured in** ***ate*** **-treated mice.** QQ-plot-based quality control is indicated as “passed” or “failed”.
(PDF)

Figure S3 **Manhattan and QQ-plots for 26 traits measured in** ***iso1*** **-treated mice.** QQ-plot-based quality control is indicated as “passed” or “failed”.
(PDF)

Figure S4 **Manhattan and QQ-plots for 26 traits measured in** ***iso10*** **-treated mice.** QQ-plot-based quality control is indicated as “passed” or “failed”.
(PDF)

Figure S5 **Manhattan and QQ-plots for the effects of** ***iso10*** **treatment on 25 traits.** QQ-plot-based quality control is indicated as “passed” or failed”. Phenotypes for which any of the differences between an individual trait value and its matching mean strain value exceeded 3 SD are labelled as “var test failed”.
(PDF)

Figure S6 **Manhattan and QQ-plots for the effects of** ***ate*** **treatment on 25 traits.** QQ-plot-based quality control is indicated as “passed” or failed”. Phenotypes for which any of the differences between an individual trait value and its matching mean strain value exceeded 3 SD are labelled as “var test failed”.
(PDF)

Figure S7 **Manhattan and QQ-plots for the effects of** ***iso10 vs ate*** **treatments on 25 traits.** QQ-plot-based quality control is indicated as “passed” or failed”. Phenotypes for which any of the differences between an individual trait value and its matching mean strain value exceeded 3 SD are labelled as “var test failed”.
(PDF)

Figure S8 **Manhattan and QQ-plots for the effects of** ***ate vs iso10*** **treatments on 25 traits.** QQ-plot-based quality control is indicated as “passed” or failed”. Phenotypes for which any of the differences between an individual trait value and its matching mean strain value exceeded 3 SD are labelled as “var test failed”.
(PDF)

Figure S9 **Distribution of cardiac mRNA expression levels as measured by microarrays.** The mixture of two Gaussian functions was fitted to the log expression values. The first component, peaking at around 2, fits the non-expressed genes, whereas the second component, peaking at around 6.5, fits the expressed genes. Non-expressed genes are those which belong to the first component with at least 95% confidence, i.e., those genes that have a log expression value less than 2.33 (red line).
(PDF)

Figure S10 **Boxplots showing the SBP values of 22 strains treated with** ***ate*** **(in red) and** ***iso10*** **(in blue).** Strains are segregated according to their genotype at marker *rs29354390* in *Trhrde* (locus AE6).
(PDF)

Figure S11 **Analysis flowchart**. This flowchart summarizes the analysis of the 27 phenotypes measured across the 22 inbred strains. Details referring to the boxes shaded in grey have been published previously [9]. These phenotypes are also freely available through the Mouse Phenome Database (project Maurer1; http://phenome.jax.org/). Analyses of the drug responses were performed following a similar scheme, except that the values were not physically measured in individual mice but approximated, as detailed in the main text.
(PDF)

Table S1 **Phenotypes and abbreviations used in this study.**
(XLS)

Table S2 **Heritabilities (** ***h^2^*** **) for 27 traits and trait responsiveness to** ***ate*** **and** ***iso*** **, as computed by EMMA.** The values of the genetic variance components for the various traits and responses were computed by fitting EMMA without any SNP but just using the kinship matrix [20]. On average, the heritabilities for the effects of *iso10* or *iso10 vs ate* were ca 10 to15% lower than those of the measured traits, indicating that a significant fraction of trait responsiveness to these treatments was genetically determined. In contrast, the values for the effects of *ate* were lower (typically *h^2^*<0.5), consistent with our previous observation that the phenotypic changes induced by *β*-blockade were moderate for most phenotypes [9]. NA: not applicable; ND: not done.
(XLS)

Table S3 **Suggestive and significant hits mapped for the 27 traits.** For each SNP, the association scores are given as the negative logarithm of the *p*-values calculated in EMMA, across the 105 genome-wide scans (columns K to DK). The data are ranked by position, from chr 1 to chr 17 (rows 5 to 391).
(XLS)

Table S4 **Suggestive and significant hits mapped for the effects of the treatments.** For each SNP, the association scores are given as the negative logarithm of the *p*-values calculated in EMMA across 201 genome-wide scans (columns L to HD). The data are ranked by position, from chr 1 to chr 19 (rows 4 to 217).
(XLS)

Table S5 **Log_2_ normalized cardiac expression values of the main candidate genes.** Transcripts were measured by microarrays in the cardiac ventricles of untreated C57BL/6J and KK/HlJ mice (n = 4 per strain). Values were calculated from Affymetrix CEL files using the RMA algorithm. Data are listed by probeset and are ranked by locus.
(XLS)
