# Correction: Mapping Genetic Variants Associated with *Beta*-Adrenergic Responses in Inbred Mice

**DOI:** 10.1371/annotation/16d5801b-2701-4ec9-9d8e-278791a8a26a

**Published:** 2014-01-03

**Authors:** Micha Hersch, Bastian Peter, Hyun Min Kang, Fanny Schüpfer, Hugues Abriel, Thierry Pedrazzini, Eleazar Eskin, Jacques S. Beckmann, Sven Bergmann, Fabienne Maurer

There is an error in the key for Figure 2. The correct key for Figure 2 is:

black dots: iso1

green dots: iso10

blue dots: ctr

red dots: ate

