# Supplementary material for: Mapping Genetic Variants Associated with Beta-Adrenergic Responses in Inbred Mice
Source: PLoS One. 2012 Jul 31;7(7):e41032. doi: 10.1371/journal.pone.0041032 (PMC3409184; doi:10.1371/journal.pone.0041032)
Supplement: Figure S1 — Manhattan and QQ-plots for 27 traits measured in ctr mice. QQ-plot-based quality control is indicated as “passed” or “failed”. (PDF) [file pone.0041032.s001.pdf]

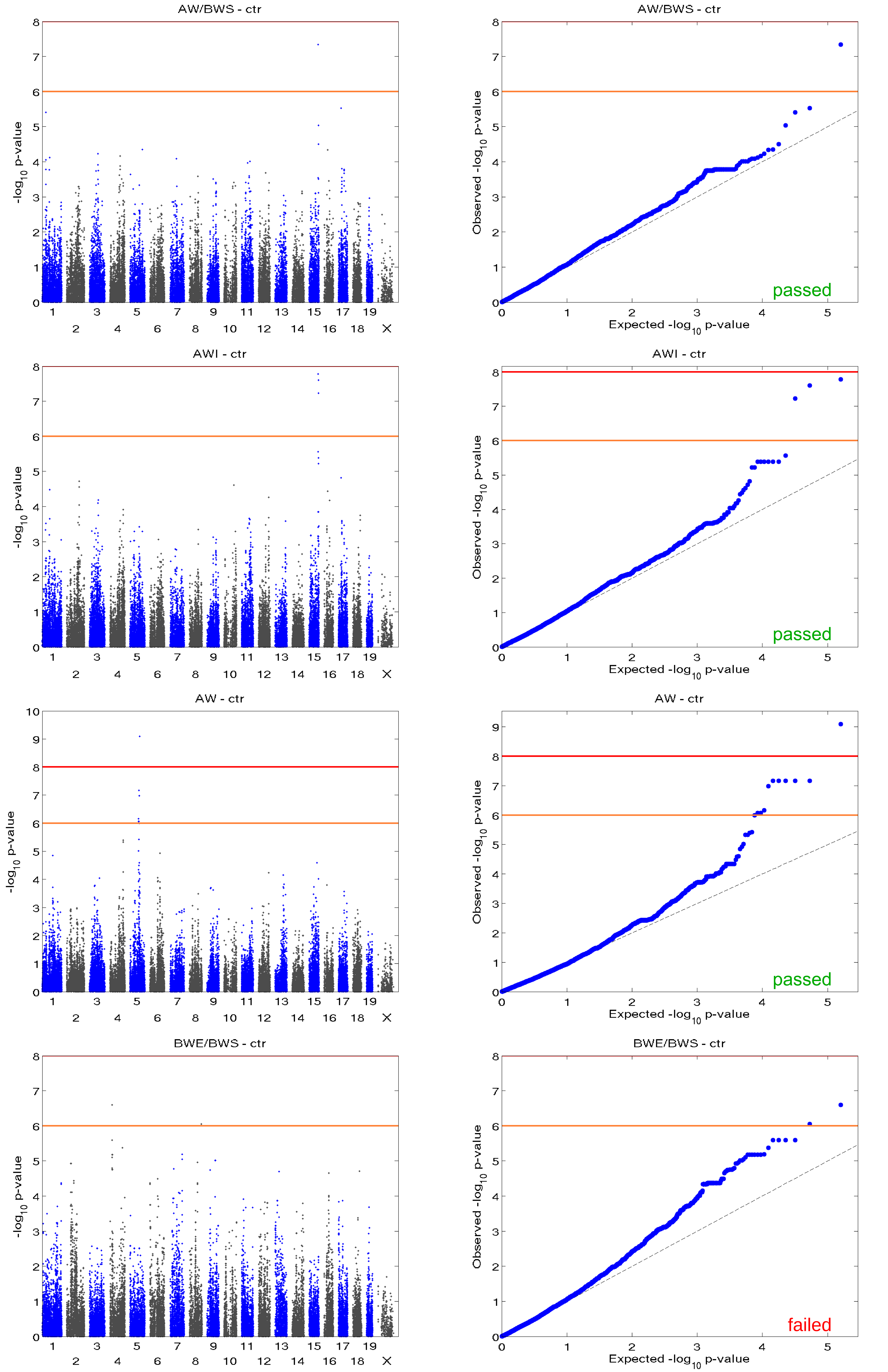

AW/BWS - ctr
AWI - ctr
AW - ctr
BWE/BWS - ctr
$-\log_{10}$ p-value
Observed $-\log_{10}$ p-value
Expected $-\log_{10}$ p-value
passed
passed
passed
failed
1
2
3
4
5
6
7
8
9
10
11
12
13
14
15
16
17
18
19
X

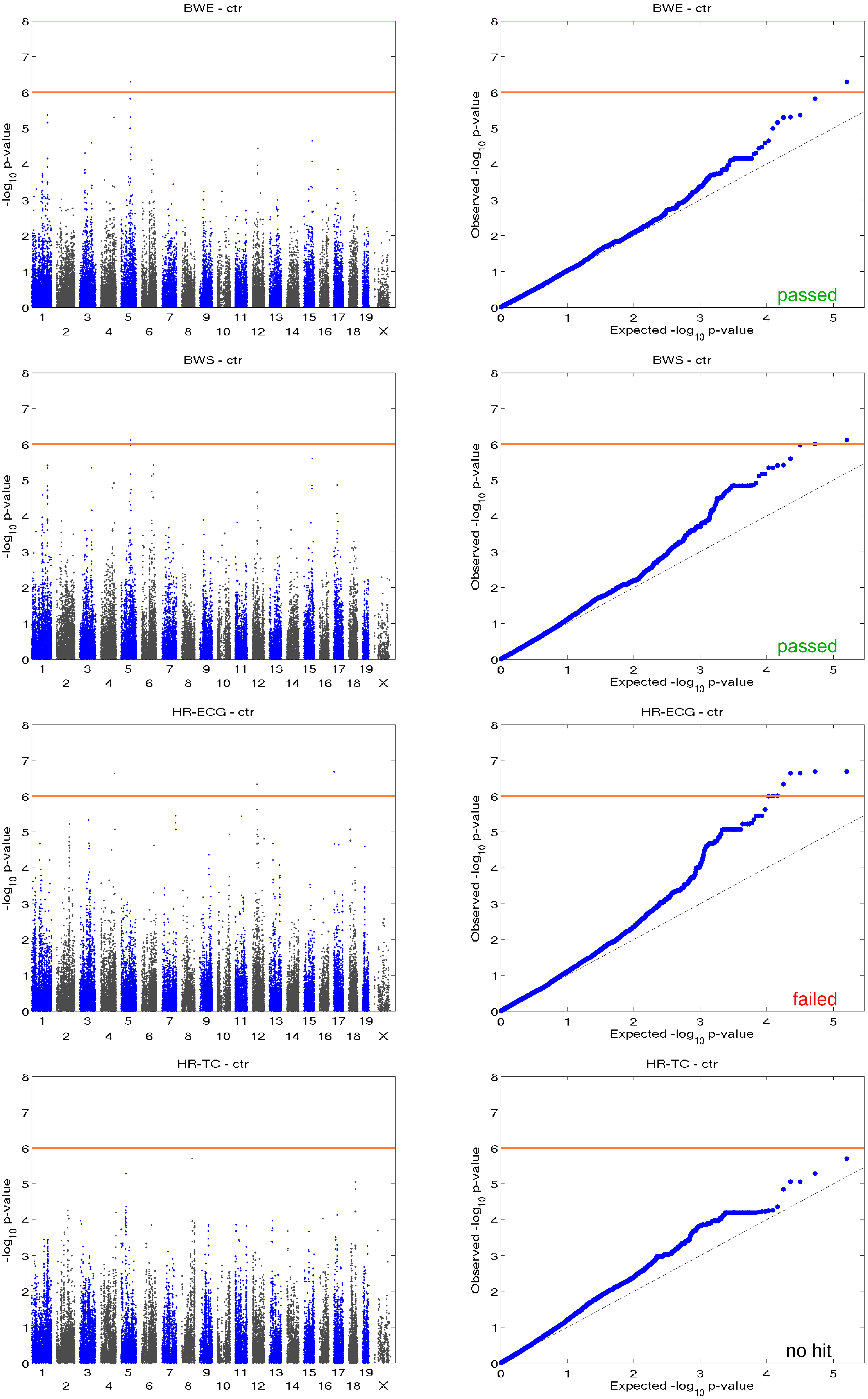
BWE - ctr
BWS - ctr
HR-ECG - ctr
HR-TC - ctr
-log10 p-value
Observed -log10 p-value
Expected -log10 p-value
1 2 3 4 5 6 7 8 9 10 11 12 13 14 15 16 17 18 19 X
passed
passed
failed
no hit

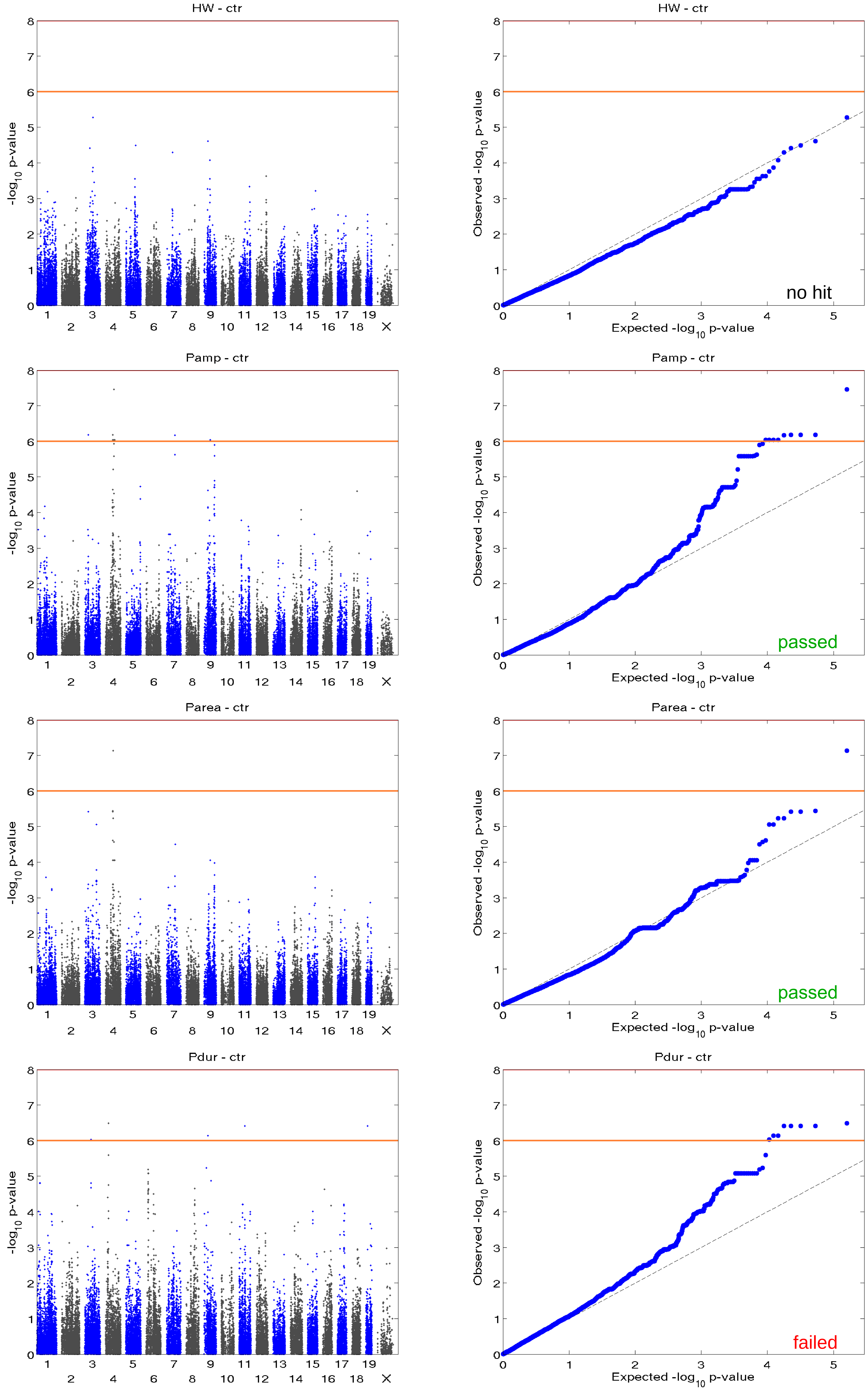

HW - ctr
Pamp - ctr
Parea - ctr
Pdur - ctr
-log10 p-value
Observed -log10 p-value
Expected -log10 p-value
no hit
passed
passed
failed

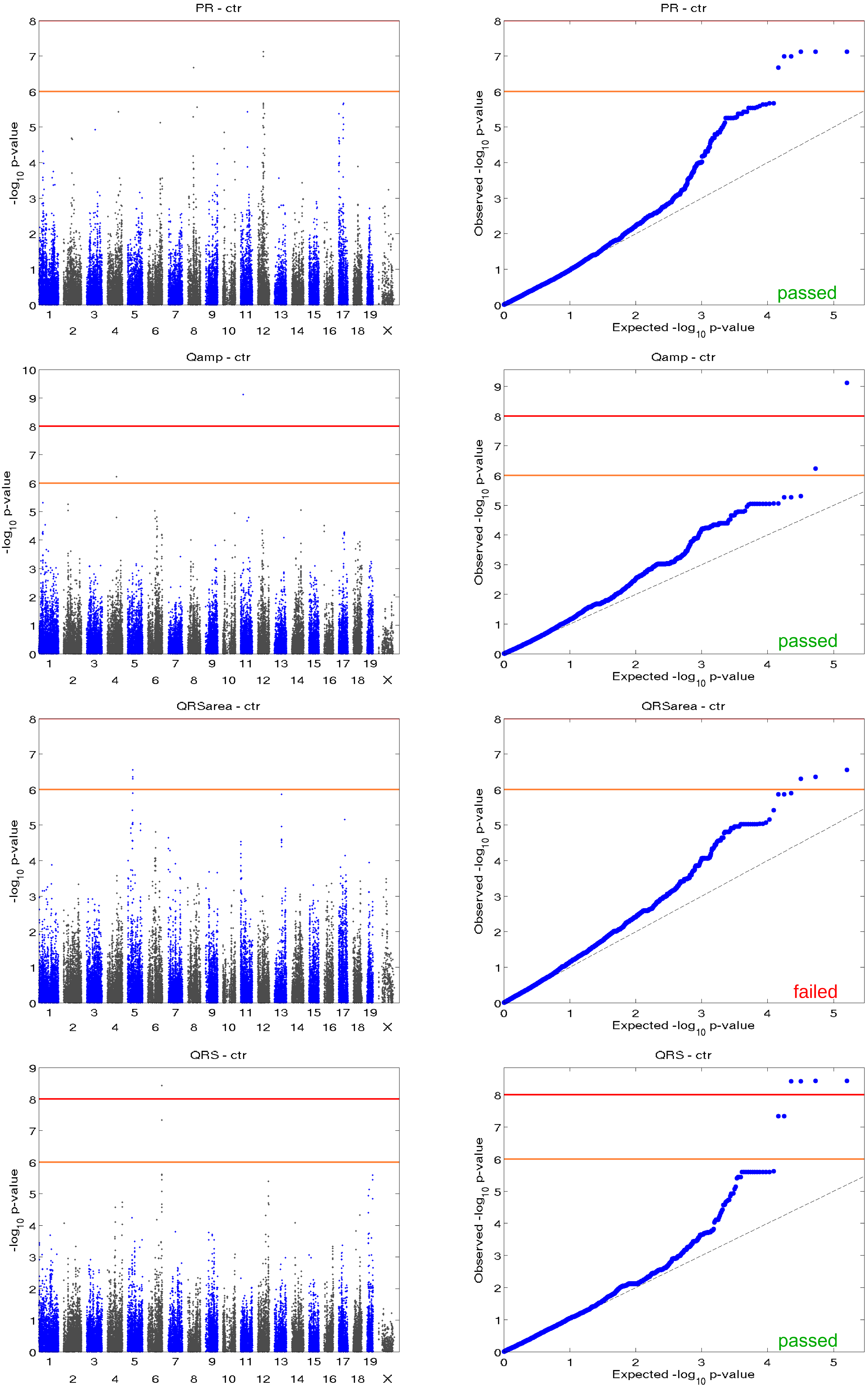
PR - ctr
-log10 p-value
PR - ctr
Observed -log10 p-value
Expected -log10 p-value
passed
Qamp - ctr
-log10 p-value
Qamp - ctr
Observed -log10 p-value
Expected -log10 p-value
passed
QRSarea - ctr
-log10 p-value
QRSarea - ctr
Observed -log10 p-value
Expected -log10 p-value
failed
QRS - ctr
-log10 p-value
QRS - ctr
Observed -log10 p-value
Expected -log10 p-value
passed
1 2 3 4 5 6 7 8 9 10 11 12 13 14 15 16 17 18 19 X

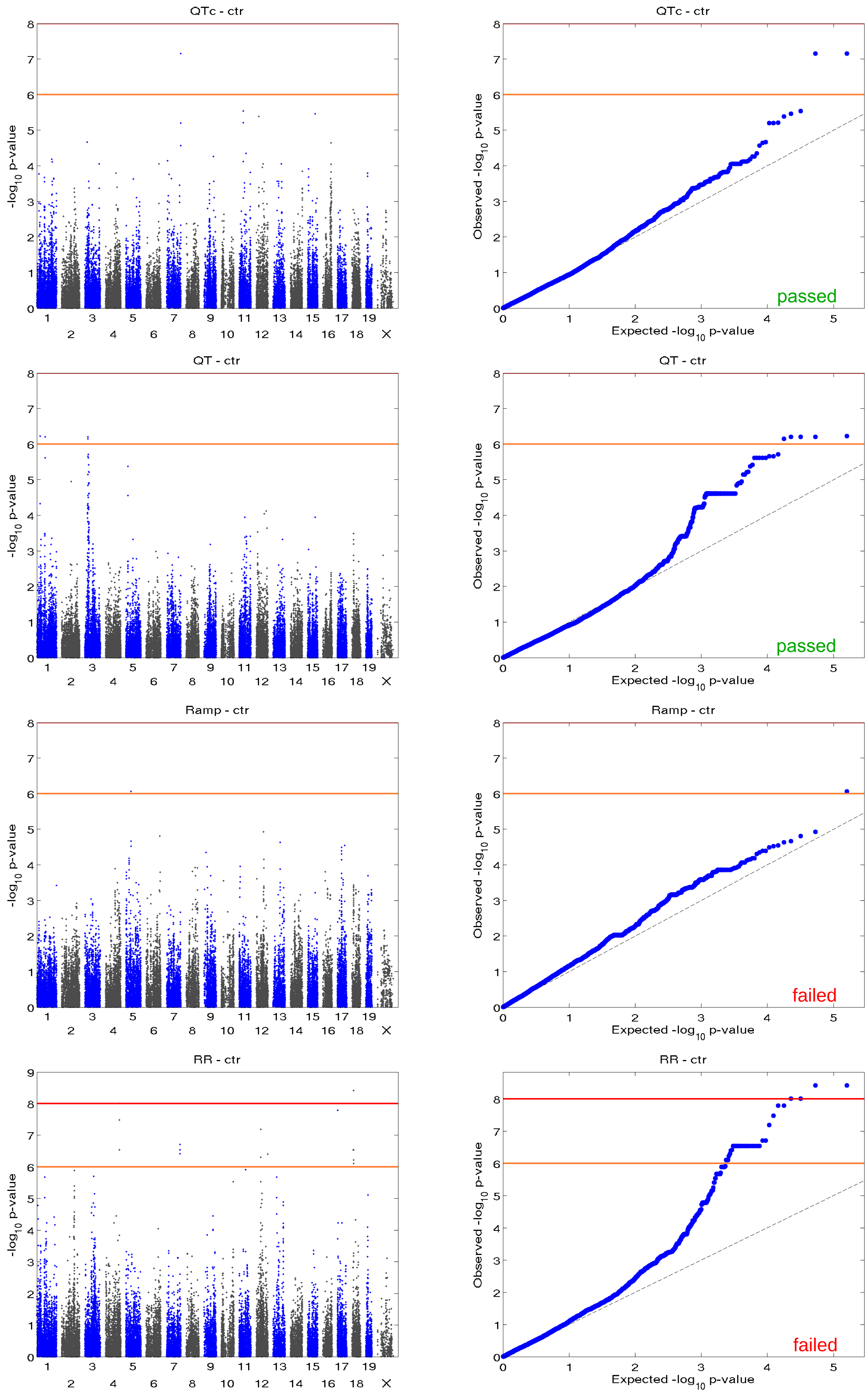
QTc - ctr
QT - ctr
Ramp - ctr
RR - ctr
-log10 p-value
Observed -log10 p-value
Expected -log10 p-value
passed
passed
failed
failed

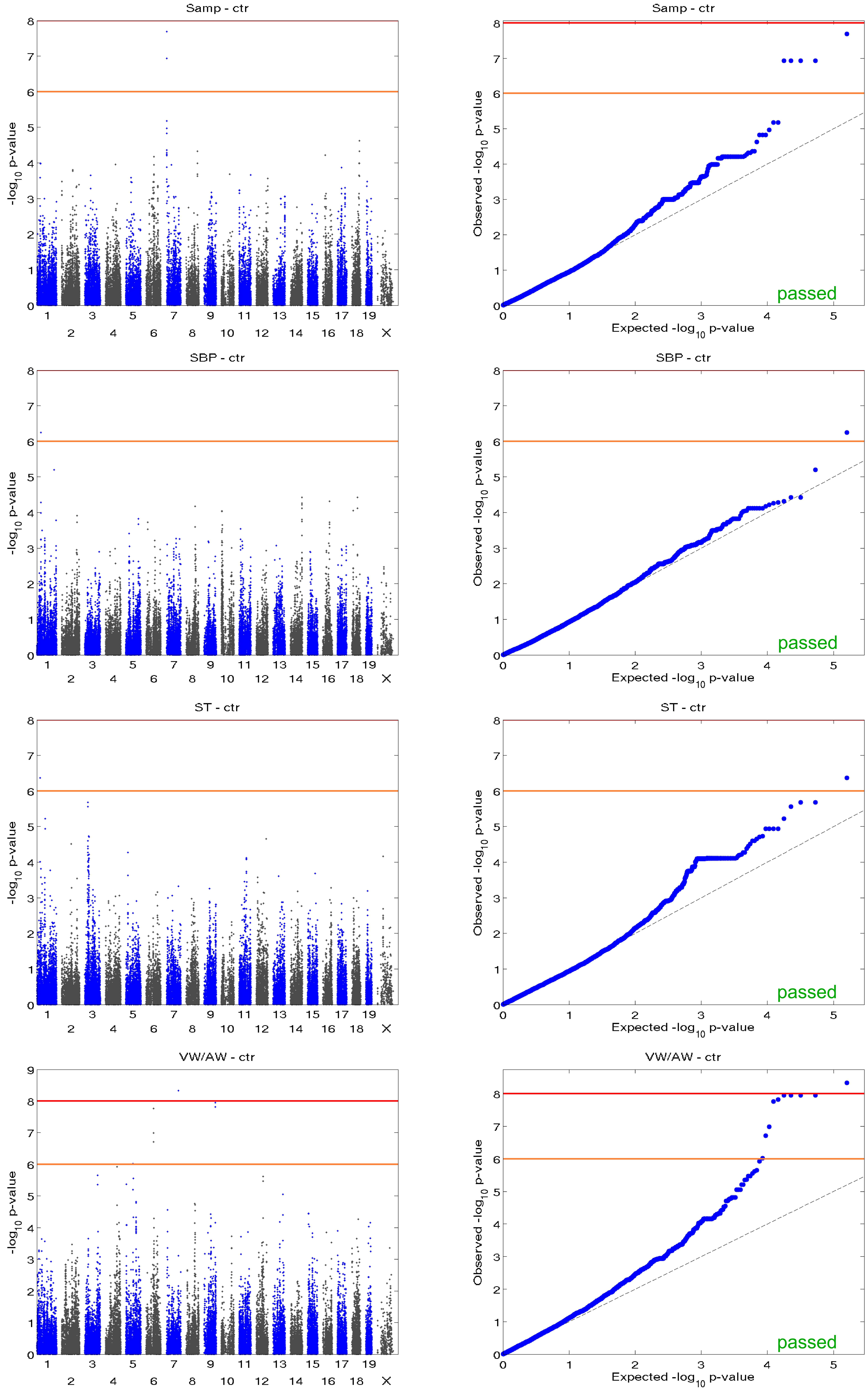
Samp - ctr
-log10 p-value
1 2 3 4 5 6 7 8 9 10 11 12 13 14 15 16 17 18 19 X
Samp - ctr
Observed -log10 p-value
Expected -log10 p-value
passed
SBP - ctr
-log10 p-value
1 2 3 4 5 6 7 8 9 10 11 12 13 14 15 16 17 18 19 X
SBP - ctr
Observed -log10 p-value
Expected -log10 p-value
passed
ST - ctr
-log10 p-value
1 2 3 4 5 6 7 8 9 10 11 12 13 14 15 16 17 18 19 X
ST - ctr
Observed -log10 p-value
Expected -log10 p-value
passed
VW/AW - ctr
-log10 p-value
1 2 3 4 5 6 7 8 9 10 11 12 13 14 15 16 17 18 19 X
VW/AW - ctr
Observed -log10 p-value
Expected -log10 p-value
passed

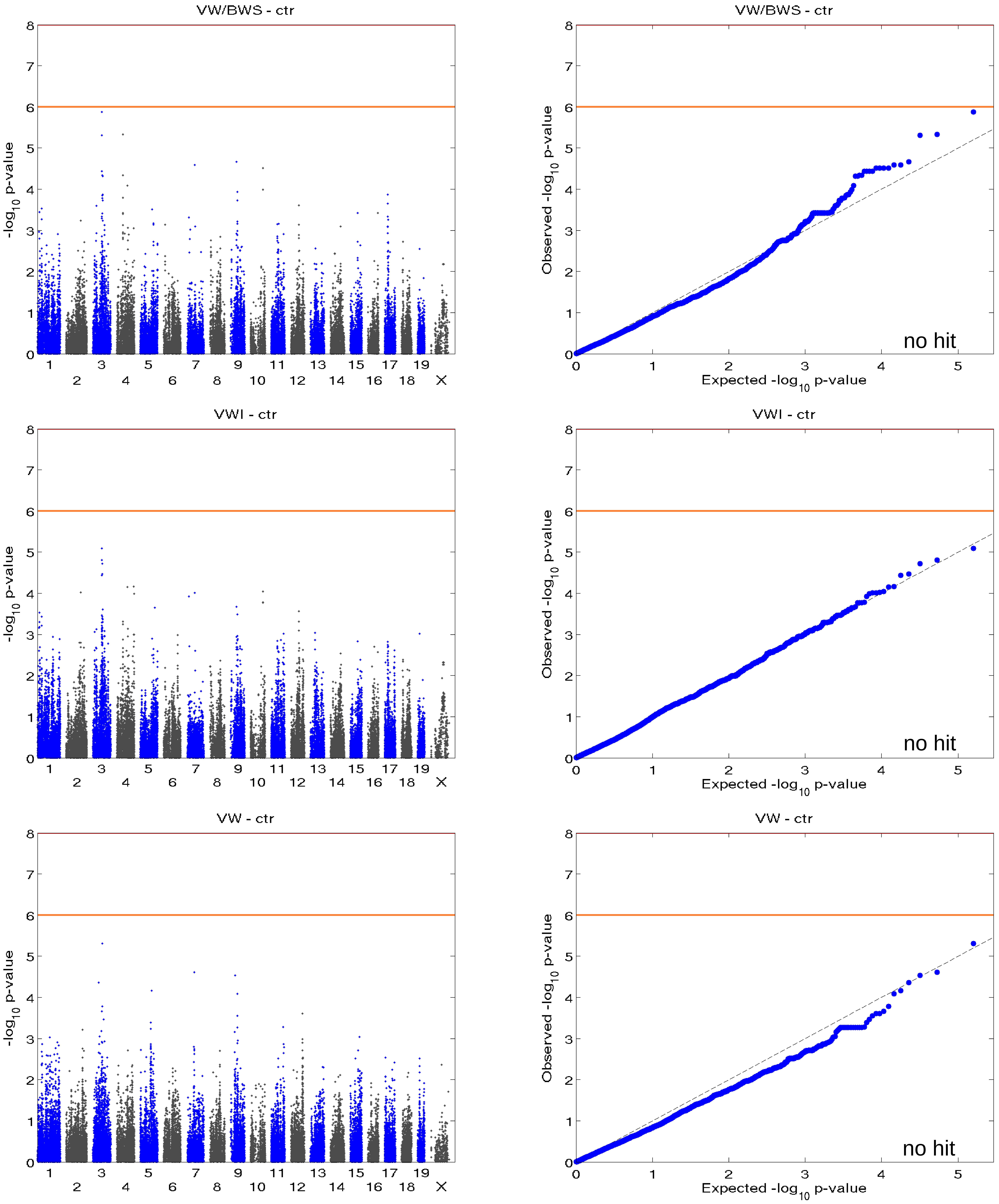
VW/BWS - ctr
-log10 p-value
VW/BWS - ctr
Observed -log10 p-value
Expected -log10 p-value
no hit
VWI - ctr
-log10 p-value
VWI - ctr
Observed -log10 p-value
Expected -log10 p-value
no hit
VW - ctr
-log10 p-value
VW - ctr
Observed -log10 p-value
Expected -log10 p-value
no hit
1 2 3 4 5 6 7 8 9 10 11 12 13 14 15 16 17 18 19 X
