# Supplementary material for: Mapping Genetic Variants Associated with Beta-Adrenergic Responses in Inbred Mice
Source: PLoS One. 2012 Jul 31;7(7):e41032. doi: 10.1371/journal.pone.0041032 (PMC3409184; doi:10.1371/journal.pone.0041032)
Supplement: Figure S2 — Manhattan and QQ-plots for 26 traits measured in ate -treated mice. QQ-plot-based quality control is indicated as “passed” or “failed”. (PDF) [file pone.0041032.s002.pdf]

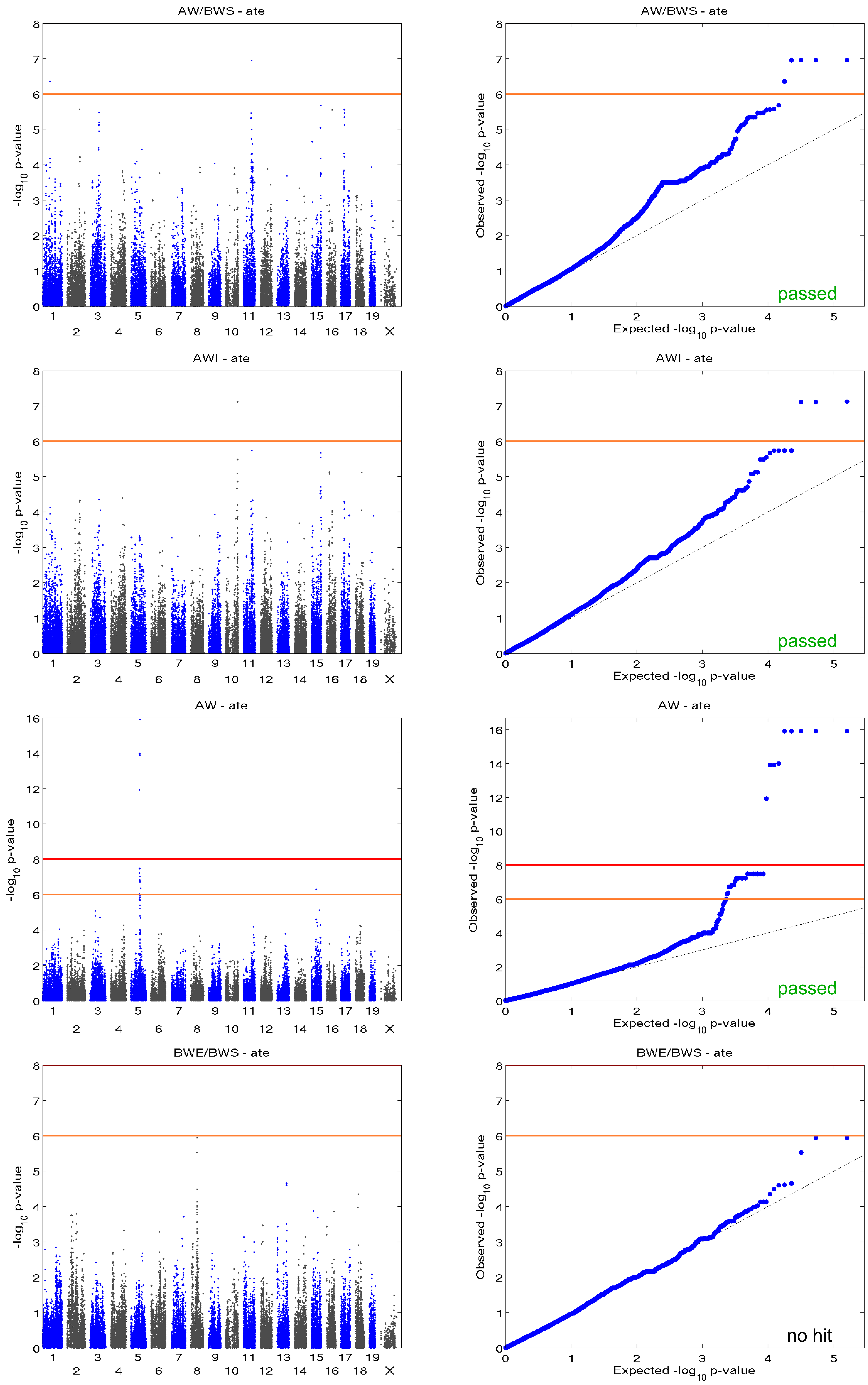

AW/BWS - ate
-log10 p-value
AW/BWS - ate
Observed -log10 p-value
Expected -log10 p-value
passed
AWI - ate
-log10 p-value
AWI - ate
Observed -log10 p-value
Expected -log10 p-value
passed
AW - ate
-log10 p-value
AW - ate
Observed -log10 p-value
Expected -log10 p-value
passed
BWE/BWS - ate
-log10 p-value
BWE/BWS - ate
Observed -log10 p-value
Expected -log10 p-value
no hit
1 2 3 4 5 6 7 8 9 10 11 12 13 14 15 16 17 18 19 X

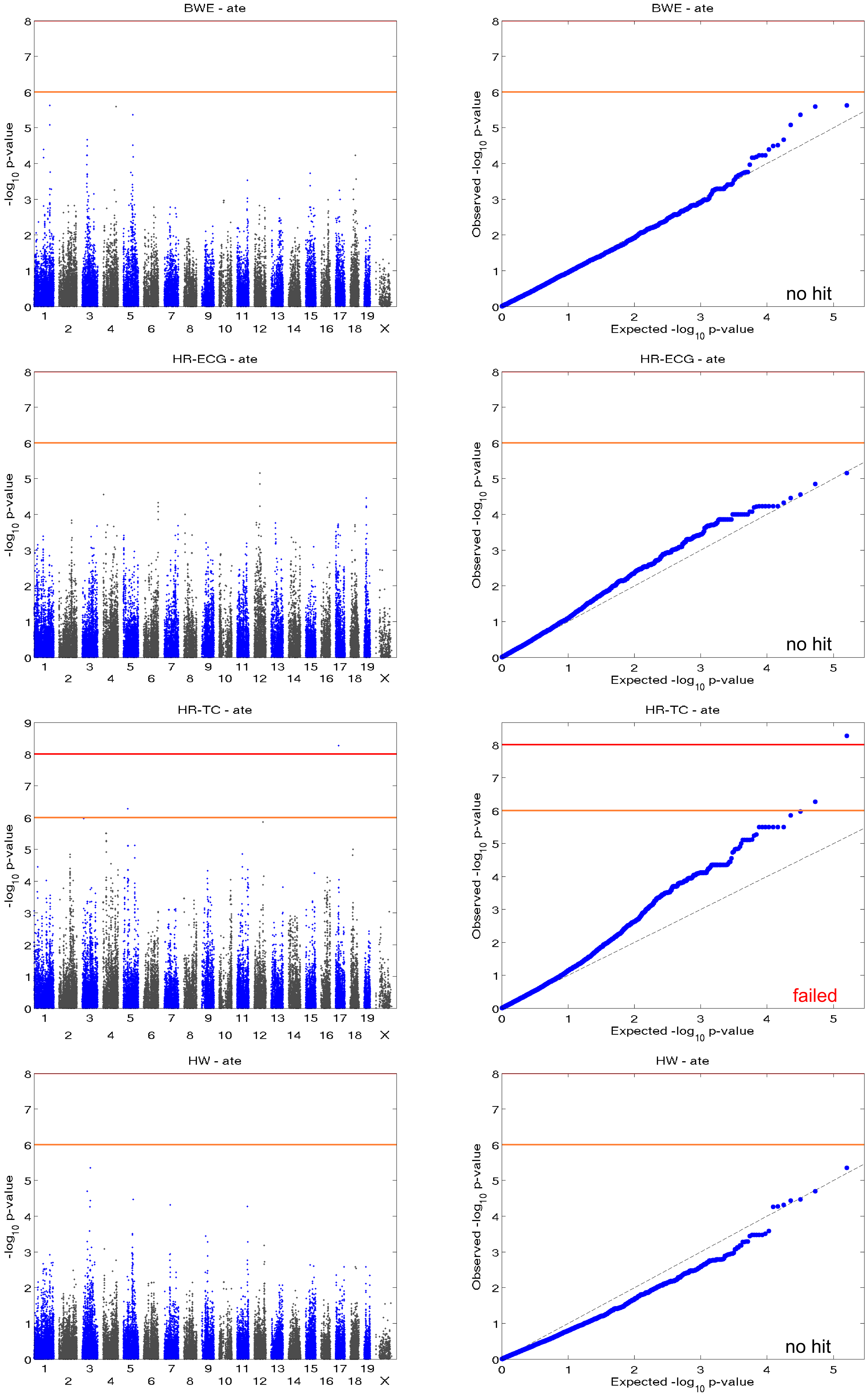

BWE - ate
BWE - ate
-log10 p-value
Observed -log10 p-value
Expected -log10 p-value
no hit
HR-ECG - ate
HR-ECG - ate
no hit
HR-TC - ate
HR-TC - ate
failed
HW - ate
HW - ate
no hit
1 2 3 4 5 6 7 8 9 10 11 12 13 14 15 16 17 18 19 X

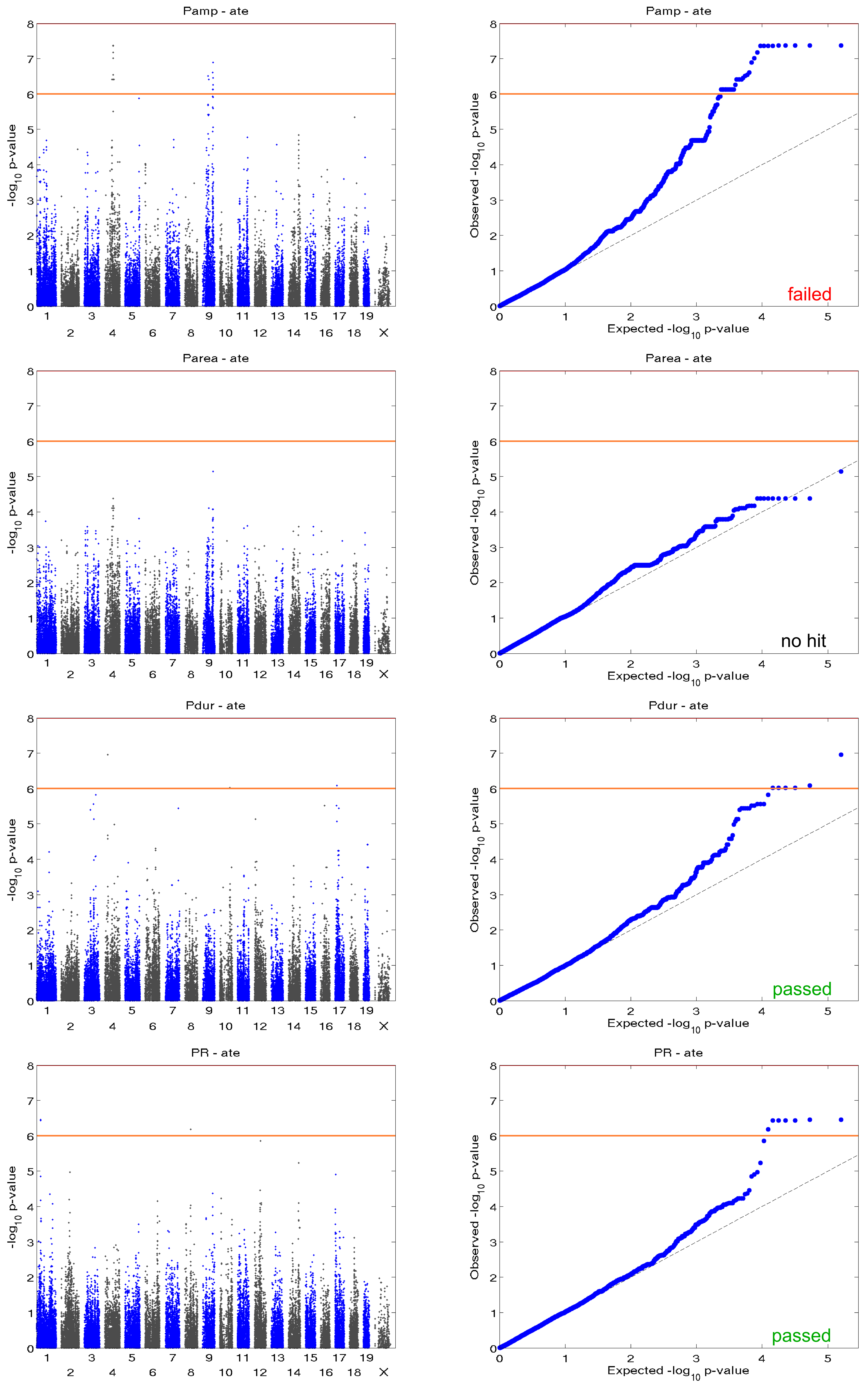

Pamp - ate
-log10 p-value
Pamp - ate
Observed -log10 p-value
Expected -log10 p-value
failed
Parea - ate
-log10 p-value
Parea - ate
Observed -log10 p-value
Expected -log10 p-value
no hit
Pdur - ate
-log10 p-value
Pdur - ate
Observed -log10 p-value
Expected -log10 p-value
passed
PR - ate
-log10 p-value
PR - ate
Observed -log10 p-value
Expected -log10 p-value
passed

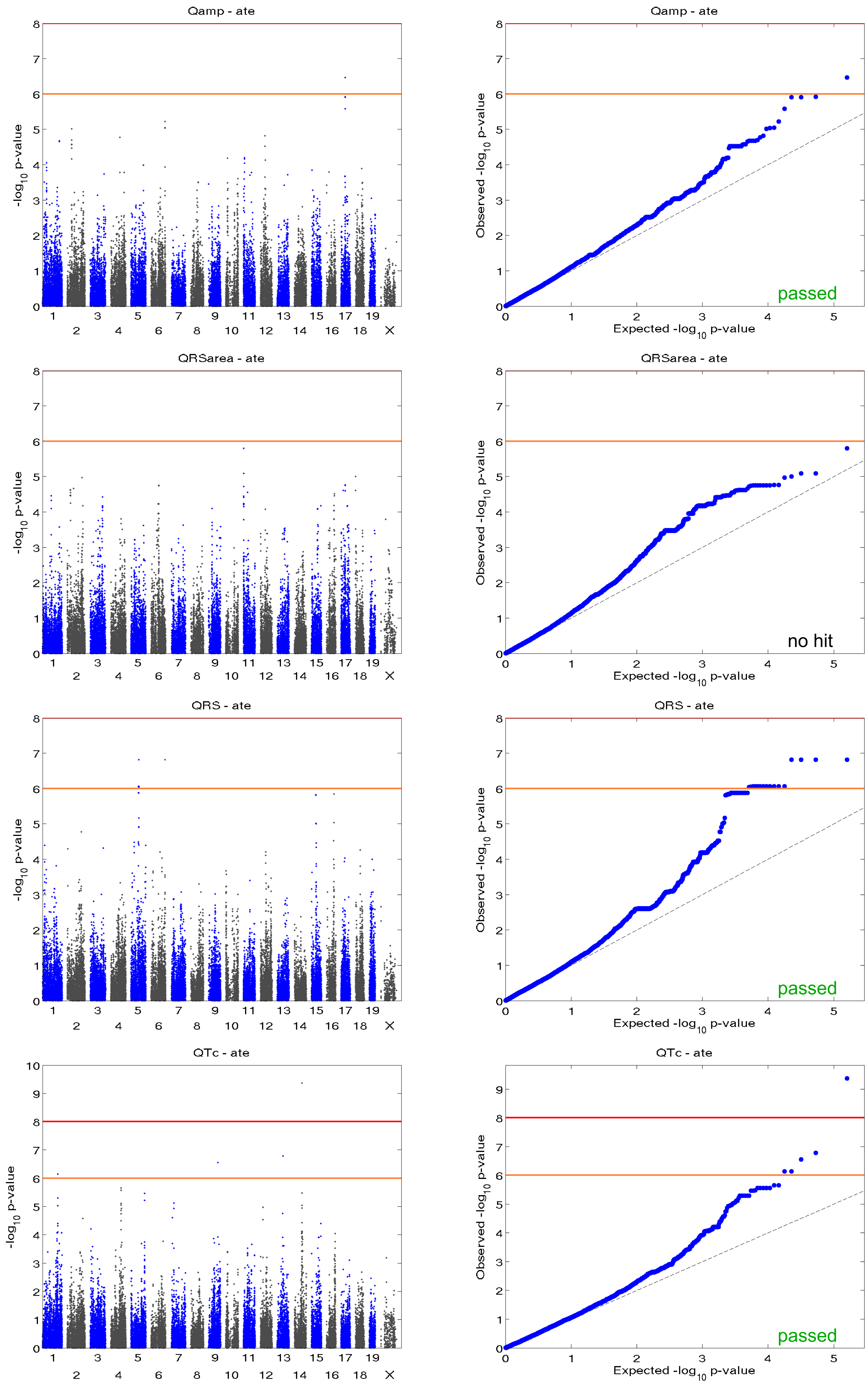

Qamp - ate
-log10 p-value
Qamp - ate
Observed -log10 p-value
Expected -log10 p-value
passed
QRSarea - ate
-log10 p-value
QRSarea - ate
Observed -log10 p-value
Expected -log10 p-value
no hit
QRS - ate
-log10 p-value
QRS - ate
Observed -log10 p-value
Expected -log10 p-value
passed
QTc - ate
-log10 p-value
QTc - ate
Observed -log10 p-value
Expected -log10 p-value
passed

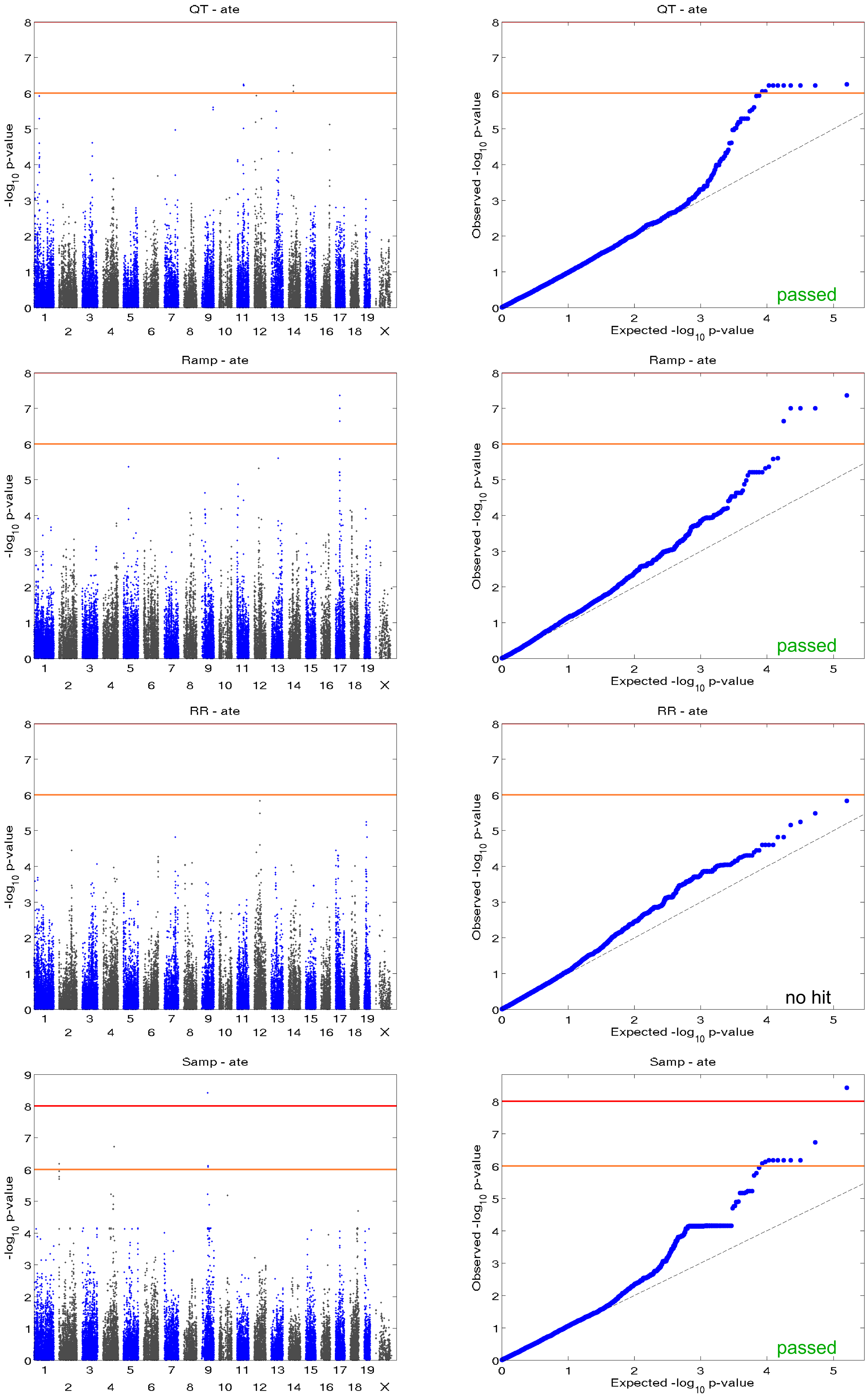

QT - ate
Ramp - ate
RR - ate
Samp - ate
-log10 p-value
Observed -log10 p-value
Expected -log10 p-value
passed
passed
no hit
passed
1 2 3 4 5 6 7 8 9 10 11 12 13 14 15 16 17 18 19 X

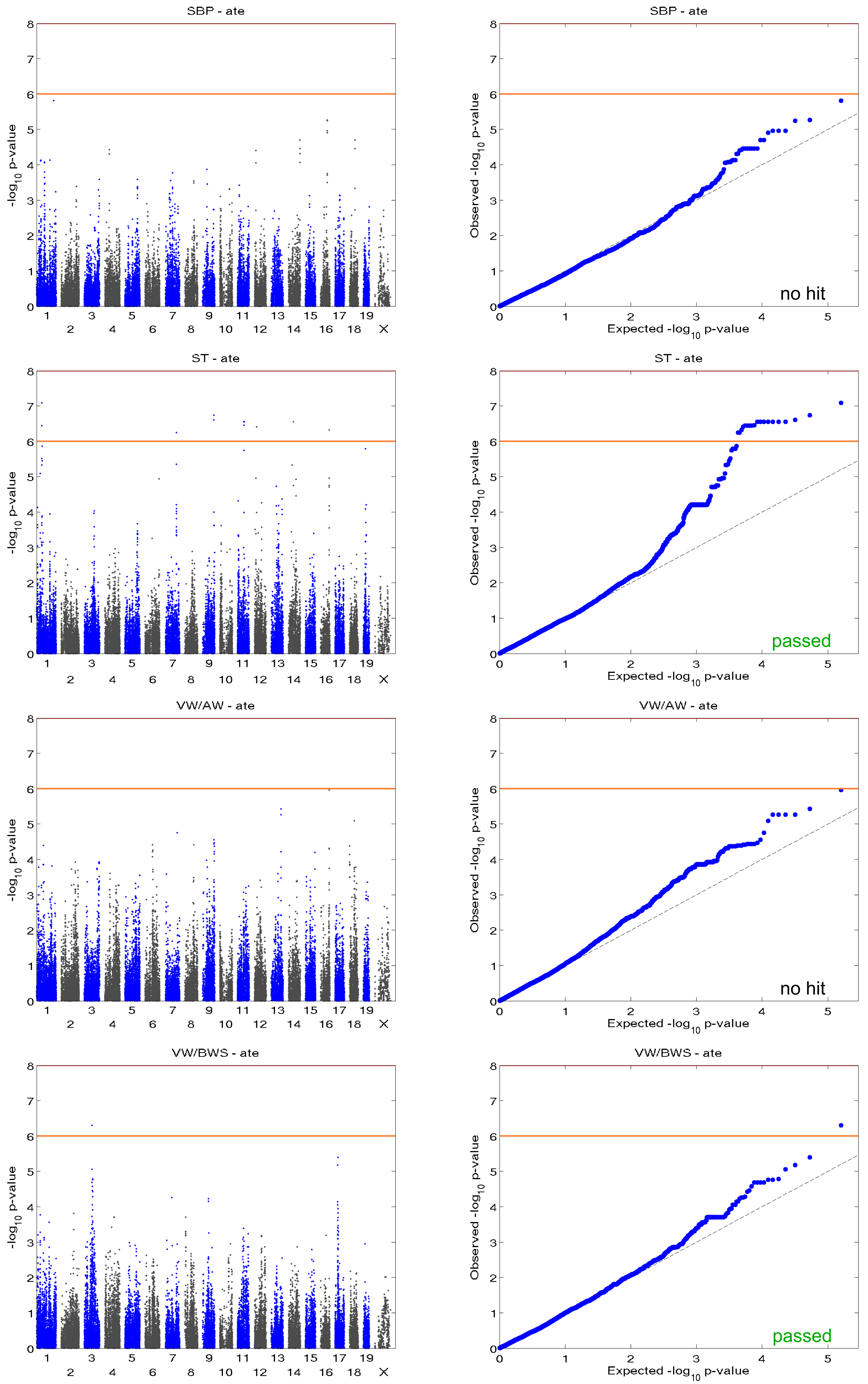
SBP - ate
-log10 p-value
1 3 5 7 9 11 13 15 17 19
2 4 6 8 10 12 14 16 18 X
SBP - ate
Observed -log10 p-value
Expected -log10 p-value
no hit
ST - ate
-log10 p-value
1 3 5 7 9 11 13 15 17 19
2 4 6 8 10 12 14 16 18 X
ST - ate
Observed -log10 p-value
Expected -log10 p-value
passed
VW/AW - ate
-log10 p-value
1 3 5 7 9 11 13 15 17 19
2 4 6 8 10 12 14 16 18 X
VW/AW - ate
Observed -log10 p-value
Expected -log10 p-value
no hit
VW/BWS - ate
-log10 p-value
1 3 5 7 9 11 13 15 17 19
2 4 6 8 10 12 14 16 18 X
VW/BWS - ate
Observed -log10 p-value
Expected -log10 p-value
passed

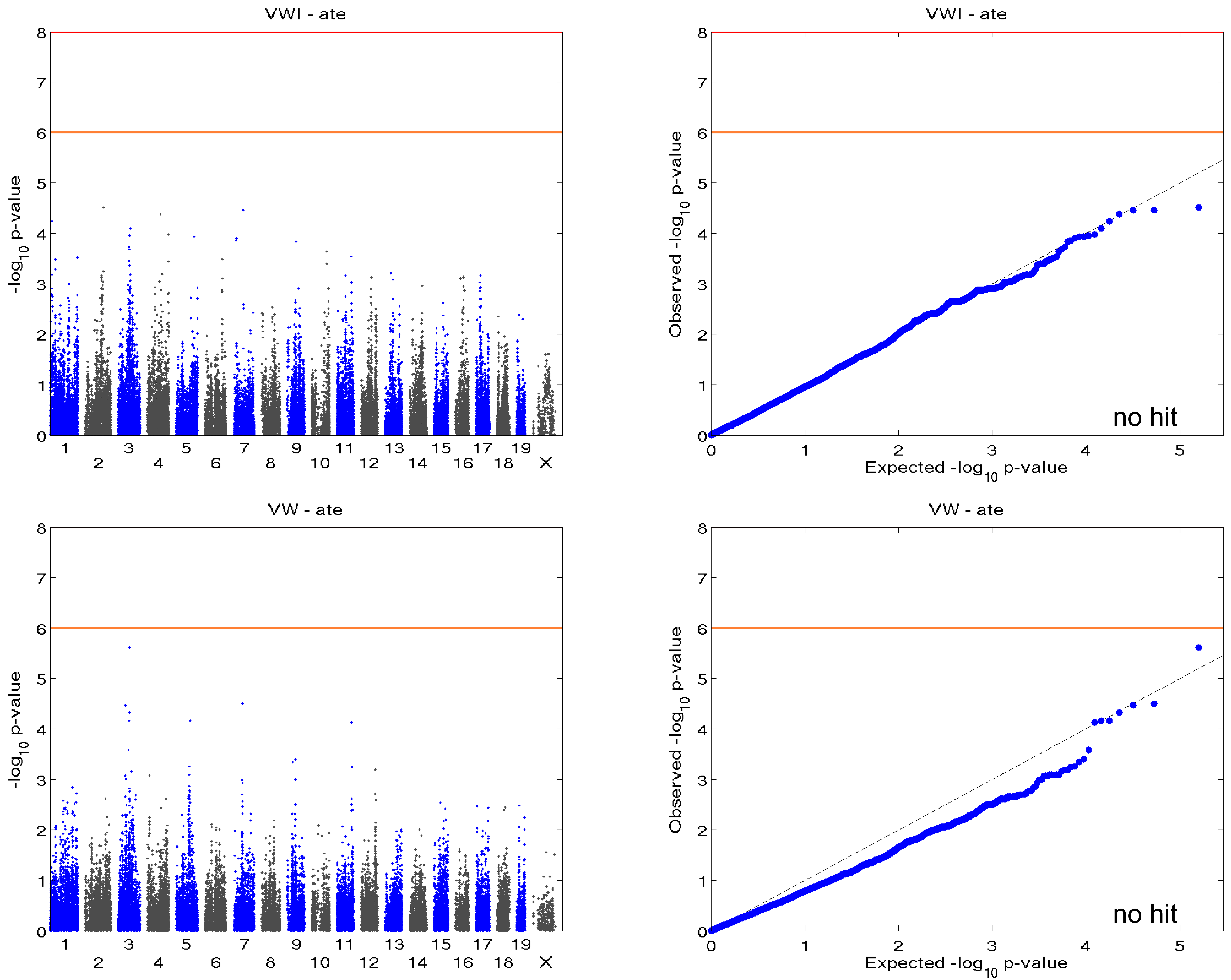
VWI - ate
-log10 p-value
Observed -log10 p-value
Expected -log10 p-value
no hit
VW - ate
-log10 p-value
Observed -log10 p-value
Expected -log10 p-value
no hit
1 2 3 4 5 6 7 8 9 10 11 12 13 14 15 16 17 18 19 X
