# Supplementary material for: Mapping Genetic Variants Associated with Beta-Adrenergic Responses in Inbred Mice
Source: PLoS One. 2012 Jul 31;7(7):e41032. doi: 10.1371/journal.pone.0041032 (PMC3409184; doi:10.1371/journal.pone.0041032)
Supplement: Figure S3 — Manhattan and QQ-plots for 26 traits measured in iso1 -treated mice. QQ-plot-based quality control is indicated as “passed” or “failed”. (PDF) [file pone.0041032.s003.pdf]

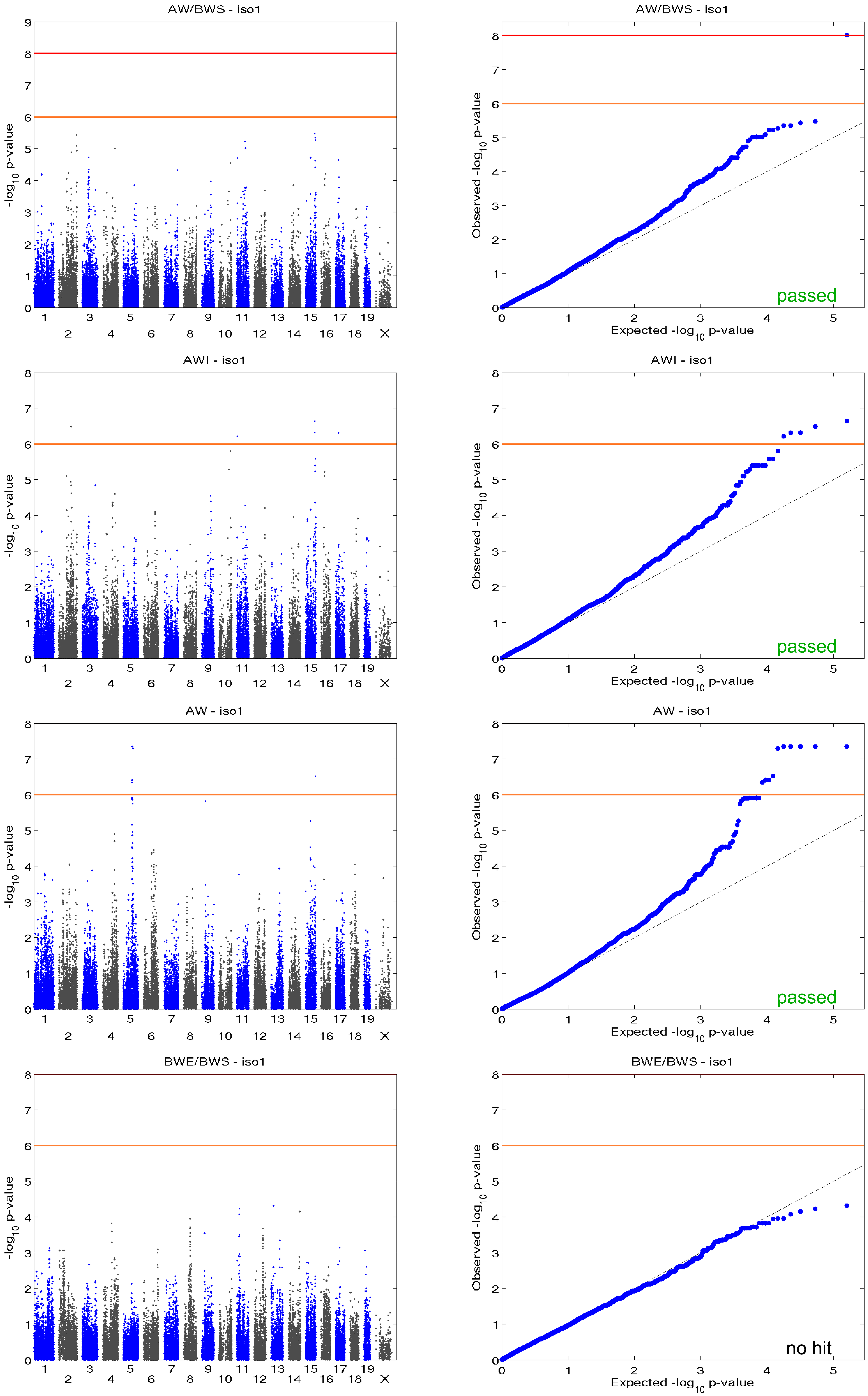
AW/BWS - iso1
-log10 p-value
AW/BWS - iso1
Observed -log10 p-value
Expected -log10 p-value
passed
AWI - iso1
-log10 p-value
AWI - iso1
Observed -log10 p-value
Expected -log10 p-value
passed
AW - iso1
-log10 p-value
AW - iso1
Observed -log10 p-value
Expected -log10 p-value
passed
BWE/BWS - iso1
-log10 p-value
BWE/BWS - iso1
Observed -log10 p-value
Expected -log10 p-value
no hit

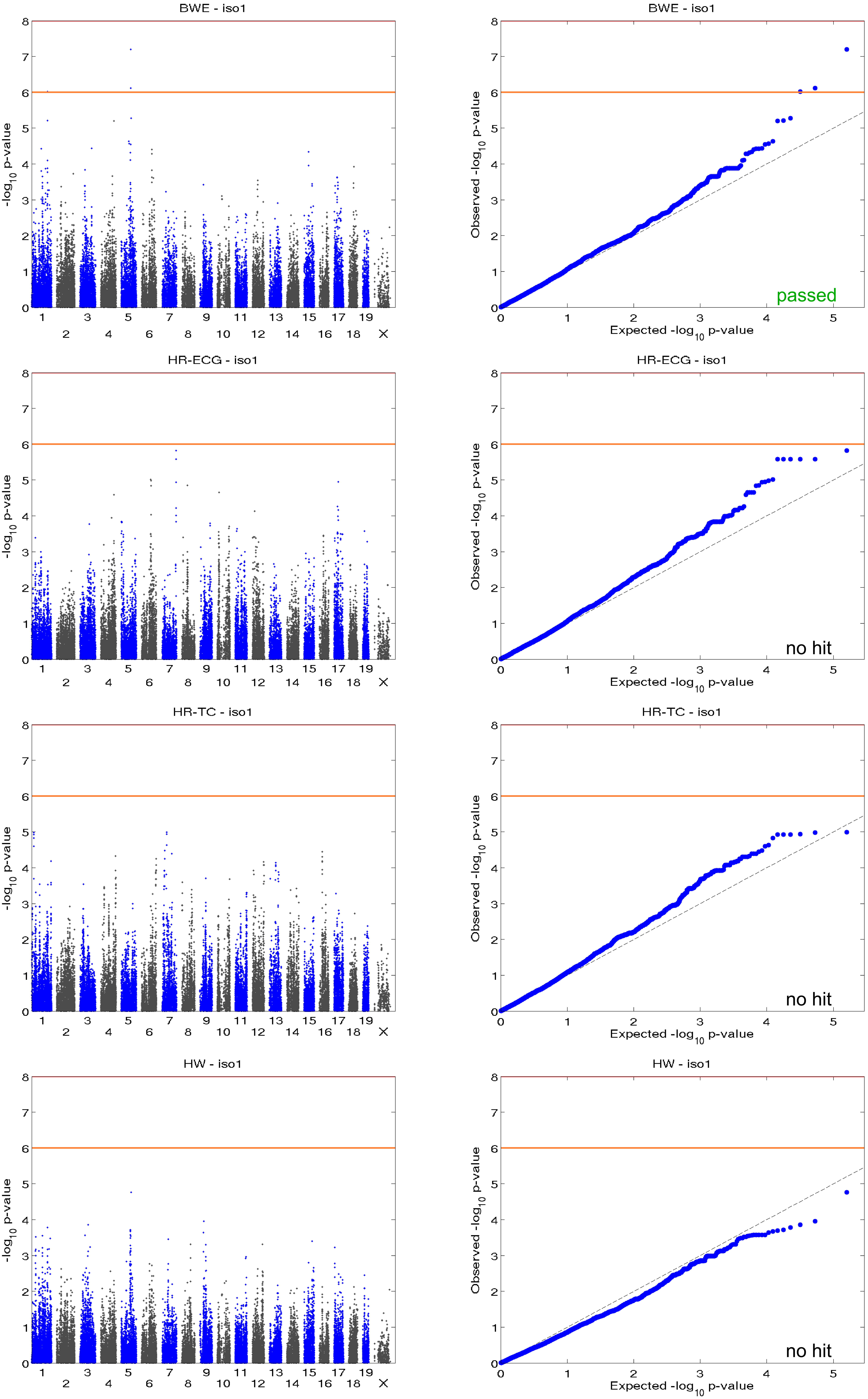

BWE - iso1
BWE - iso1
passed
HR-ECG - iso1
HR-ECG - iso1
no hit
HR-TC - iso1
HR-TC - iso1
no hit
HW - iso1
HW - iso1
no hit
$-\log_{10}$ p-value
Observed $-\log_{10}$ p-value
Expected $-\log_{10}$ p-value
1 2 3 4 5 6 7 8 9 10 11 12 13 14 15 16 17 18 19 X

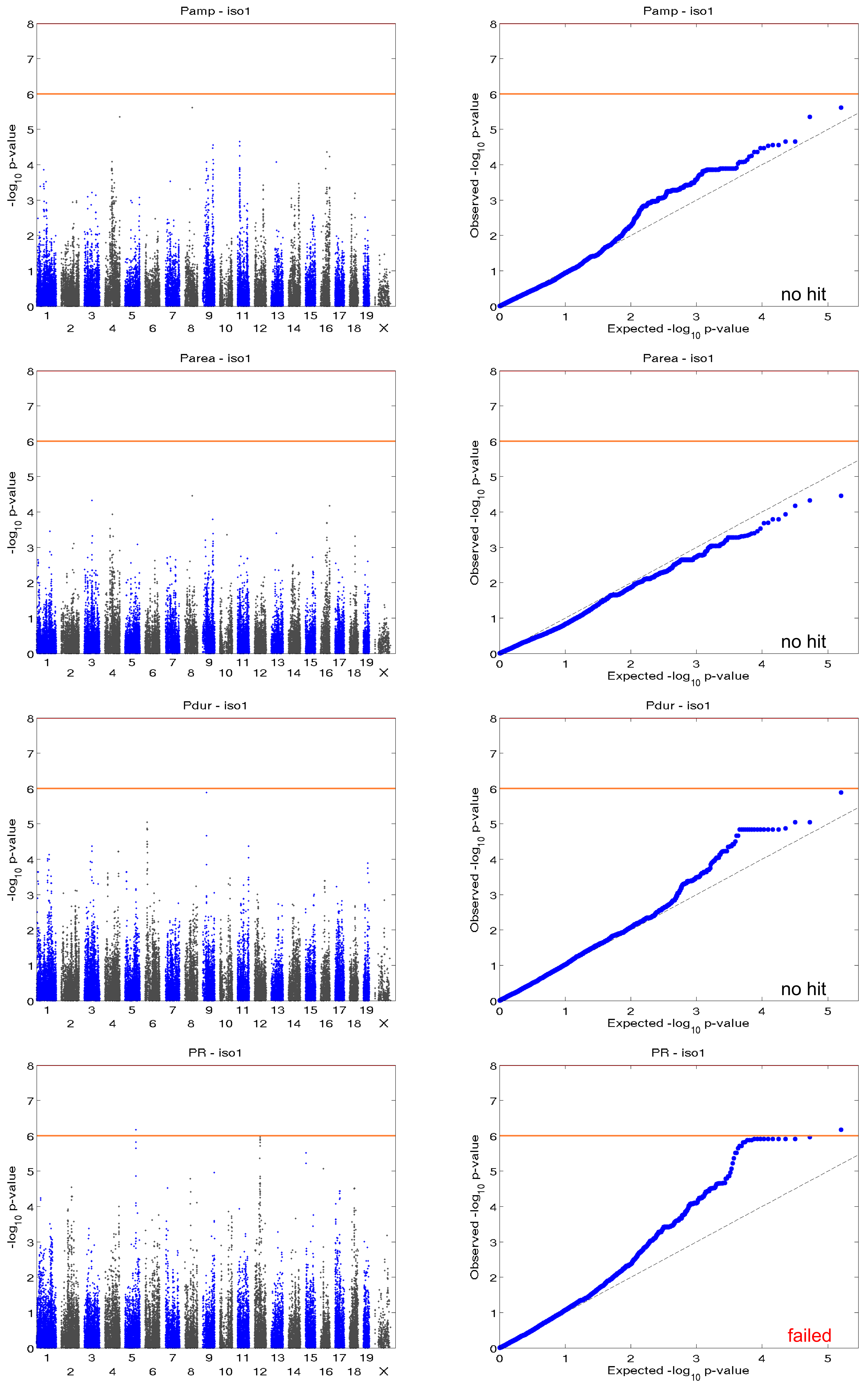
Pamp - iso1
-log10 p-value
Pamp - iso1
Observed -log10 p-value
Expected -log10 p-value
no hit
Parea - iso1
-log10 p-value
Parea - iso1
Observed -log10 p-value
Expected -log10 p-value
no hit
Pdur - iso1
-log10 p-value
Pdur - iso1
Observed -log10 p-value
Expected -log10 p-value
no hit
PR - iso1
-log10 p-value
PR - iso1
Observed -log10 p-value
Expected -log10 p-value
failed

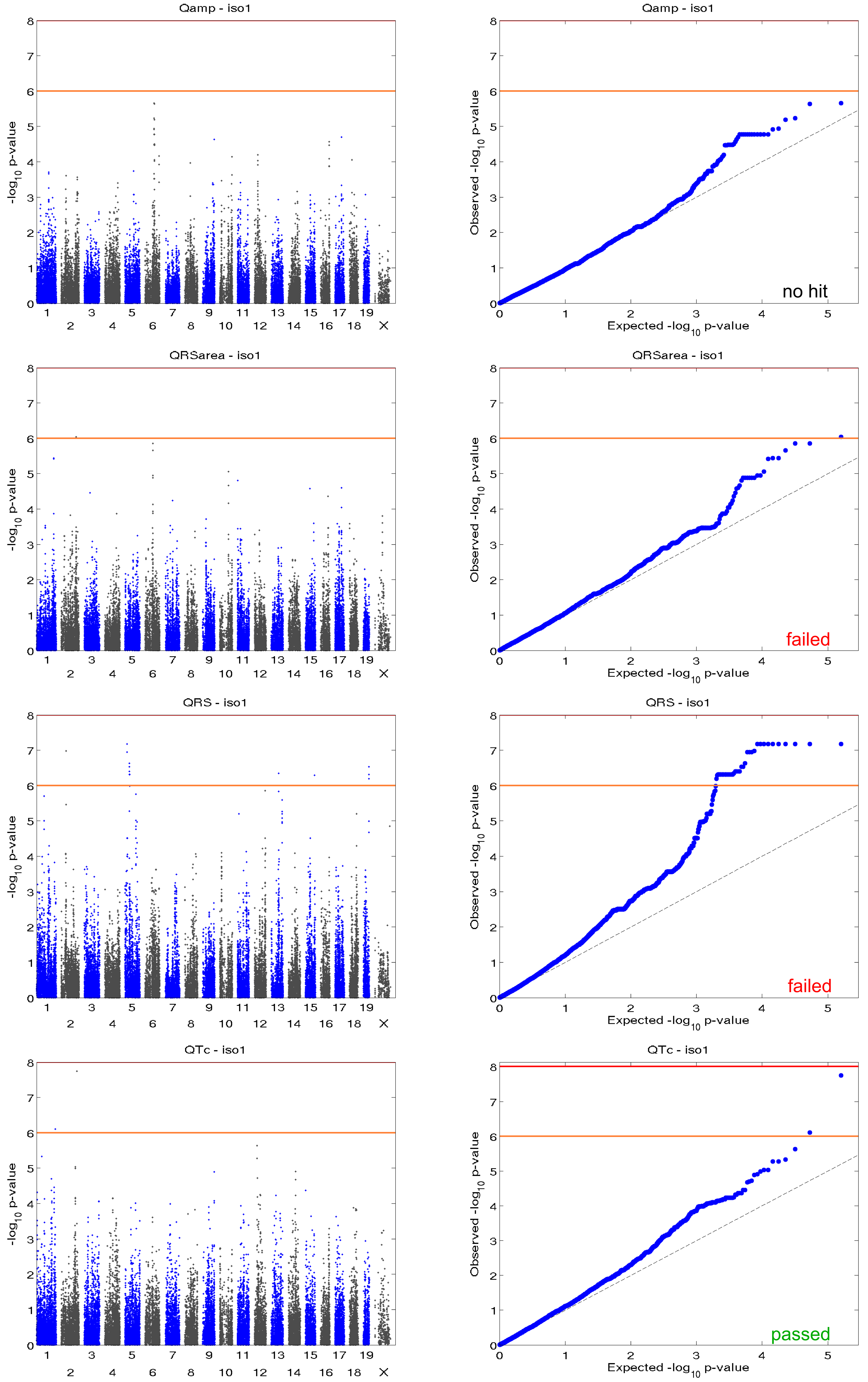
Qamp - iso1
-log10 p-value
Qamp - iso1
Observed -log10 p-value
Expected -log10 p-value
no hit
QRSarea - iso1
-log10 p-value
QRSarea - iso1
Observed -log10 p-value
Expected -log10 p-value
failed
QRS - iso1
-log10 p-value
QRS - iso1
Observed -log10 p-value
Expected -log10 p-value
failed
QTc - iso1
-log10 p-value
QTc - iso1
Observed -log10 p-value
Expected -log10 p-value
passed

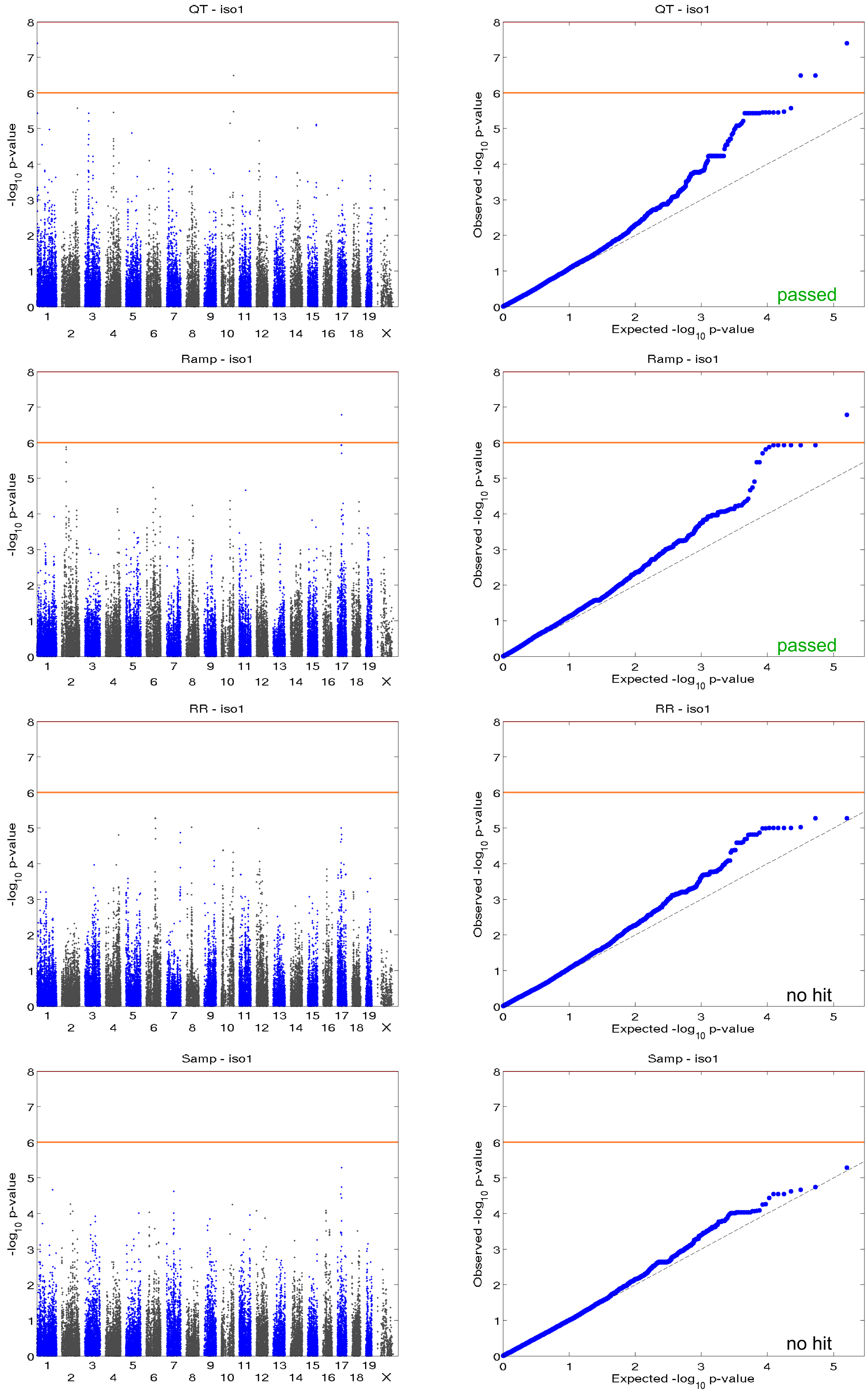
QT - iso1
-log10 p-value
Observed -log10 p-value
Expected -log10 p-value
passed
Ramp - iso1
-log10 p-value
Observed -log10 p-value
Expected -log10 p-value
passed
RR - iso1
-log10 p-value
Observed -log10 p-value
Expected -log10 p-value
no hit
Samp - iso1
-log10 p-value
Observed -log10 p-value
Expected -log10 p-value
no hit
1
2
3
4
5
6
7
8
9
10
11
12
13
14
15
16
17
18
19
X

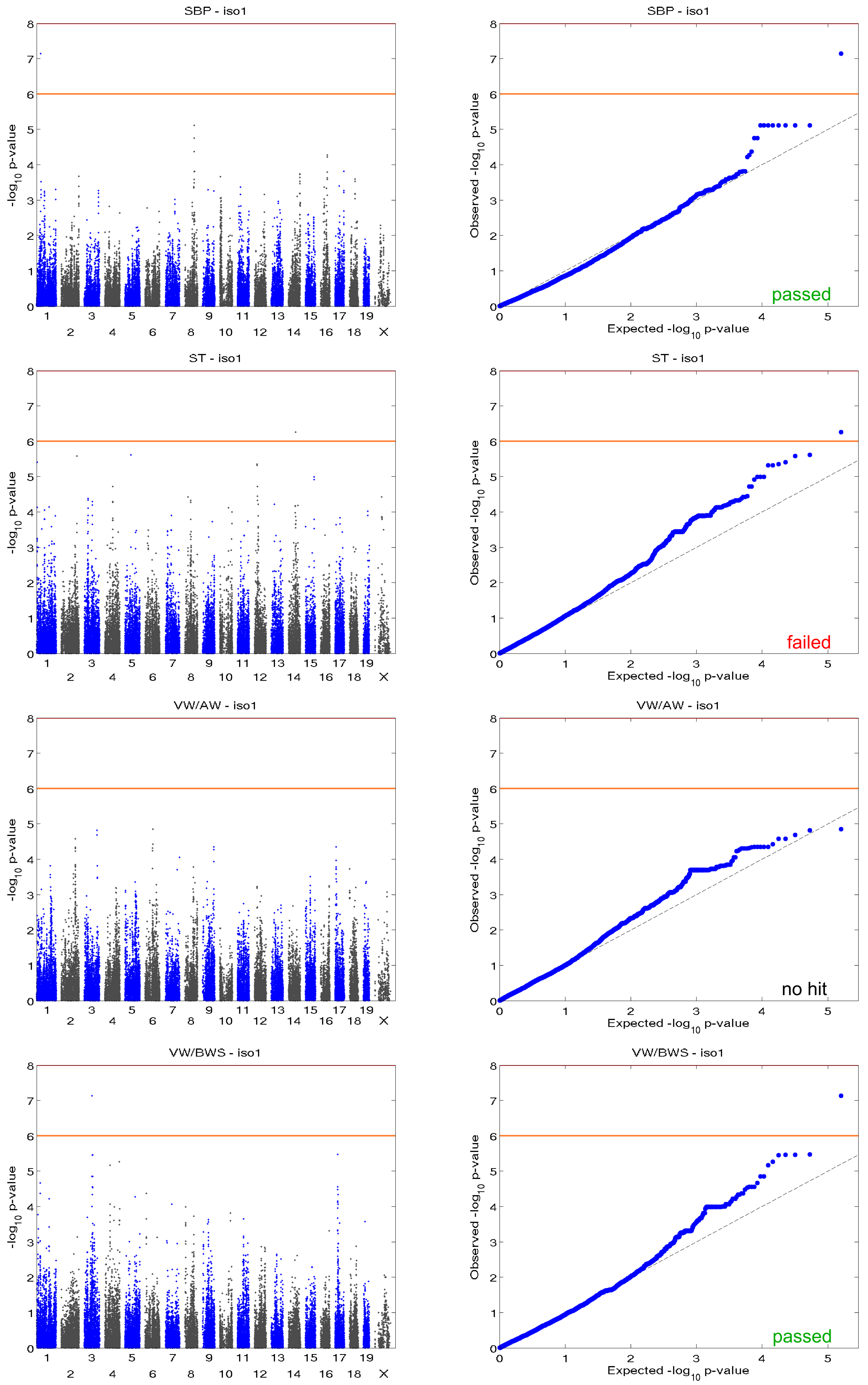
SBP - iso1
SBP - iso1
passed
ST - iso1
ST - iso1
failed
VW/AW - iso1
VW/AW - iso1
no hit
VW/BWS - iso1
VW/BWS - iso1
passed
-log10 p-value
Observed -log10 p-value
Expected -log10 p-value

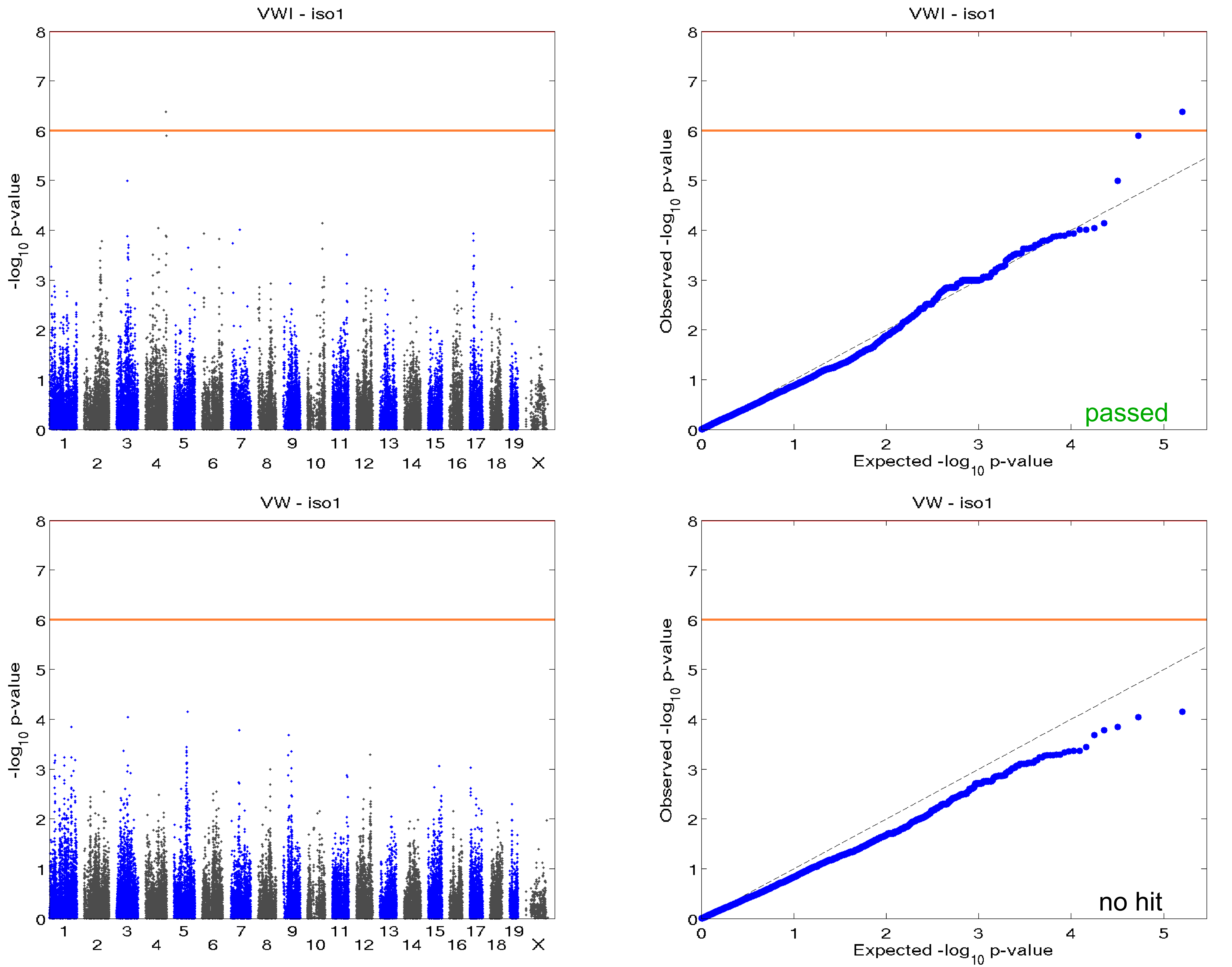

VWI - iso1
-log10 p-value
1 2 3 4 5 6 7 8 9 10 11 12 13 14 15 16 17 18 19 X
VWI - iso1
Observed -log10 p-value
Expected -log10 p-value
passed
VW - iso1
-log10 p-value
1 2 3 4 5 6 7 8 9 10 11 12 13 14 15 16 17 18 19 X
VW - iso1
Observed -log10 p-value
Expected -log10 p-value
no hit
