# Supplementary material for: Mapping Genetic Variants Associated with Beta-Adrenergic Responses in Inbred Mice
Source: PLoS One. 2012 Jul 31;7(7):e41032. doi: 10.1371/journal.pone.0041032 (PMC3409184; doi:10.1371/journal.pone.0041032)
Supplement: Figure S4 — Manhattan and QQ-plots for 26 traits measured in iso10 -treated mice. QQ-plot-based quality control is indicated as “passed” or “failed”. (PDF) [file pone.0041032.s004.pdf]

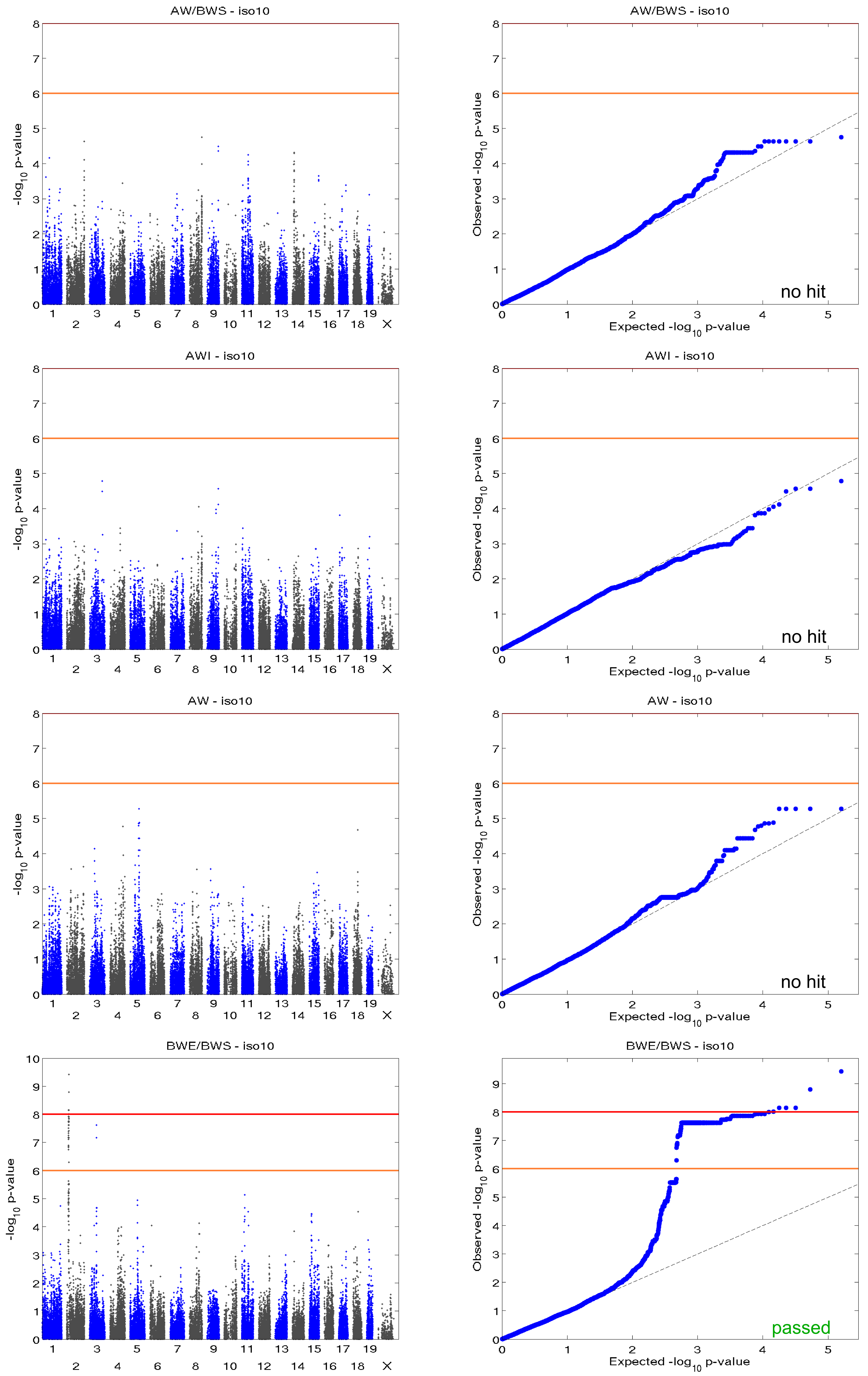

AW/BWS - iso10
-log10 p-value
AW/BWS - iso10
Observed -log10 p-value
Expected -log10 p-value
no hit
AWI - iso10
-log10 p-value
AWI - iso10
Observed -log10 p-value
Expected -log10 p-value
no hit
AW - iso10
-log10 p-value
AW - iso10
Observed -log10 p-value
Expected -log10 p-value
no hit
BWE/BWS - iso10
-log10 p-value
BWE/BWS - iso10
Observed -log10 p-value
Expected -log10 p-value
passed
1 2 3 4 5 6 7 8 9 10 11 12 13 14 15 16 17 18 19 X

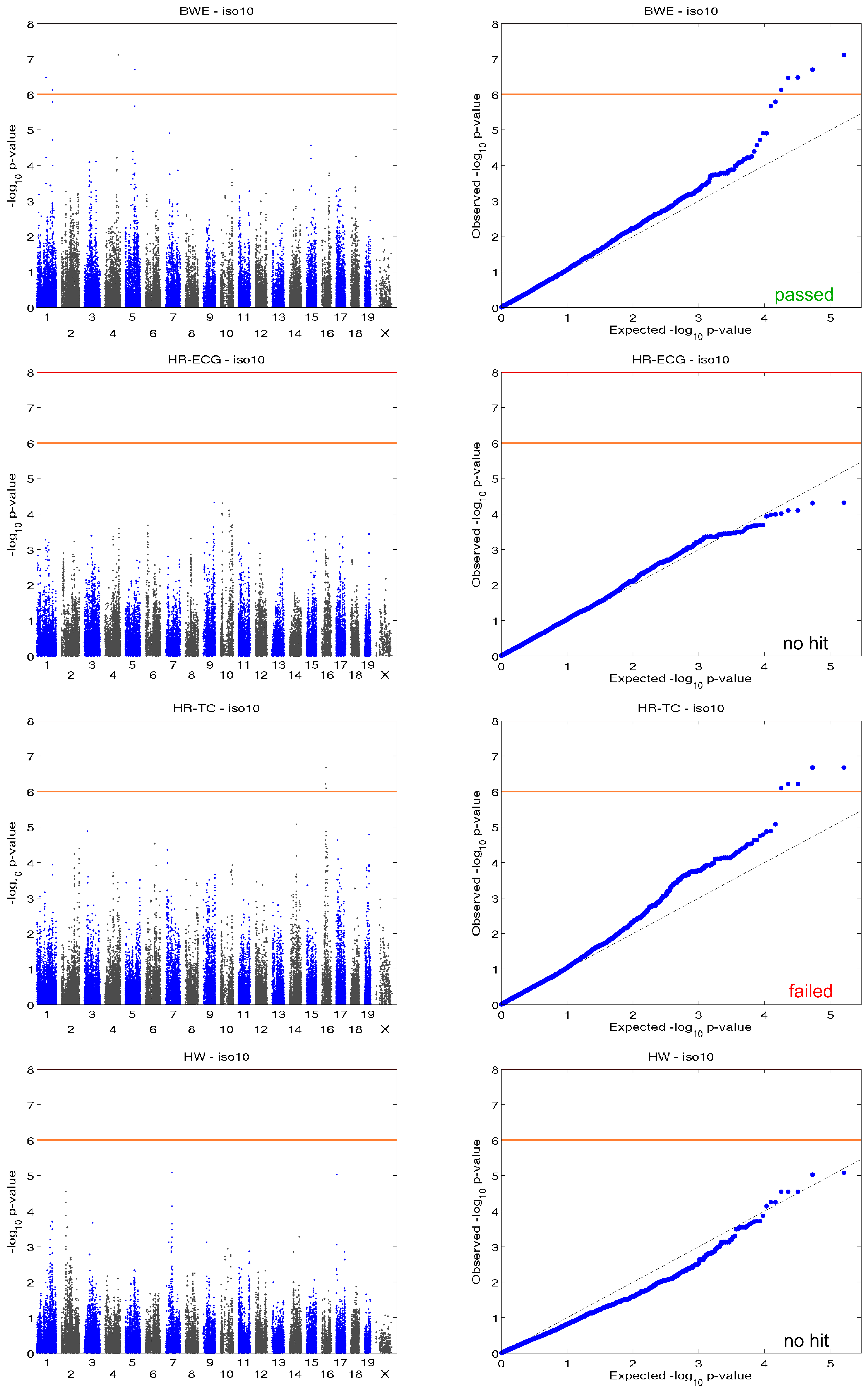
BWE - iso10
HR-ECG - iso10
HR-TC - iso10
HW - iso10
-log10 p-value
Observed -log10 p-value
Expected -log10 p-value
1 2 3 4 5 6 7 8 9 10 11 12 13 14 15 16 17 18 19 X
passed
no hit
failed
no hit

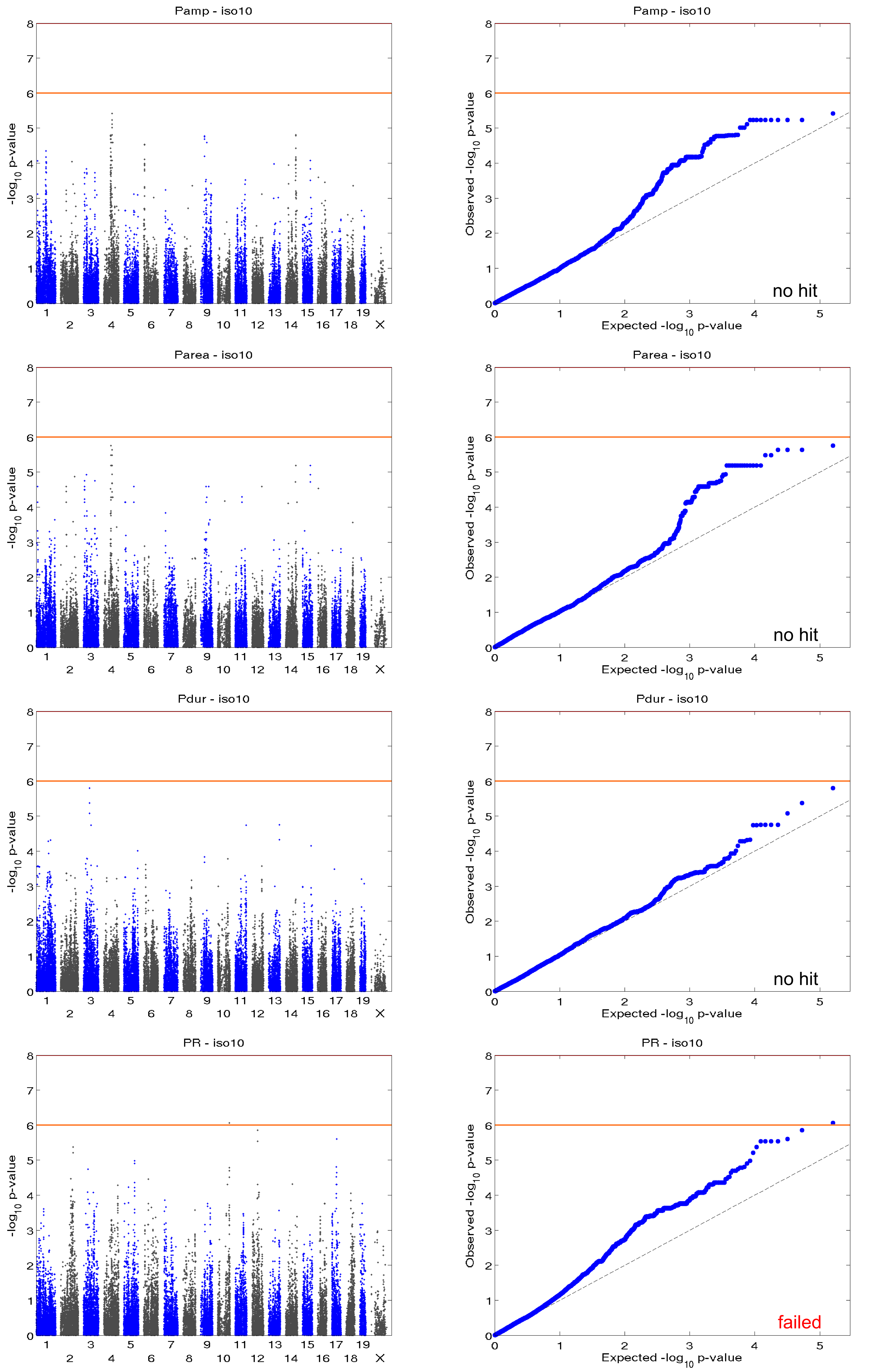

Pamp - iso10
Parea - iso10
Pdur - iso10
PR - iso10
-log10 p-value
Observed -log10 p-value
Expected -log10 p-value
1 2 3 4 5 6 7 8 9 10 11 12 13 14 15 16 17 18 19 X
no hit
no hit
no hit
failed

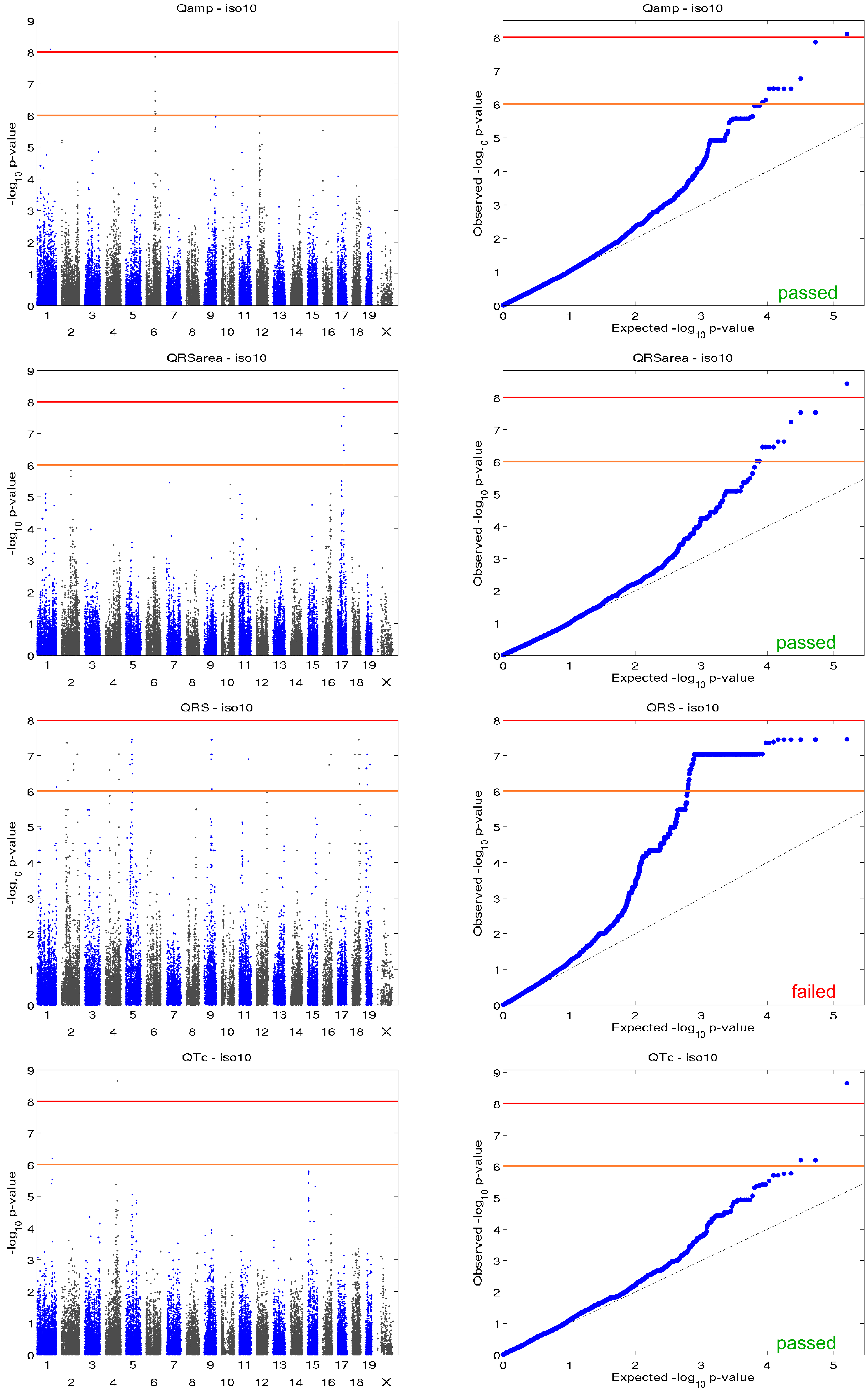

Qamp - iso10
-log10 p-value
Qamp - iso10
Observed -log10 p-value
Expected -log10 p-value
passed
QRSarea - iso10
-log10 p-value
QRSarea - iso10
Observed -log10 p-value
Expected -log10 p-value
passed
QRS - iso10
-log10 p-value
QRS - iso10
Observed -log10 p-value
Expected -log10 p-value
failed
QTc - iso10
-log10 p-value
QTc - iso10
Observed -log10 p-value
Expected -log10 p-value
passed
1 2 3 4 5 6 7 8 9 10 11 12 13 14 15 16 17 18 19 X

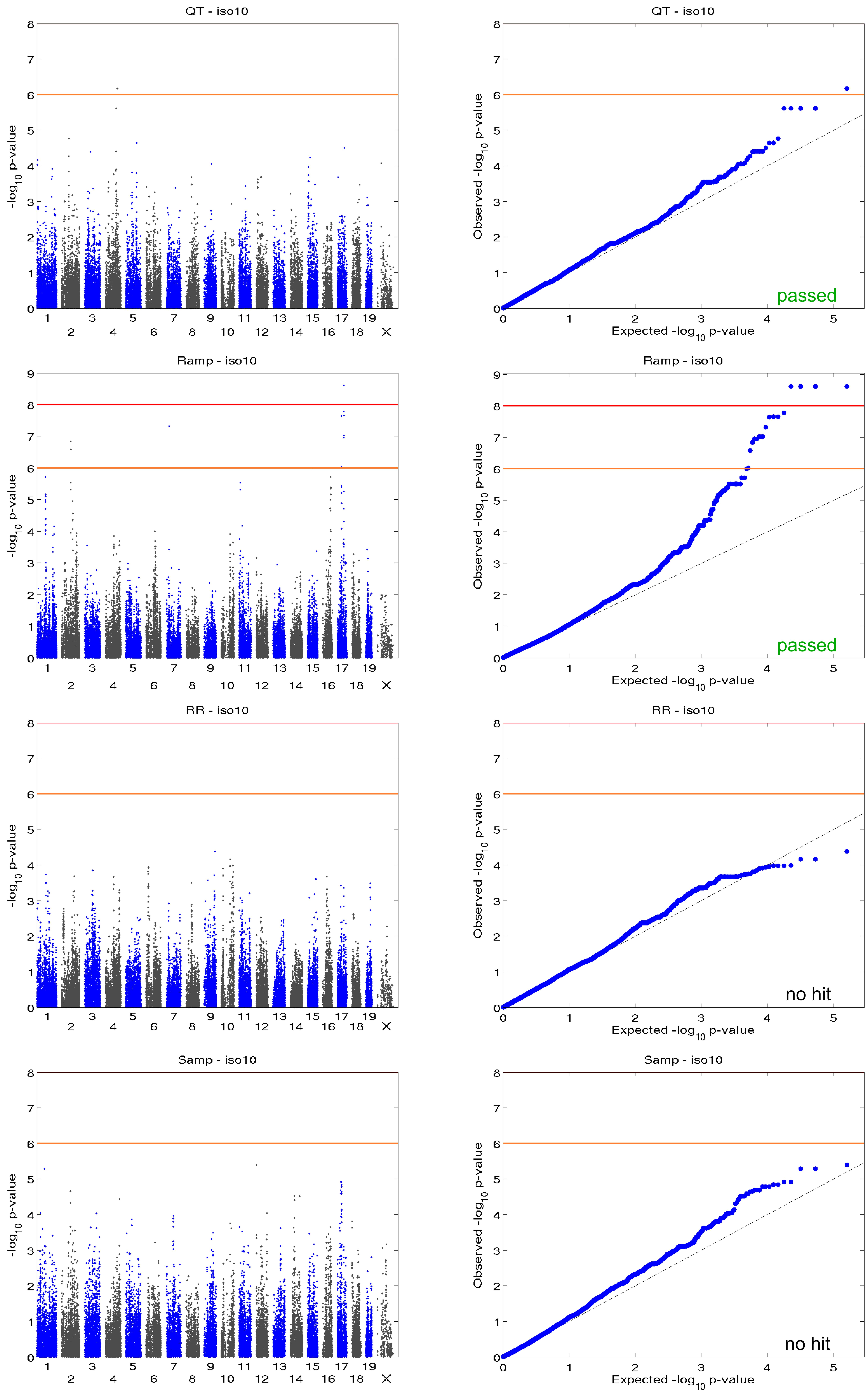
QT - iso10
Ramp - iso10
RR - iso10
Samp - iso10
$-\log_{10}$ p-value
Observed $-\log_{10}$ p-value
Expected $-\log_{10}$ p-value
1 2 3 4 5 6 7 8 9 10 11 12 13 14 15 16 17 18 19 X
passed
passed
no hit
no hit

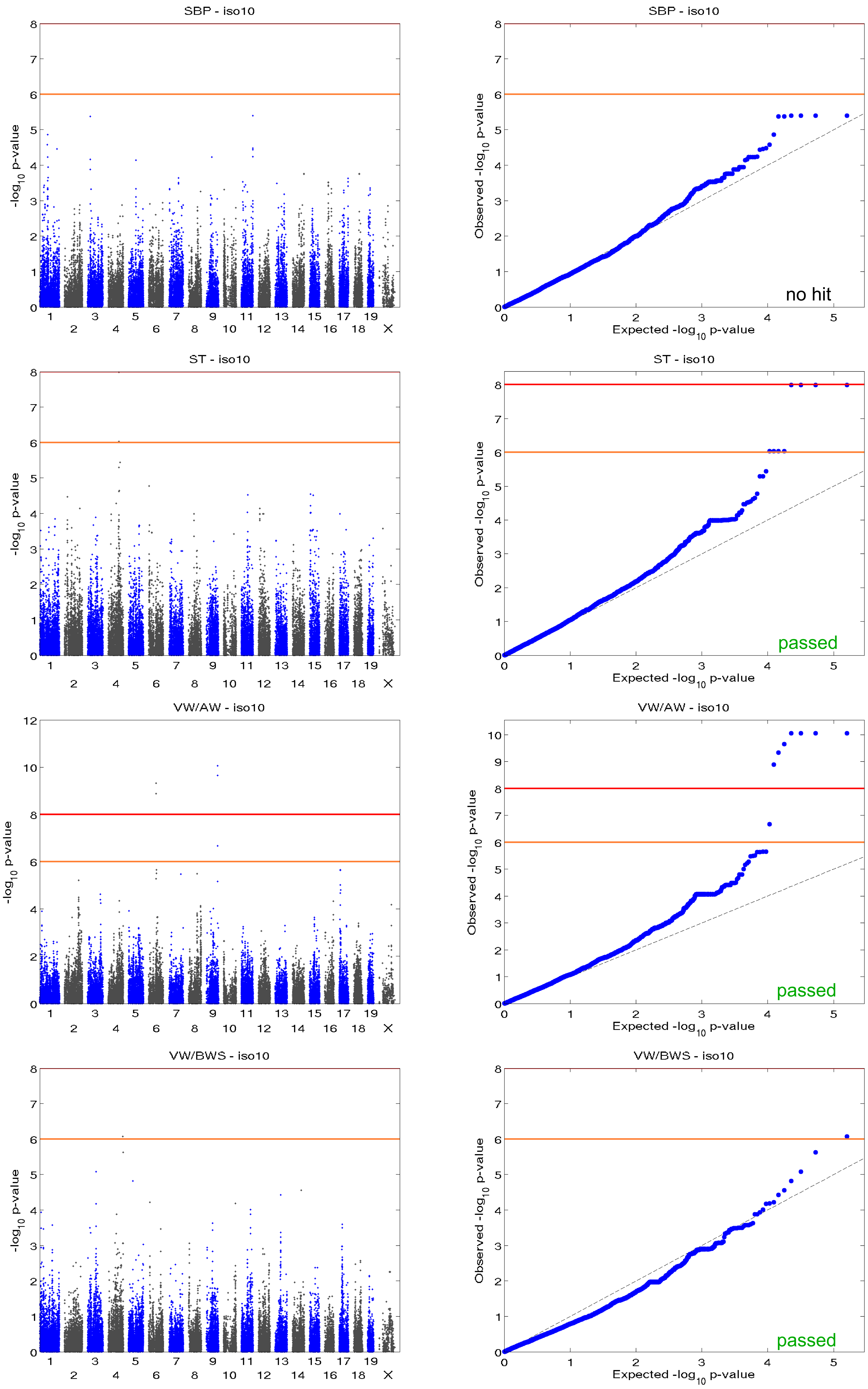
SBP - iso10
-log10 p-value
SBP - iso10
Observed -log10 p-value
Expected -log10 p-value
no hit
ST - iso10
-log10 p-value
ST - iso10
Observed -log10 p-value
Expected -log10 p-value
passed
VW/AW - iso10
-log10 p-value
VW/AW - iso10
Observed -log10 p-value
Expected -log10 p-value
passed
VW/BWS - iso10
-log10 p-value
VW/BWS - iso10
Observed -log10 p-value
Expected -log10 p-value
passed

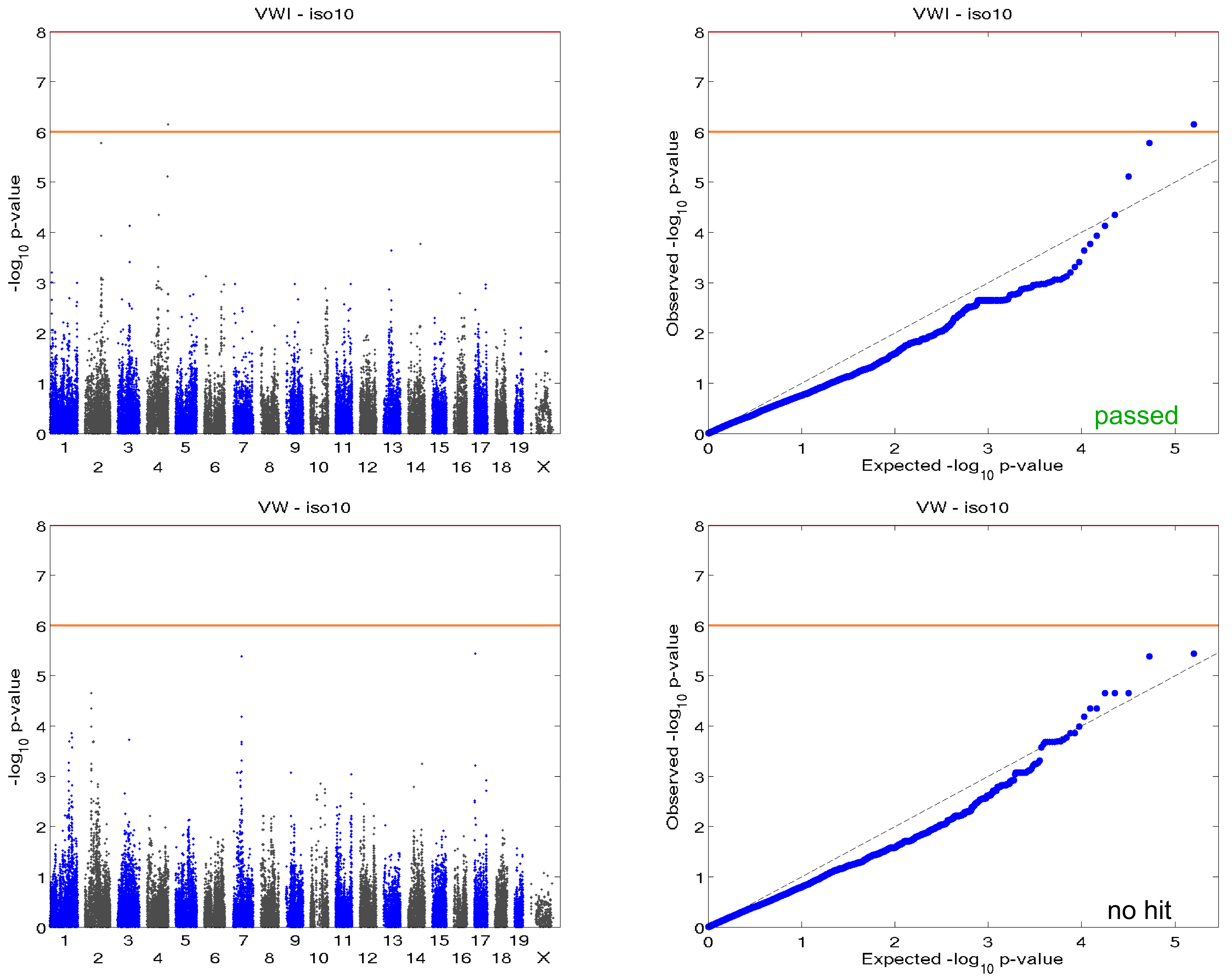

VWI - iso10
-log10 p-value
Observed -log10 p-value
Expected -log10 p-value
passed
VW - iso10
no hit
1 2 3 4 5 6 7 8 9 10 11 12 13 14 15 16 17 18 19 X
