# Supplementary material for: Mapping Genetic Variants Associated with Beta-Adrenergic Responses in Inbred Mice
Source: PLoS One. 2012 Jul 31;7(7):e41032. doi: 10.1371/journal.pone.0041032 (PMC3409184; doi:10.1371/journal.pone.0041032)
Supplement: Figure S5 — Manhattan and QQ-plots for the effects of iso10 treatment on 25 traits. QQ-plot-based quality control is indicated as “passed” or failed”. Phenotypes for which any of the differences between an individual trait value and its matching mean strain value exceeded 3 SD are labelled as “var test failed”. (PDF) [file pone.0041032.s005.pdf]

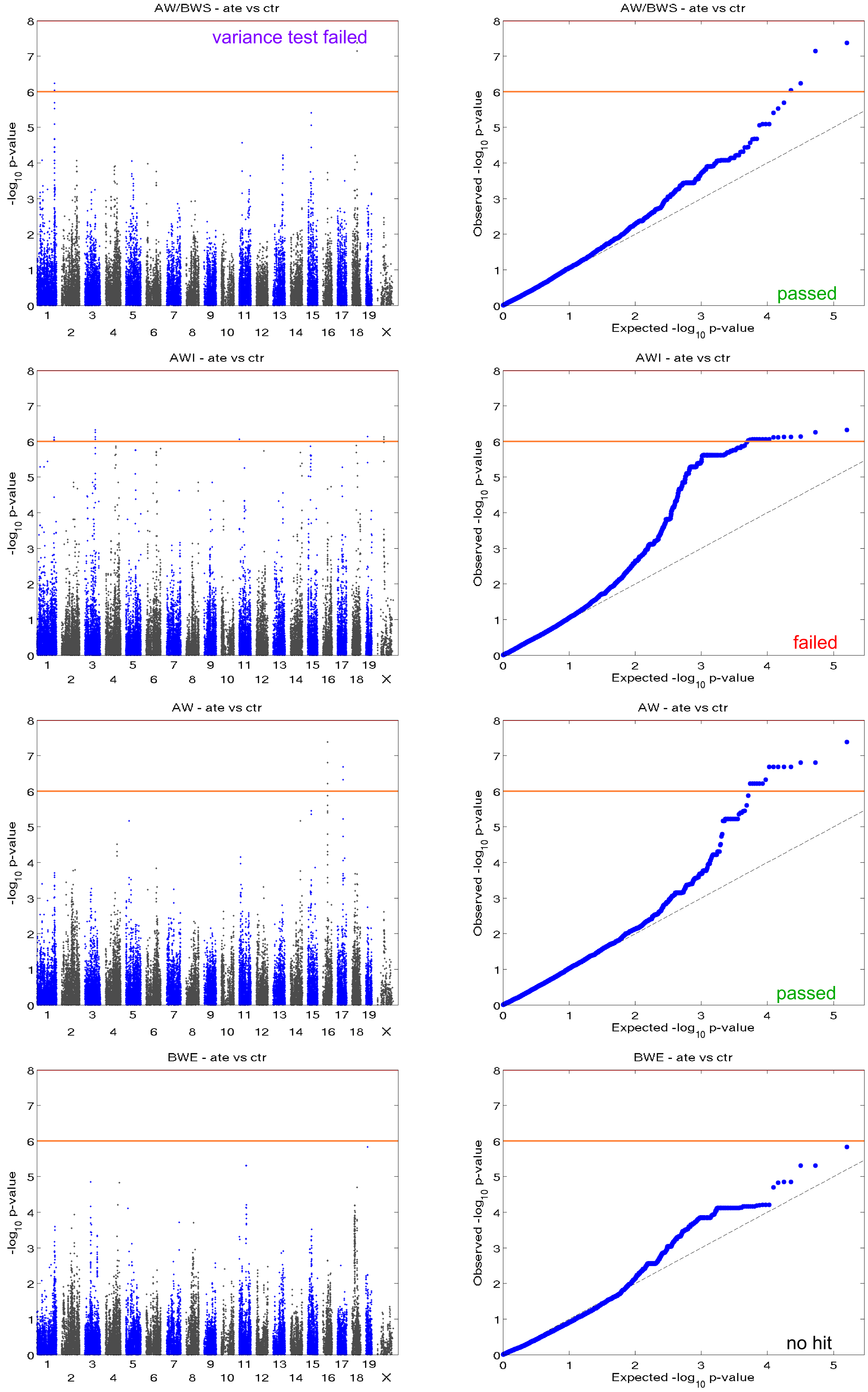

AW/BWS - ate vs ctr
variance test failed
-log10 p-value
AW/BWS - ate vs ctr
Observed -log10 p-value
Expected -log10 p-value
passed
AWI - ate vs ctr
-log10 p-value
AWI - ate vs ctr
Observed -log10 p-value
Expected -log10 p-value
failed
AW - ate vs ctr
-log10 p-value
AW - ate vs ctr
Observed -log10 p-value
Expected -log10 p-value
passed
BWE - ate vs ctr
-log10 p-value
BWE - ate vs ctr
Observed -log10 p-value
Expected -log10 p-value
no hit

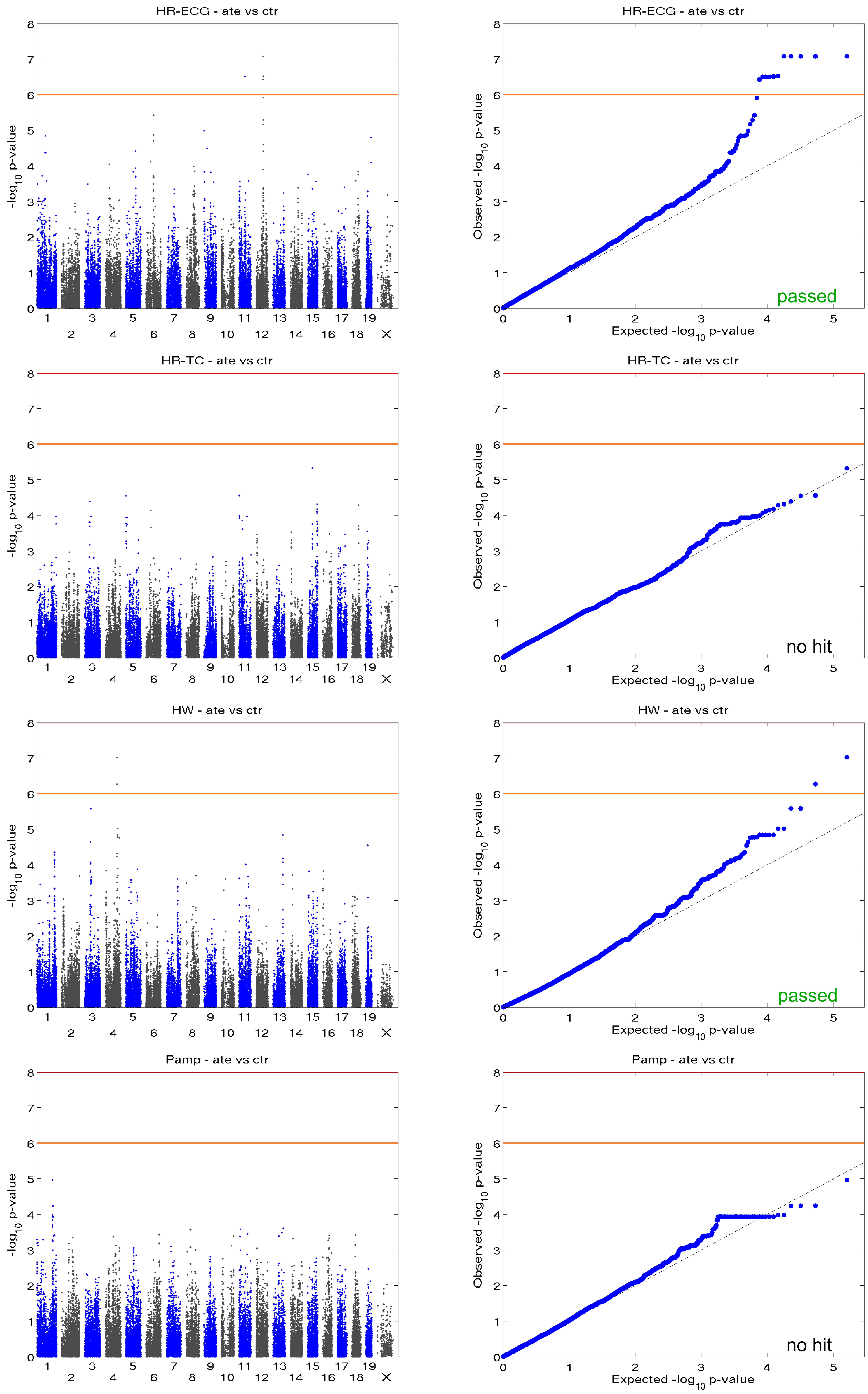

HR-ECG - ate vs ctr
-log10 p-value
HR-ECG - ate vs ctr
Observed -log10 p-value
Expected -log10 p-value
passed
HR-TC - ate vs ctr
-log10 p-value
HR-TC - ate vs ctr
Observed -log10 p-value
Expected -log10 p-value
no hit
HW - ate vs ctr
-log10 p-value
HW - ate vs ctr
Observed -log10 p-value
Expected -log10 p-value
passed
Pamp - ate vs ctr
-log10 p-value
Pamp - ate vs ctr
Observed -log10 p-value
Expected -log10 p-value
no hit

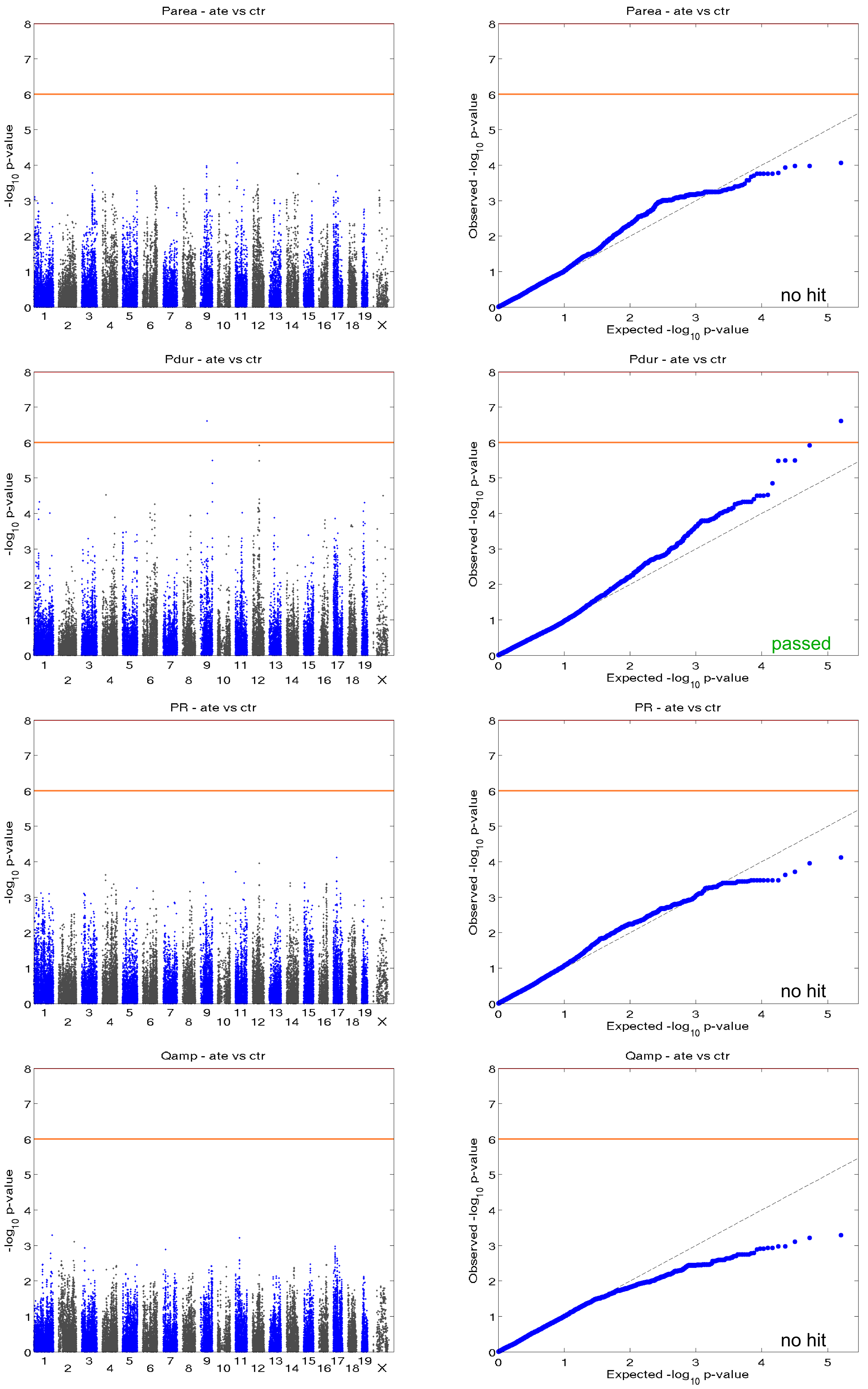
Parea - ate vs ctr
-log10 p-value
Observed -log10 p-value
Expected -log10 p-value
no hit
Pdur - ate vs ctr
passed
PR - ate vs ctr
no hit
Qamp - ate vs ctr
no hit
1 2 3 4 5 6 7 8 9 10 11 12 13 14 15 16 17 18 19 X

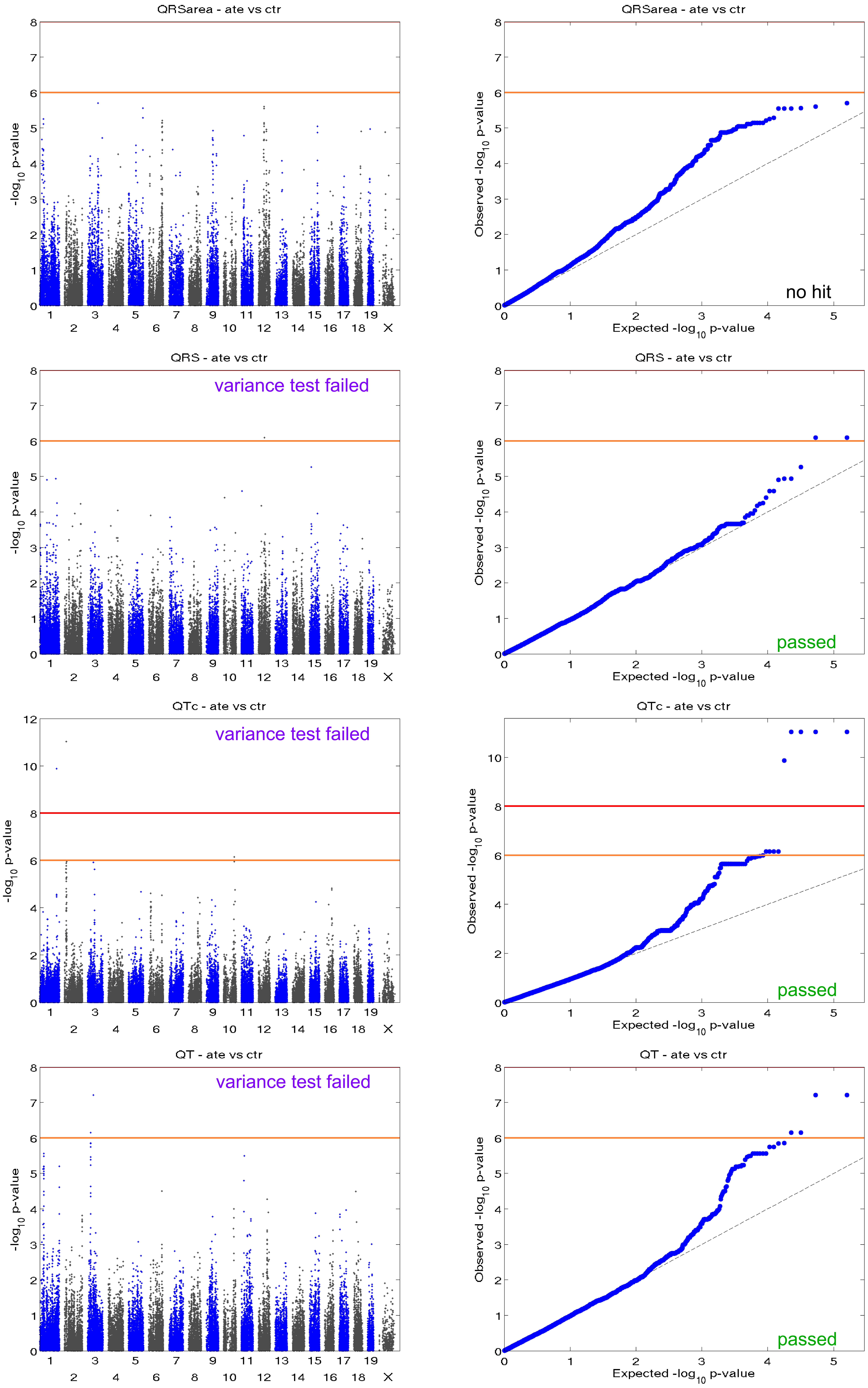

QRSarea - ate vs ctr
-log10 p-value
no hit
Expected -log10 p-value
Observed -log10 p-value
QRS - ate vs ctr
variance test failed
passed
QTc - ate vs ctr
variance test failed
passed
QT - ate vs ctr
variance test failed
passed
1 2 3 4 5 6 7 8 9 10 11 12 13 14 15 16 17 18 19 X

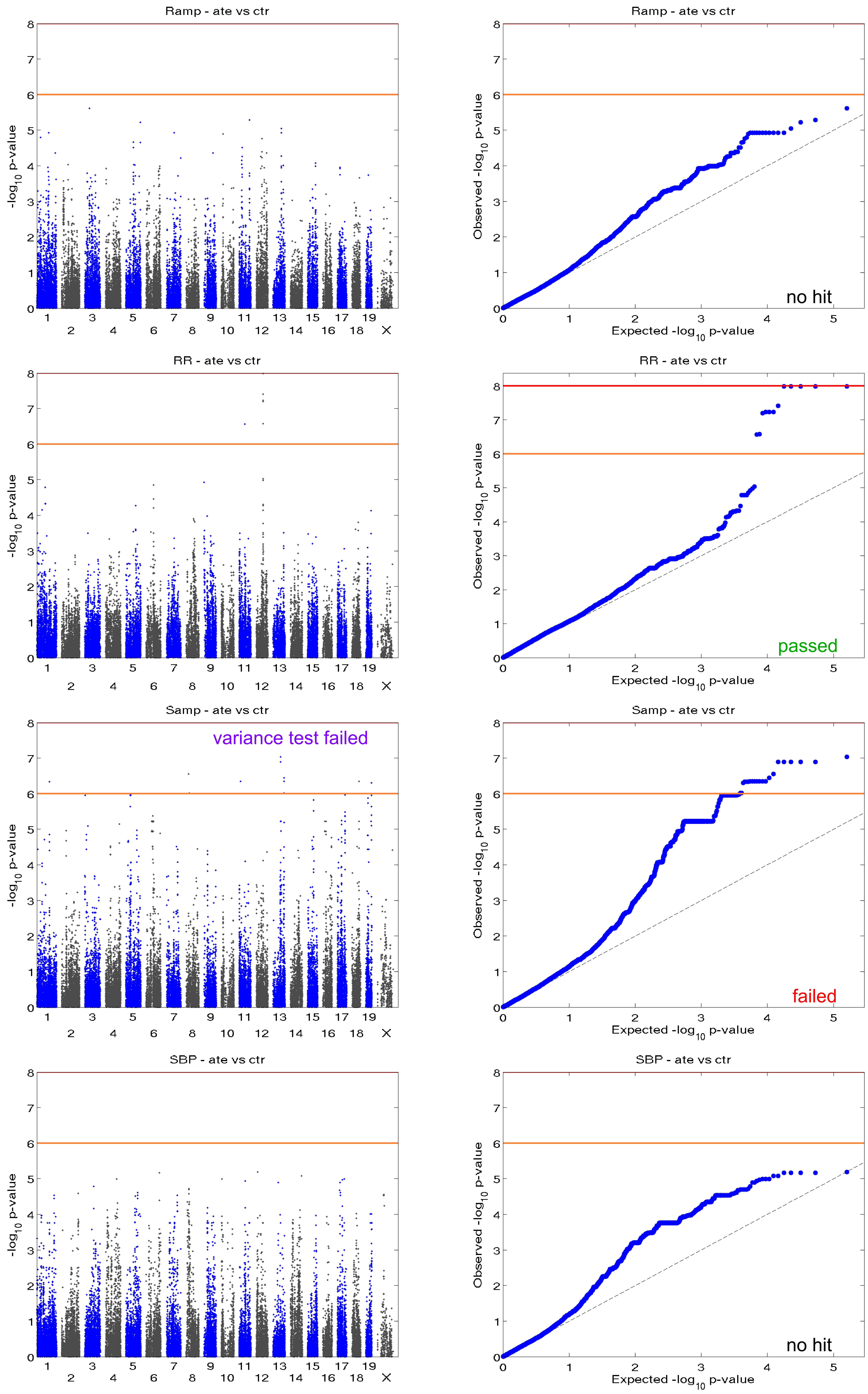

Ramp - ate vs ctr
-log10 p-value
Observed -log10 p-value
Expected -log10 p-value
no hit
RR - ate vs ctr
passed
Samp - ate vs ctr
variance test failed
failed
SBP - ate vs ctr
no hit
1 2 3 4 5 6 7 8 9 10 11 12 13 14 15 16 17 18 19 X

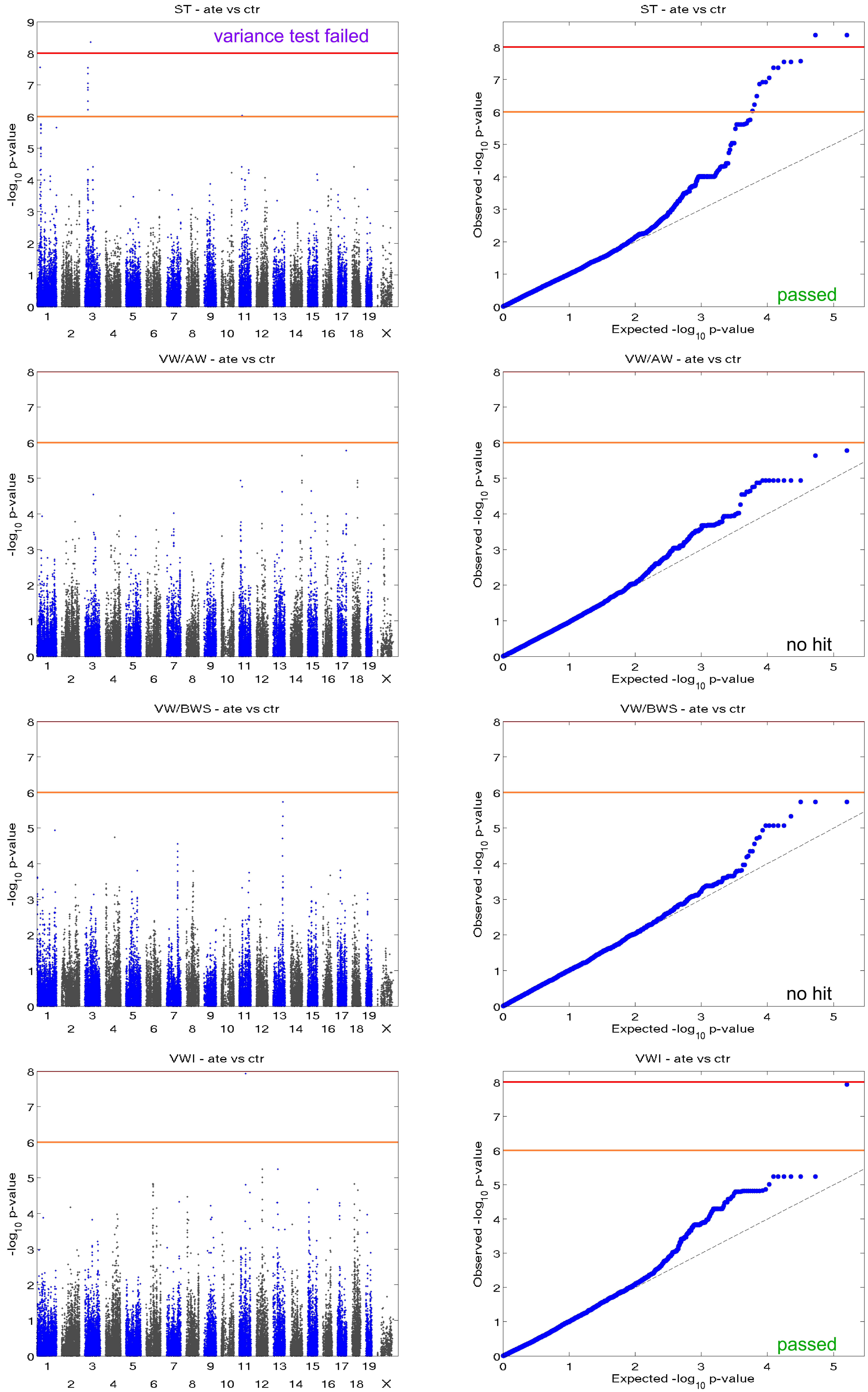
ST - ate vs ctr
variance test failed
-log10 p-value
ST - ate vs ctr
passed
Observed -log10 p-value
Expected -log10 p-value
VW/AW - ate vs ctr
-log10 p-value
VW/AW - ate vs ctr
no hit
Observed -log10 p-value
Expected -log10 p-value
VW/BWS - ate vs ctr
-log10 p-value
VW/BWS - ate vs ctr
no hit
Observed -log10 p-value
Expected -log10 p-value
VWI - ate vs ctr
-log10 p-value
VWI - ate vs ctr
passed
Observed -log10 p-value
Expected -log10 p-value

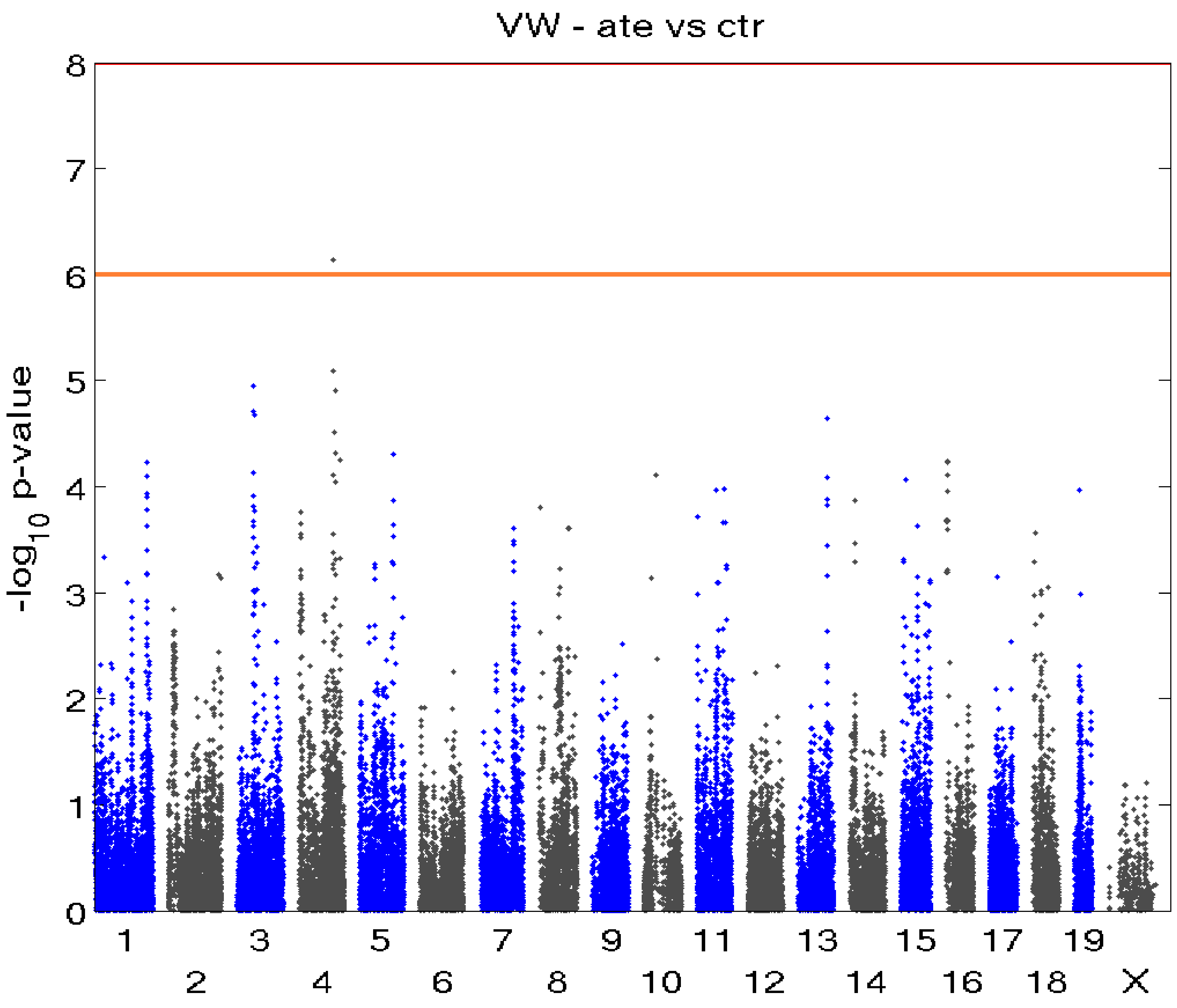
VW - ate vs ctr
-log10 p-value
1
2
3
4
5
6
7
8
9
10
11
12
13
14
15
16
17
18
19
X

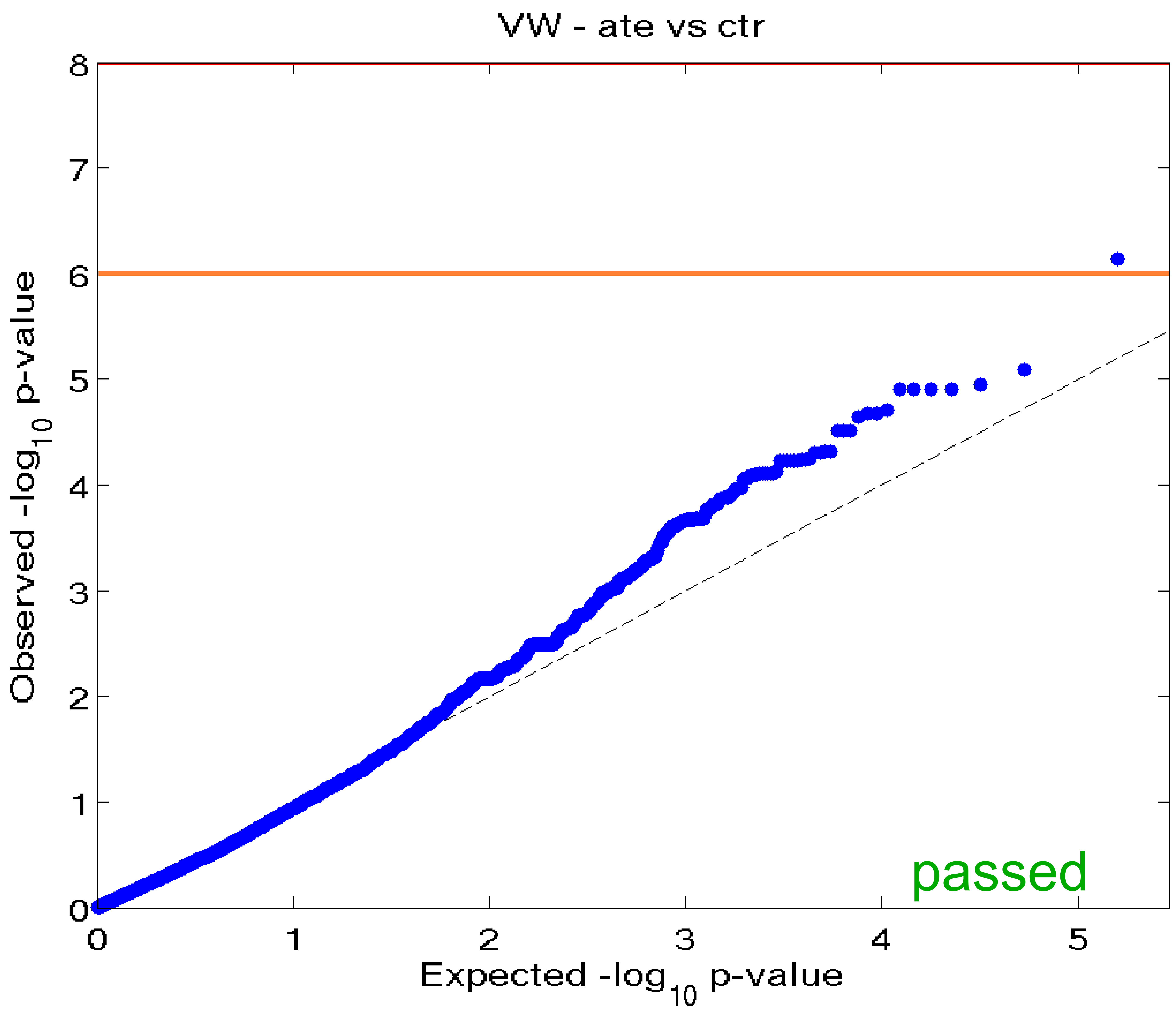
VW - ate vs ctr
Observed -log10 p-value
Expected -log10 p-value
passed
