# Supplementary material for: Mapping Genetic Variants Associated with Beta-Adrenergic Responses in Inbred Mice
Source: PLoS One. 2012 Jul 31;7(7):e41032. doi: 10.1371/journal.pone.0041032 (PMC3409184; doi:10.1371/journal.pone.0041032)
Supplement: Figure S6 — Manhattan and QQ-plots for the effects of ate treatment on 25 traits. QQ-plot-based quality control is indicated as “passed” or failed”. Phenotypes for which any of the differences between an individual trait value and its matching mean strain value exceeded 3 SD are labelled as “var test failed”. (PDF) [file pone.0041032.s006.pdf]

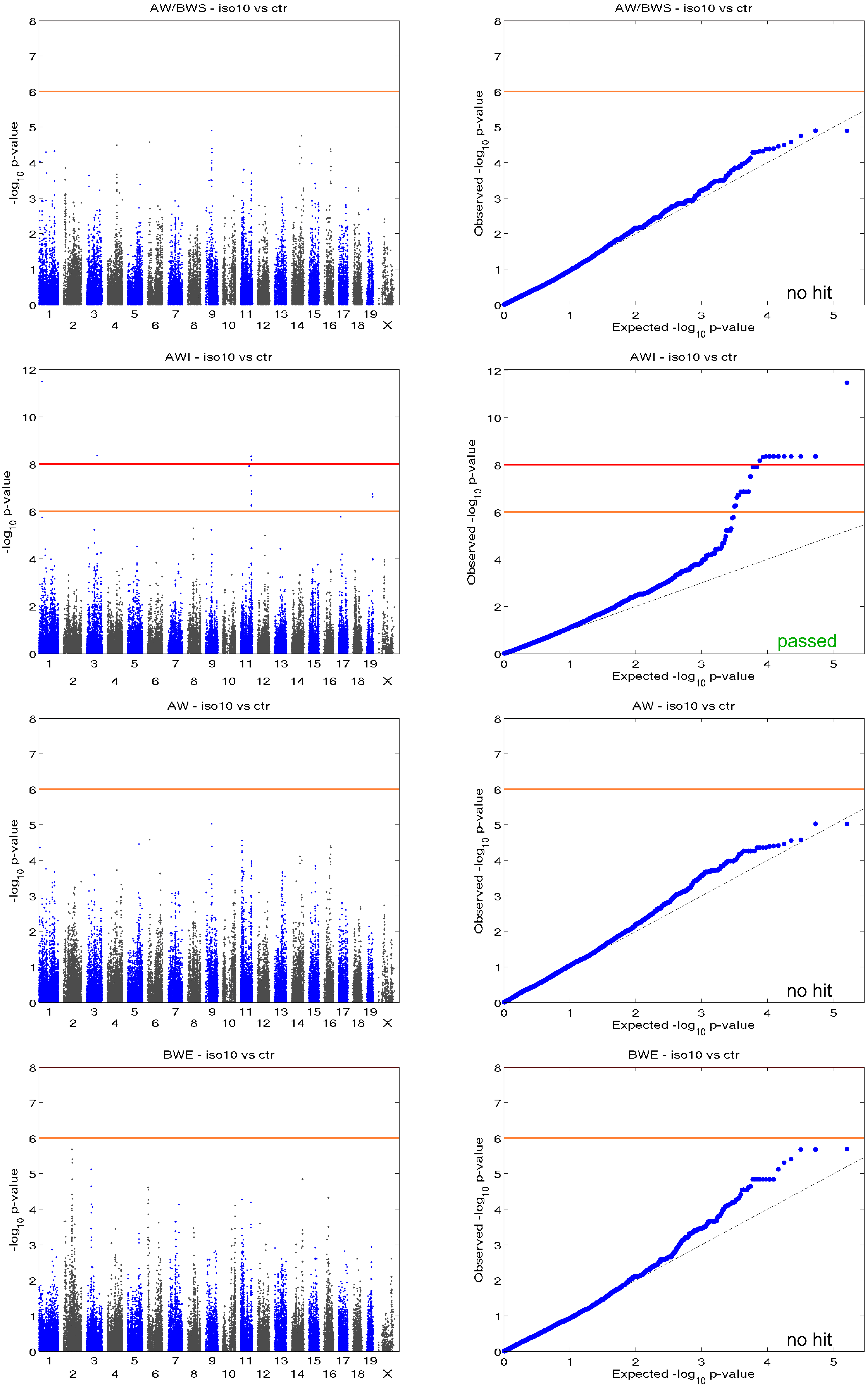

AW/BWS - iso10 vs ctr
-log10 p-value
AW/BWS - iso10 vs ctr
Observed -log10 p-value
Expected -log10 p-value
no hit
AWI - iso10 vs ctr
-log10 p-value
AWI - iso10 vs ctr
Observed -log10 p-value
Expected -log10 p-value
passed
AW - iso10 vs ctr
-log10 p-value
AW - iso10 vs ctr
Observed -log10 p-value
Expected -log10 p-value
no hit
BWE - iso10 vs ctr
-log10 p-value
BWE - iso10 vs ctr
Observed -log10 p-value
Expected -log10 p-value
no hit
1 2 3 4 5 6 7 8 9 10 11 12 13 14 15 16 17 18 19 X

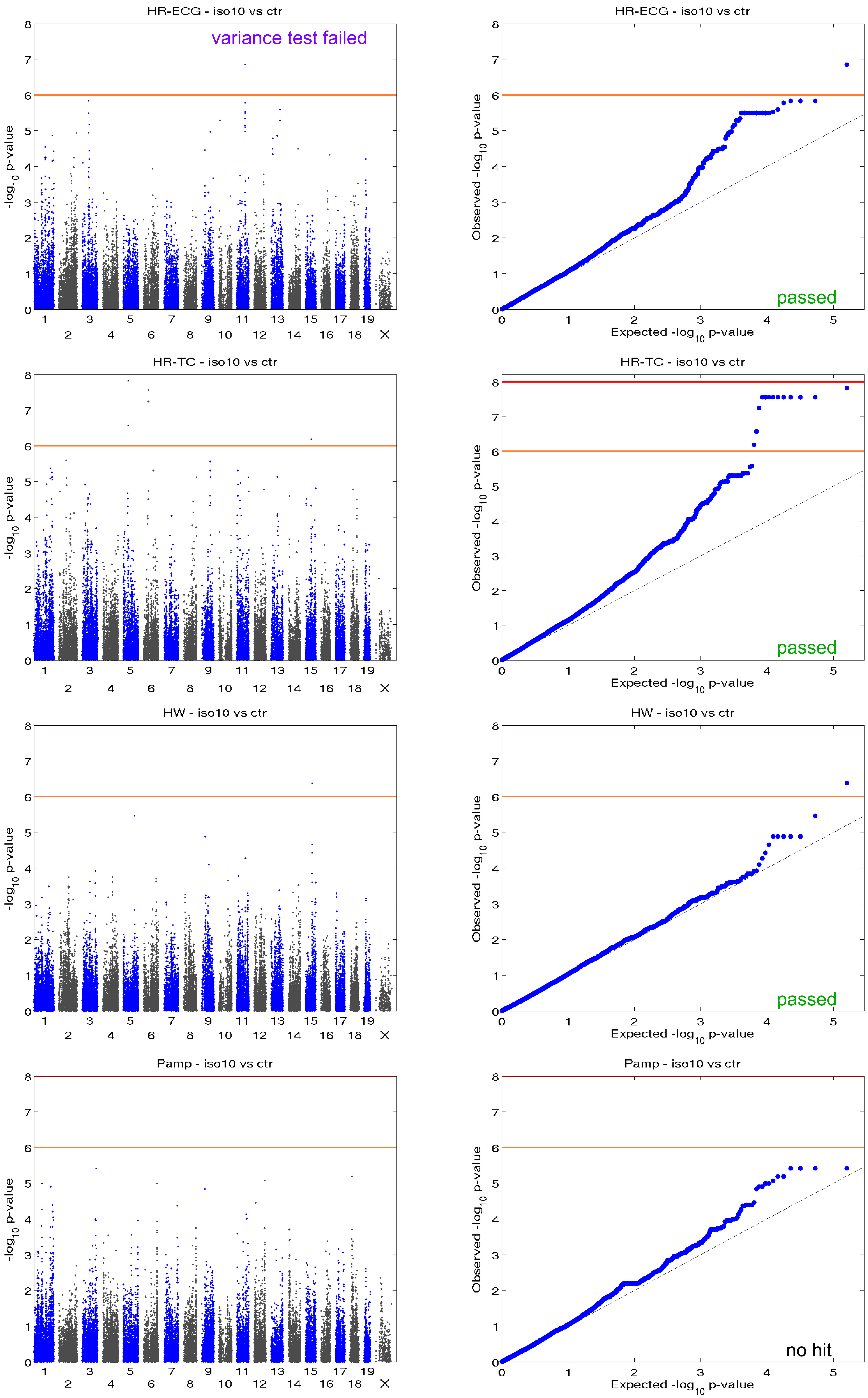

HR-ECG - iso10 vs ctr
variance test failed
HR-ECG - iso10 vs ctr
passed
HR-TC - iso10 vs ctr
HR-TC - iso10 vs ctr
passed
HW - iso10 vs ctr
HW - iso10 vs ctr
passed
Pamp - iso10 vs ctr
Pamp - iso10 vs ctr
no hit
-log10 p-value
Observed -log10 p-value
Expected -log10 p-value
1 2 3 4 5 6 7 8 9 10 11 12 13 14 15 16 17 18 19 X

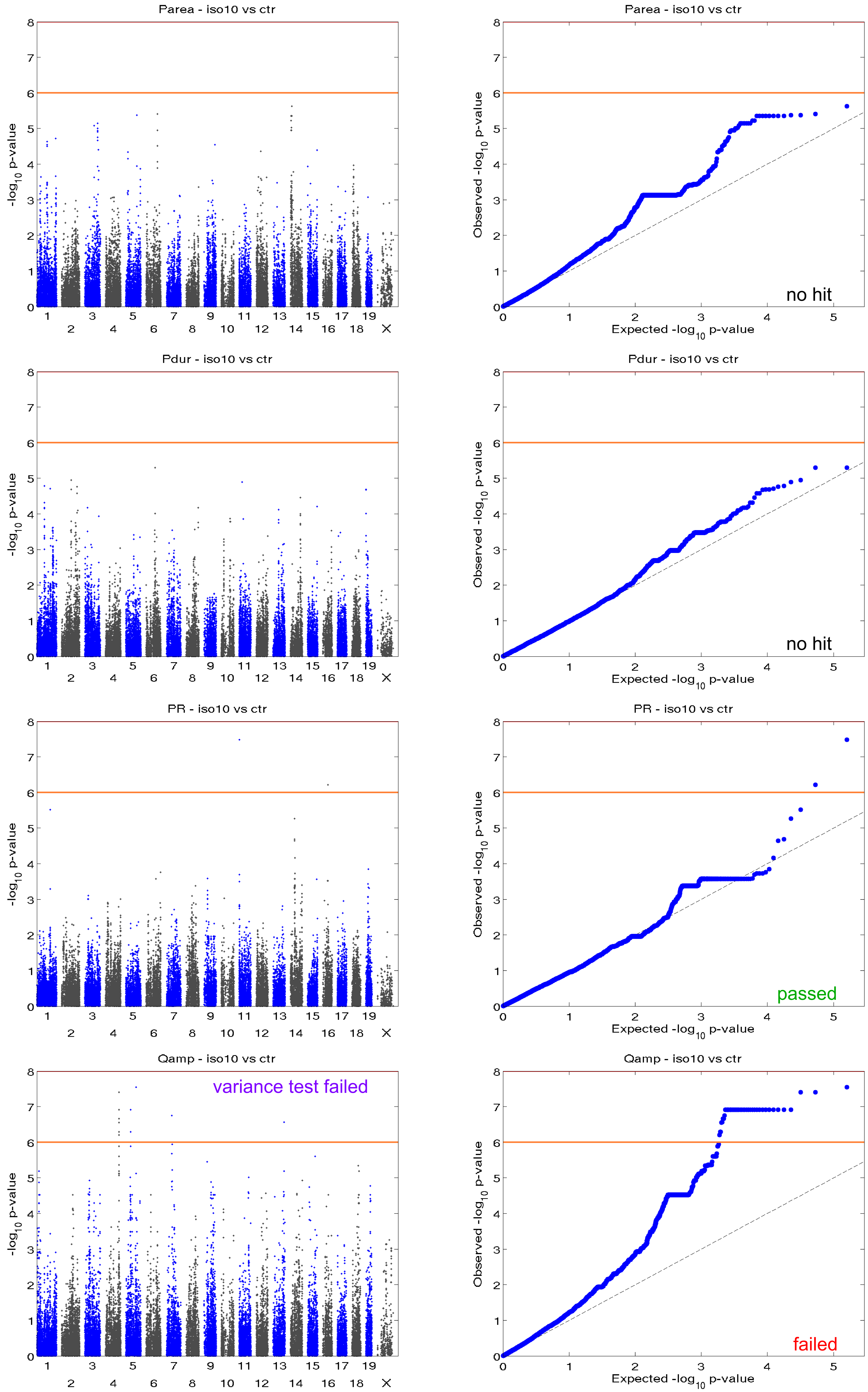

Parea - iso10 vs ctr
-log10 p-value
Parea - iso10 vs ctr
Observed -log10 p-value
Expected -log10 p-value
no hit
Pdur - iso10 vs ctr
-log10 p-value
Pdur - iso10 vs ctr
Observed -log10 p-value
Expected -log10 p-value
no hit
PR - iso10 vs ctr
-log10 p-value
PR - iso10 vs ctr
Observed -log10 p-value
Expected -log10 p-value
passed
Qamp - iso10 vs ctr
variance test failed
-log10 p-value
Qamp - iso10 vs ctr
Observed -log10 p-value
Expected -log10 p-value
failed
1 2 3 4 5 6 7 8 9 10 11 12 13 14 15 16 17 18 19 X

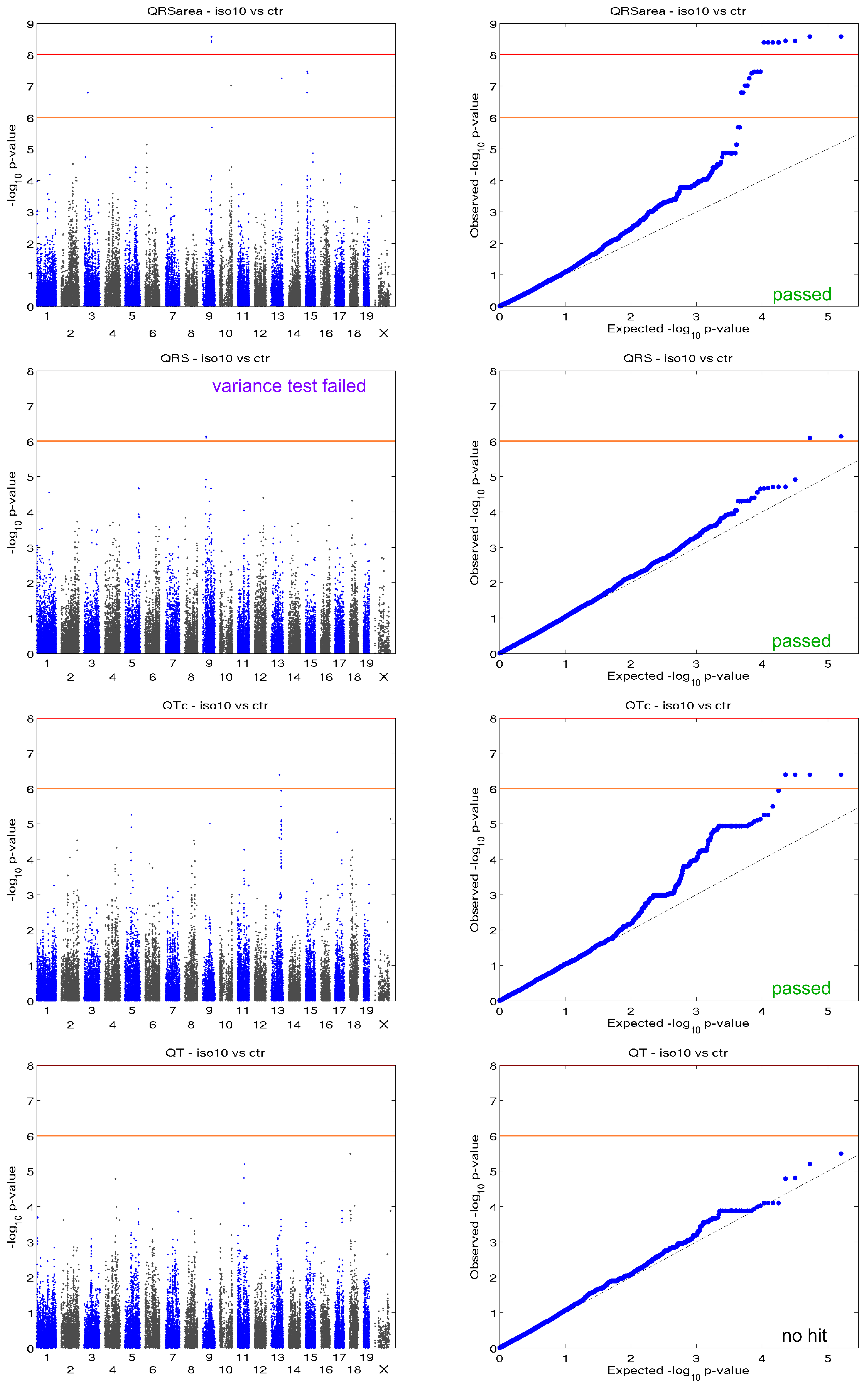
QRSarea - iso10 vs ctr
passed
QRS - iso10 vs ctr
variance test failed
passed
QTc - iso10 vs ctr
passed
QT - iso10 vs ctr
no hit
-log10 p-value
Observed -log10 p-value
Expected -log10 p-value

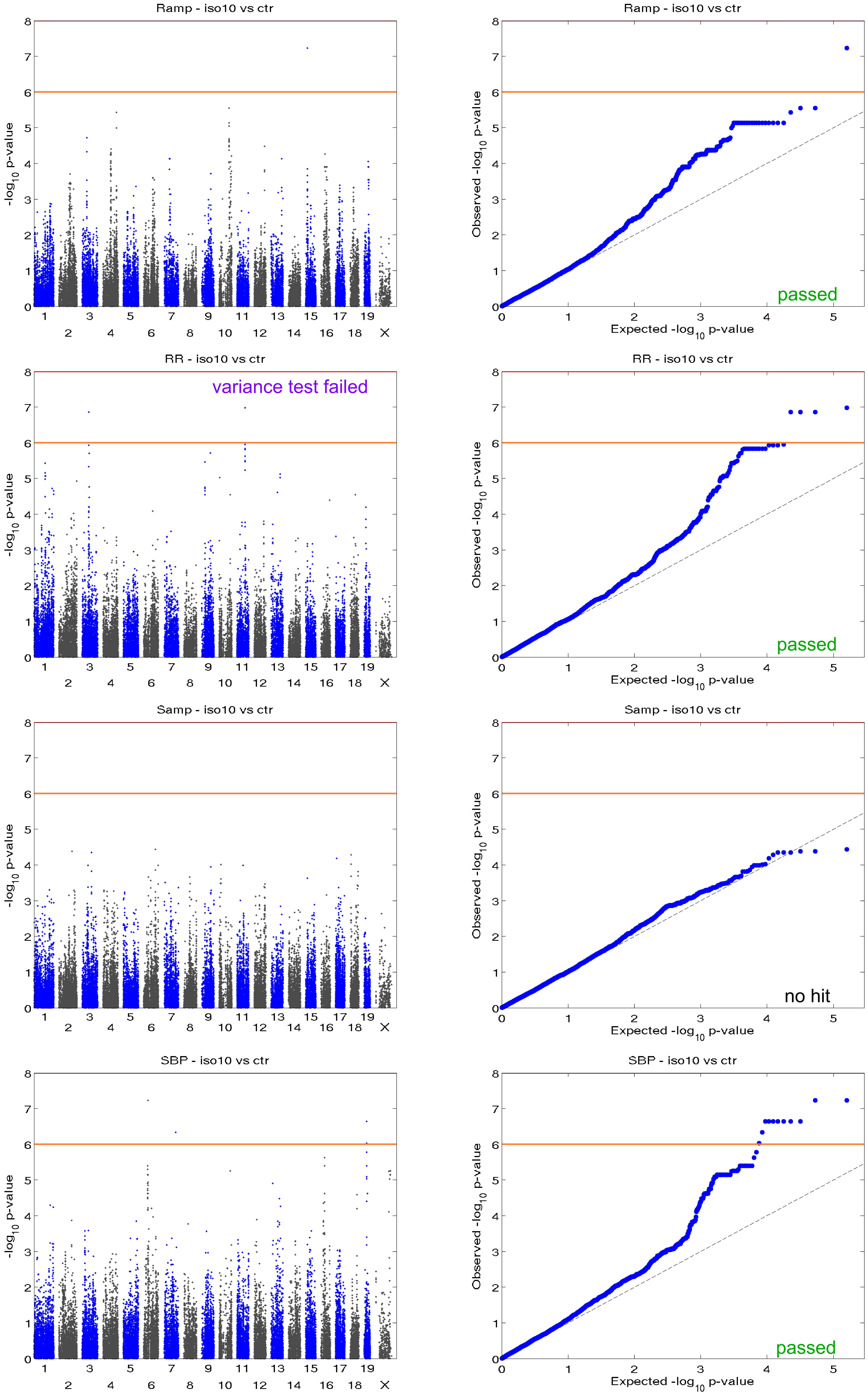
Ramp - iso10 vs ctr
-log10 p-value
Ramp - iso10 vs ctr
Observed -log10 p-value
Expected -log10 p-value
passed
RR - iso10 vs ctr
variance test failed
-log10 p-value
RR - iso10 vs ctr
Observed -log10 p-value
Expected -log10 p-value
passed
Samp - iso10 vs ctr
-log10 p-value
Samp - iso10 vs ctr
Observed -log10 p-value
Expected -log10 p-value
no hit
SBP - iso10 vs ctr
-log10 p-value
SBP - iso10 vs ctr
Observed -log10 p-value
Expected -log10 p-value
passed

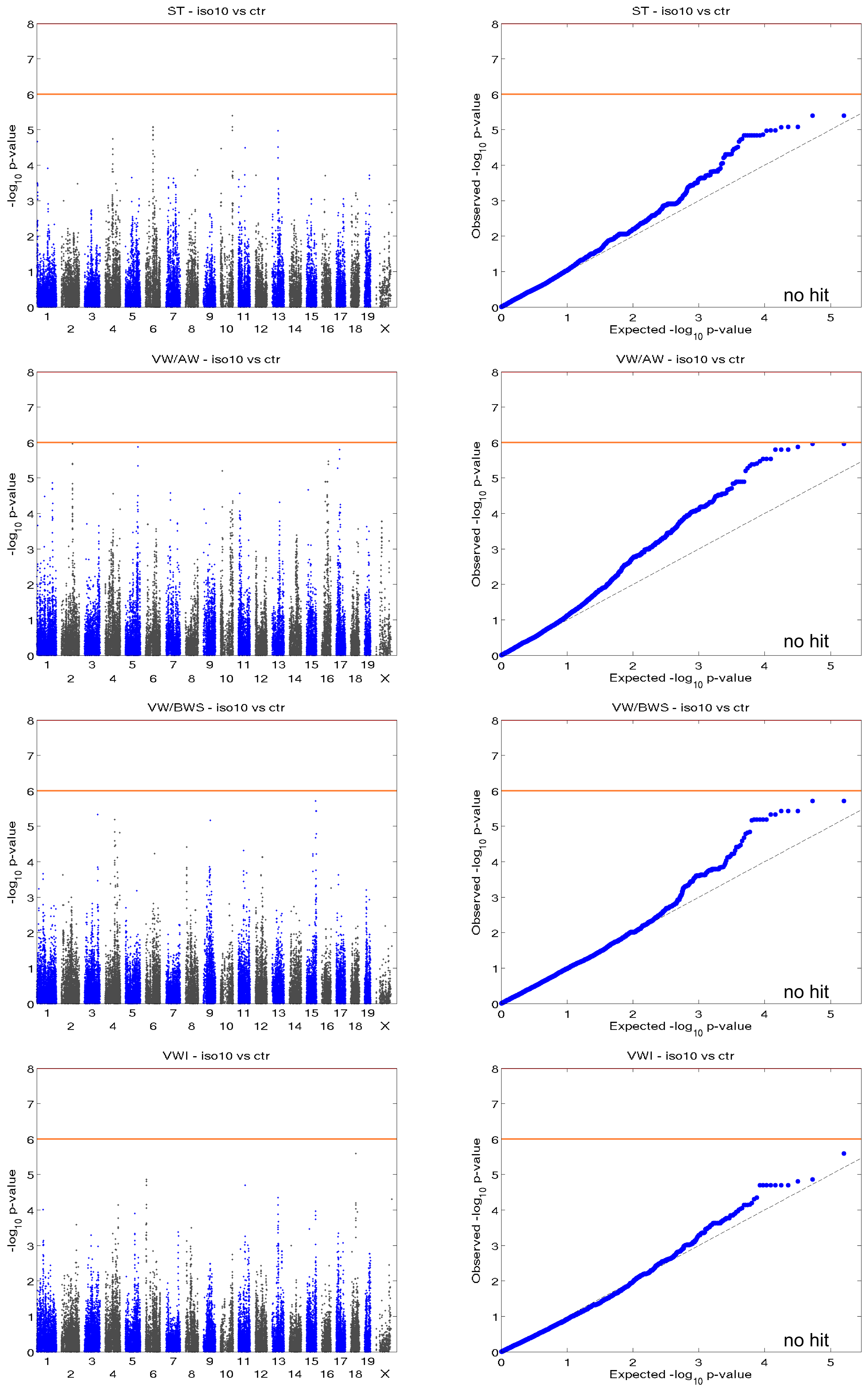
ST - iso10 vs ctr
-log10 p-value
1 2 3 4 5 6 7 8 9 10 11 12 13 14 15 16 17 18 19 X
ST - iso10 vs ctr
Observed -log10 p-value
Expected -log10 p-value
no hit
VW/AW - iso10 vs ctr
-log10 p-value
1 2 3 4 5 6 7 8 9 10 11 12 13 14 15 16 17 18 19 X
VW/AW - iso10 vs ctr
Observed -log10 p-value
Expected -log10 p-value
no hit
VW/BWS - iso10 vs ctr
-log10 p-value
1 2 3 4 5 6 7 8 9 10 11 12 13 14 15 16 17 18 19 X
VW/BWS - iso10 vs ctr
Observed -log10 p-value
Expected -log10 p-value
no hit
VWI - iso10 vs ctr
-log10 p-value
1 2 3 4 5 6 7 8 9 10 11 12 13 14 15 16 17 18 19 X
VWI - iso10 vs ctr
Observed -log10 p-value
Expected -log10 p-value
no hit

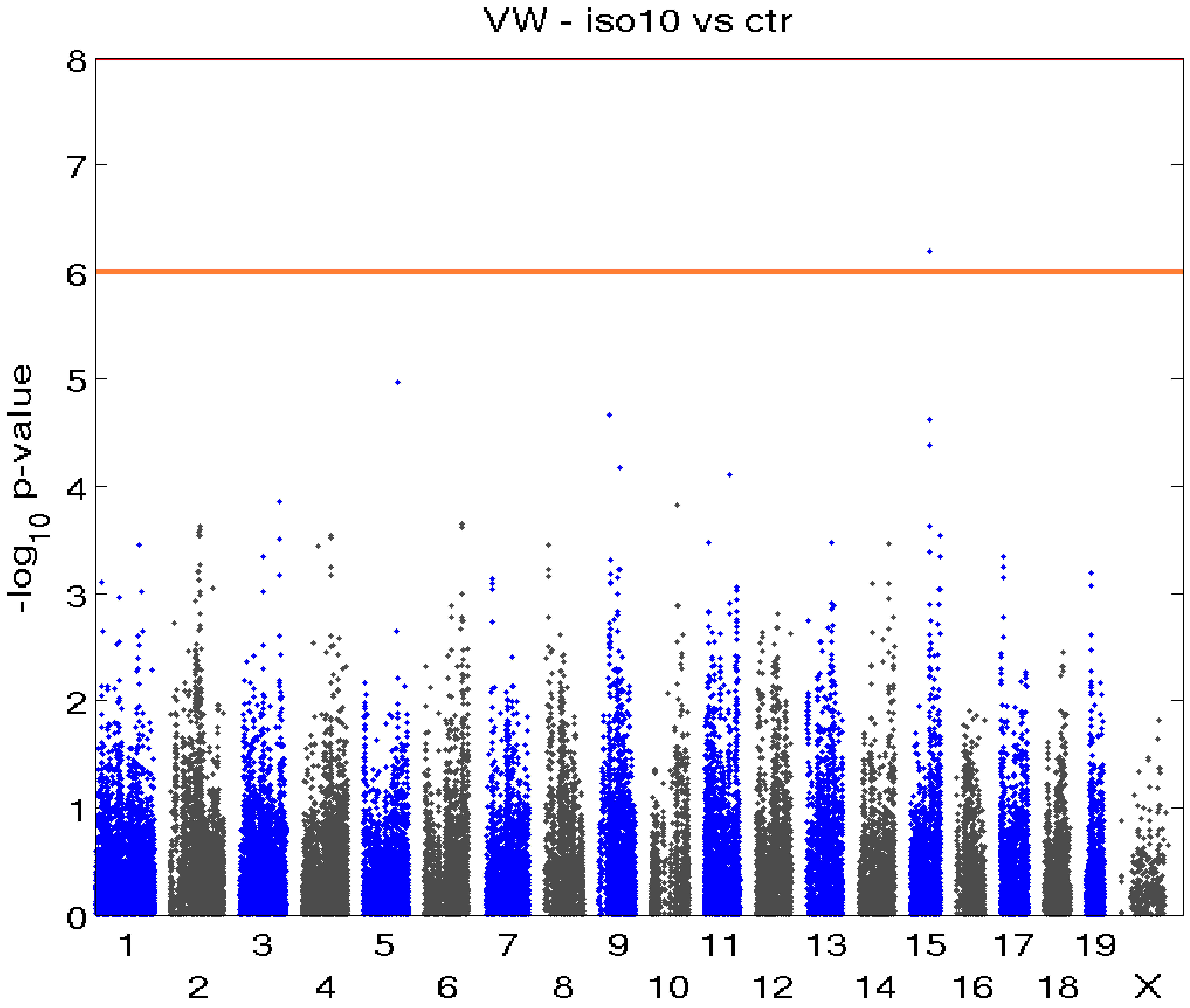
VW - iso10 vs ctr
-log10 p-value
0
1
2
3
4
5
6
7
8
1
2
3
4
5
6
7
8
9
10
11
12
13
14
15
16
17
18
19
X

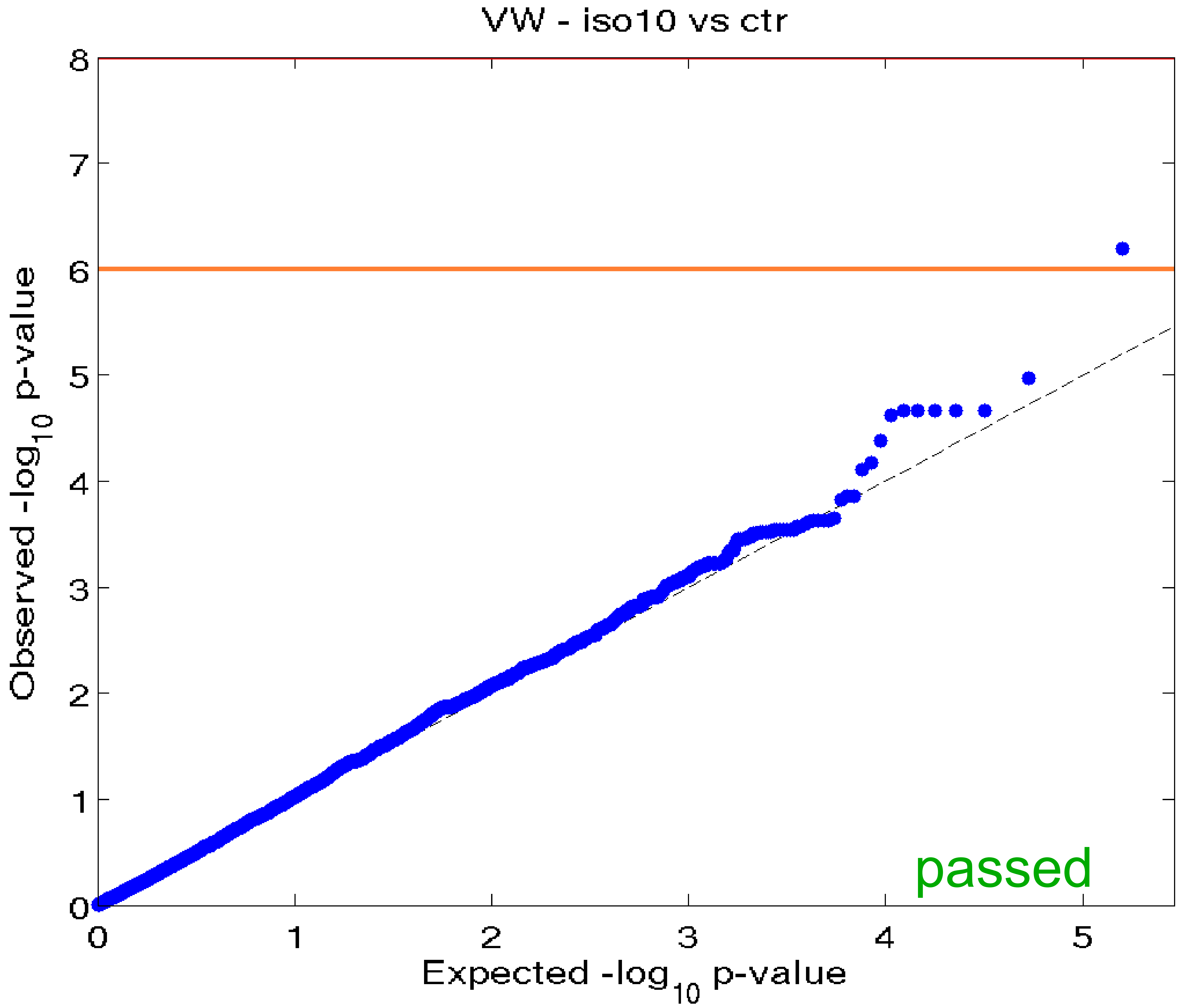
VW - iso10 vs ctr
Observed -log10 p-value
Expected -log10 p-value
0
1
2
3
4
5
6
7
8
0
1
2
3
4
5
passed
