# Supplementary material for: Mapping Genetic Variants Associated with Beta-Adrenergic Responses in Inbred Mice
Source: PLoS One. 2012 Jul 31;7(7):e41032. doi: 10.1371/journal.pone.0041032 (PMC3409184; doi:10.1371/journal.pone.0041032)
Supplement: Figure S7 — Manhattan and QQ-plots for the effects of iso10 vs ate treatments on 25 traits. QQ-plot-based quality control is indicated as “passed” or failed”. Phenotypes for which any of the differences between an individual trait value and its matching mean strain value exceeded 3 SD are labelled as “var test failed”. (PDF) [file pone.0041032.s007.pdf]

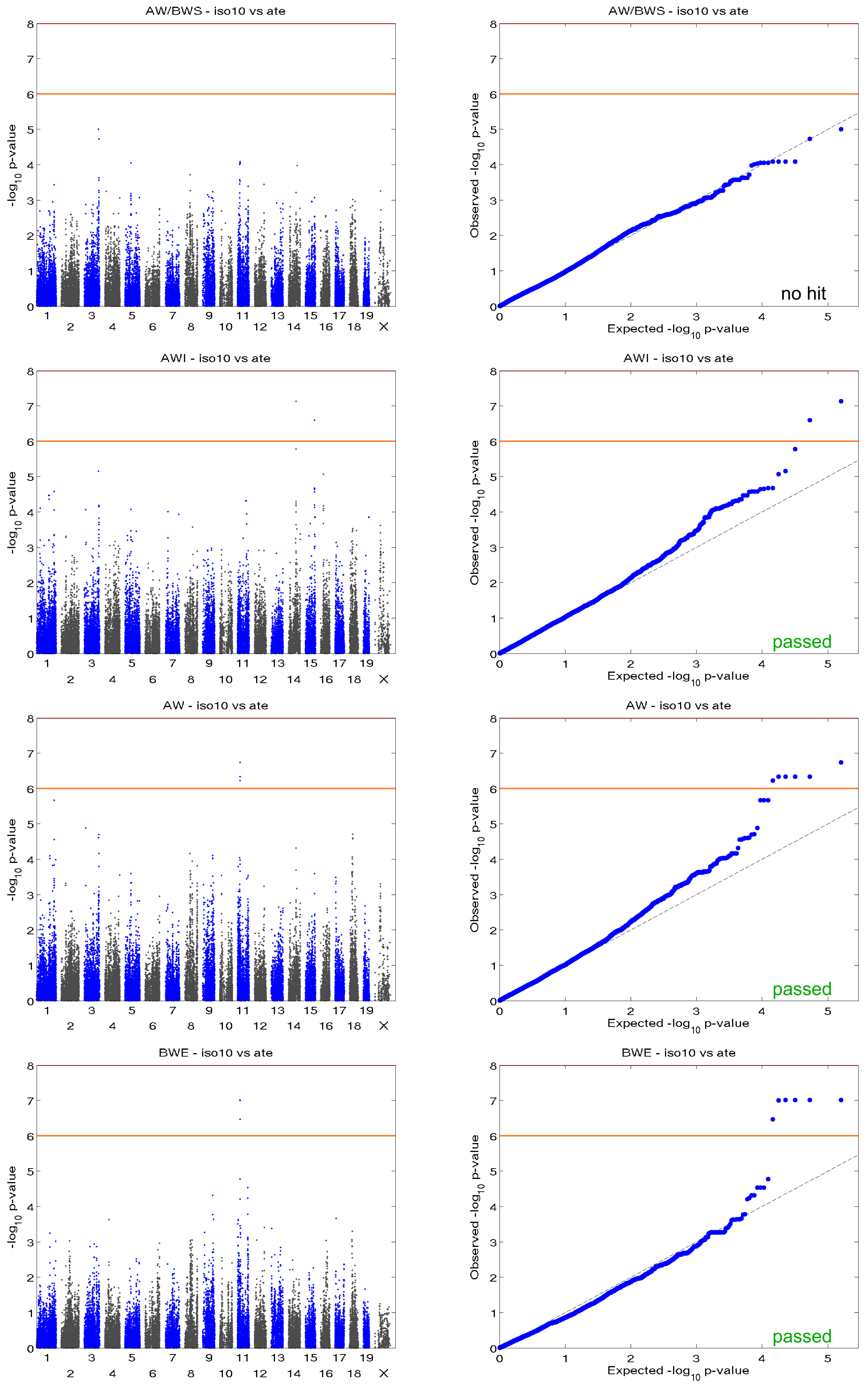

AW/BWS - iso10 vs ate
AWI - iso10 vs ate
AW - iso10 vs ate
BWE - iso10 vs ate
-log10 p-value
Observed -log10 p-value
Expected -log10 p-value
1 2 3 4 5 6 7 8 9 10 11 12 13 14 15 16 17 18 19 X
no hit
passed
passed
passed

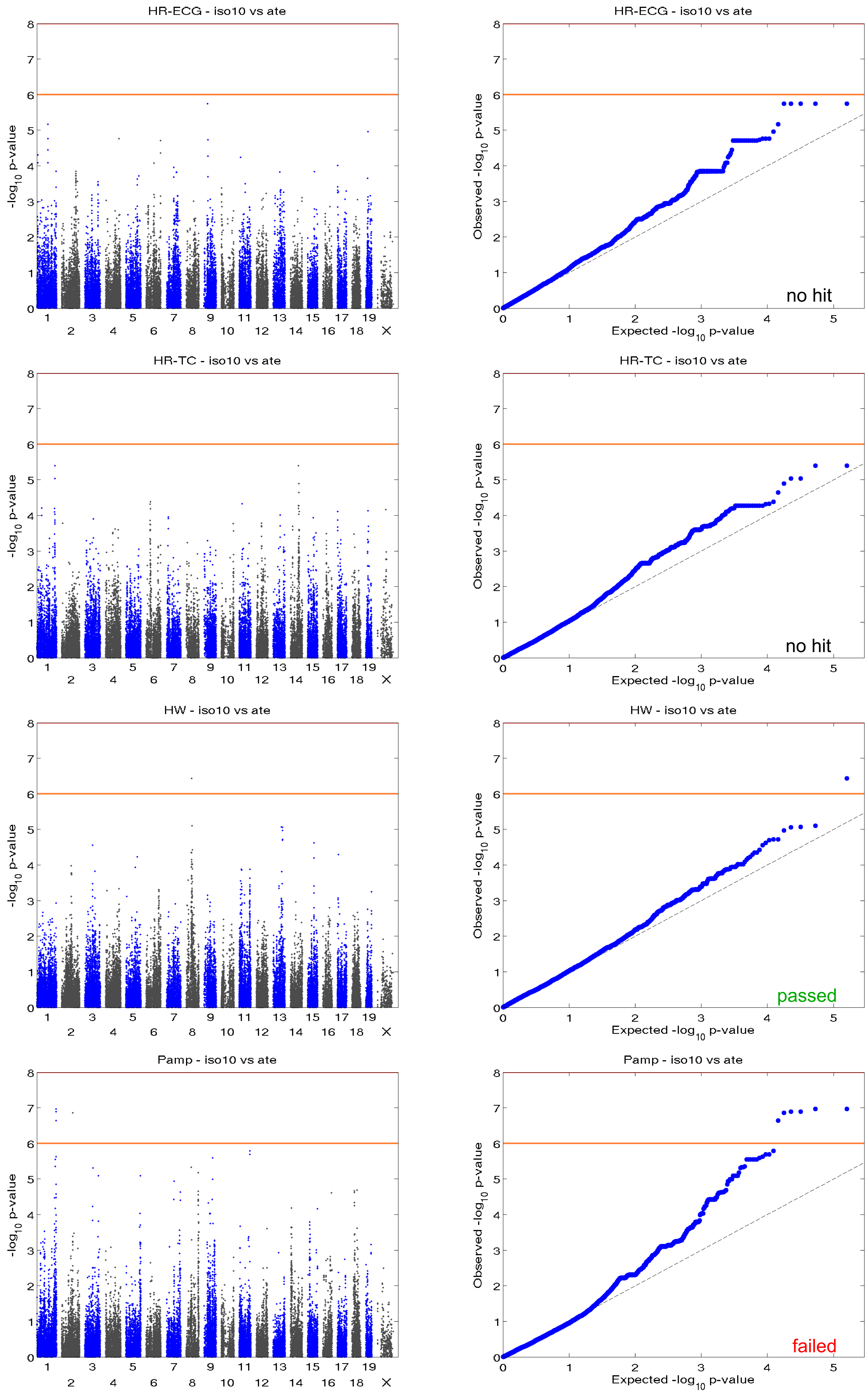

HR-ECG - iso10 vs ate
-log10 p-value
HR-ECG - iso10 vs ate
Observed -log10 p-value
Expected -log10 p-value
no hit
HR-TC - iso10 vs ate
-log10 p-value
HR-TC - iso10 vs ate
Observed -log10 p-value
Expected -log10 p-value
no hit
HW - iso10 vs ate
-log10 p-value
HW - iso10 vs ate
Observed -log10 p-value
Expected -log10 p-value
passed
Pamp - iso10 vs ate
-log10 p-value
Pamp - iso10 vs ate
Observed -log10 p-value
Expected -log10 p-value
failed

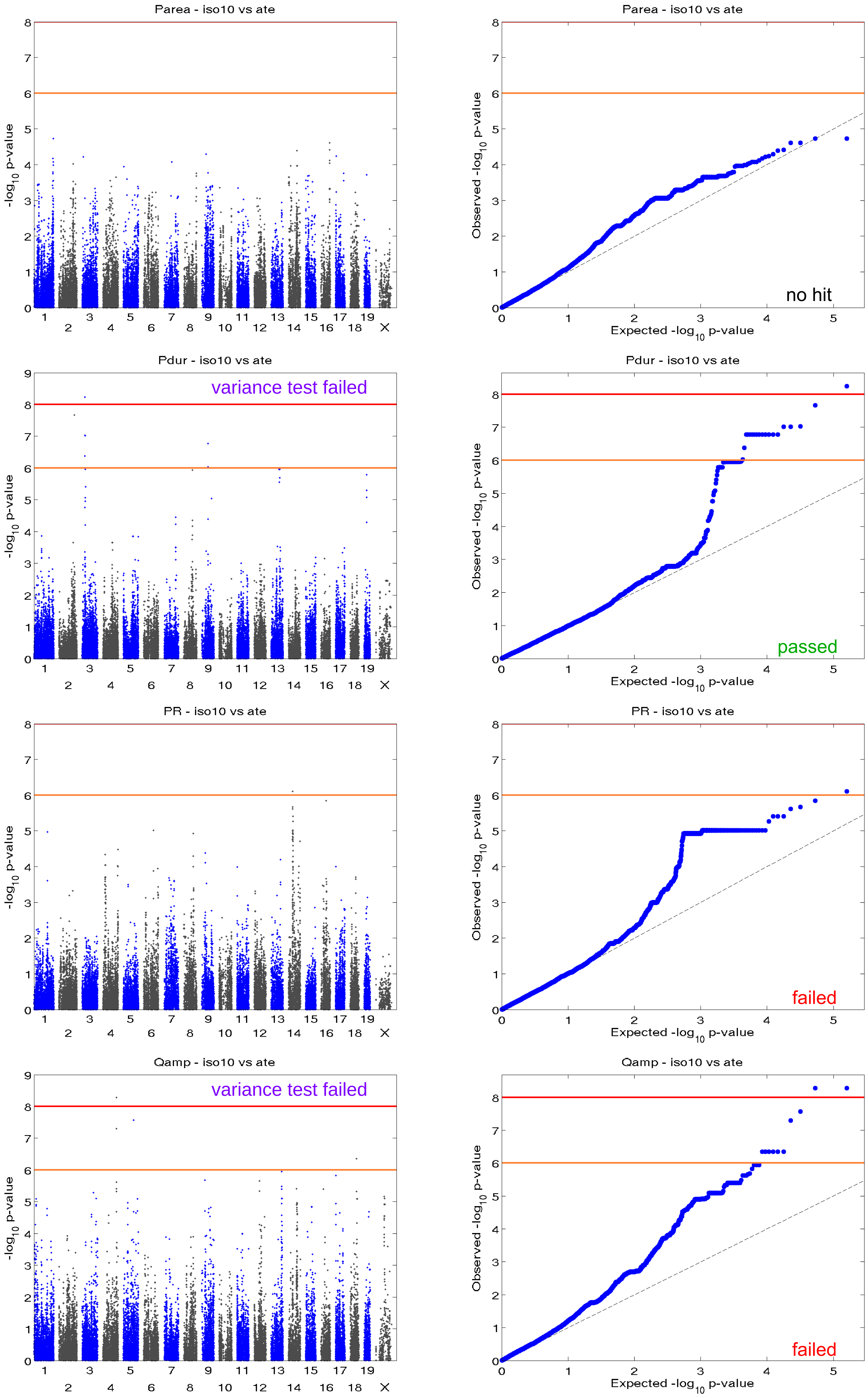

Parea - iso10 vs ate
-log10 p-value
Parea - iso10 vs ate
Observed -log10 p-value
Expected -log10 p-value
no hit
Pdur - iso10 vs ate
variance test failed
-log10 p-value
Pdur - iso10 vs ate
Observed -log10 p-value
Expected -log10 p-value
passed
PR - iso10 vs ate
-log10 p-value
PR - iso10 vs ate
Observed -log10 p-value
Expected -log10 p-value
failed
Qamp - iso10 vs ate
variance test failed
-log10 p-value
Qamp - iso10 vs ate
Observed -log10 p-value
Expected -log10 p-value
failed

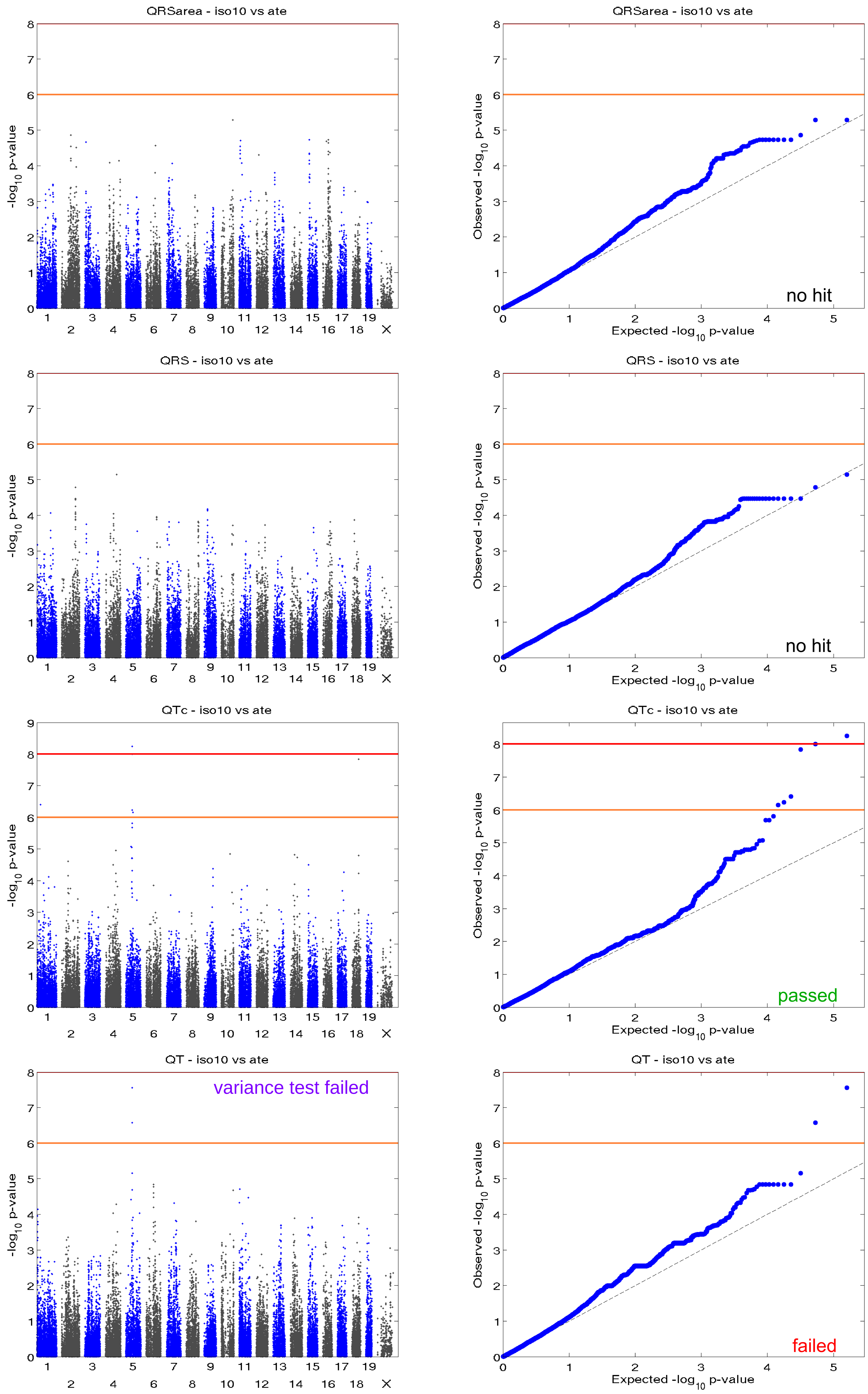
QRSarea - iso10 vs ate
-log10 p-value
QRSarea - iso10 vs ate
Observed -log10 p-value
Expected -log10 p-value
no hit
QRS - iso10 vs ate
-log10 p-value
QRS - iso10 vs ate
Observed -log10 p-value
Expected -log10 p-value
no hit
QTc - iso10 vs ate
-log10 p-value
QTc - iso10 vs ate
Observed -log10 p-value
Expected -log10 p-value
passed
QT - iso10 vs ate
variance test failed
-log10 p-value
QT - iso10 vs ate
Observed -log10 p-value
Expected -log10 p-value
failed
1 2 3 4 5 6 7 8 9 10 11 12 13 14 15 16 17 18 19 X

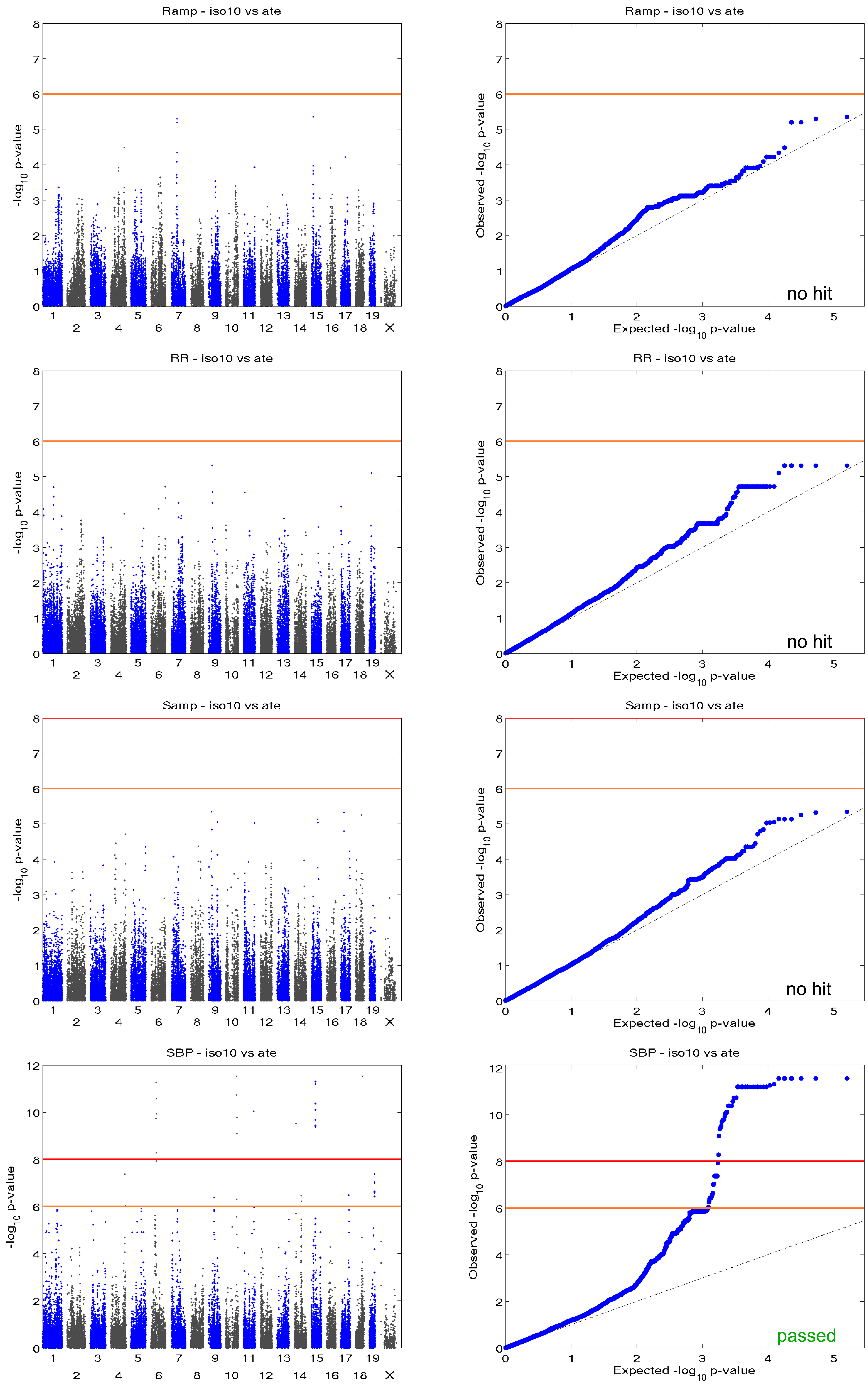
Ramp - iso10 vs ate
-log10 p-value
Ramp - iso10 vs ate
Observed -log10 p-value
Expected -log10 p-value
no hit
RR - iso10 vs ate
-log10 p-value
RR - iso10 vs ate
Observed -log10 p-value
Expected -log10 p-value
no hit
Samp - iso10 vs ate
-log10 p-value
Samp - iso10 vs ate
Observed -log10 p-value
Expected -log10 p-value
no hit
SBP - iso10 vs ate
-log10 p-value
SBP - iso10 vs ate
Observed -log10 p-value
Expected -log10 p-value
passed

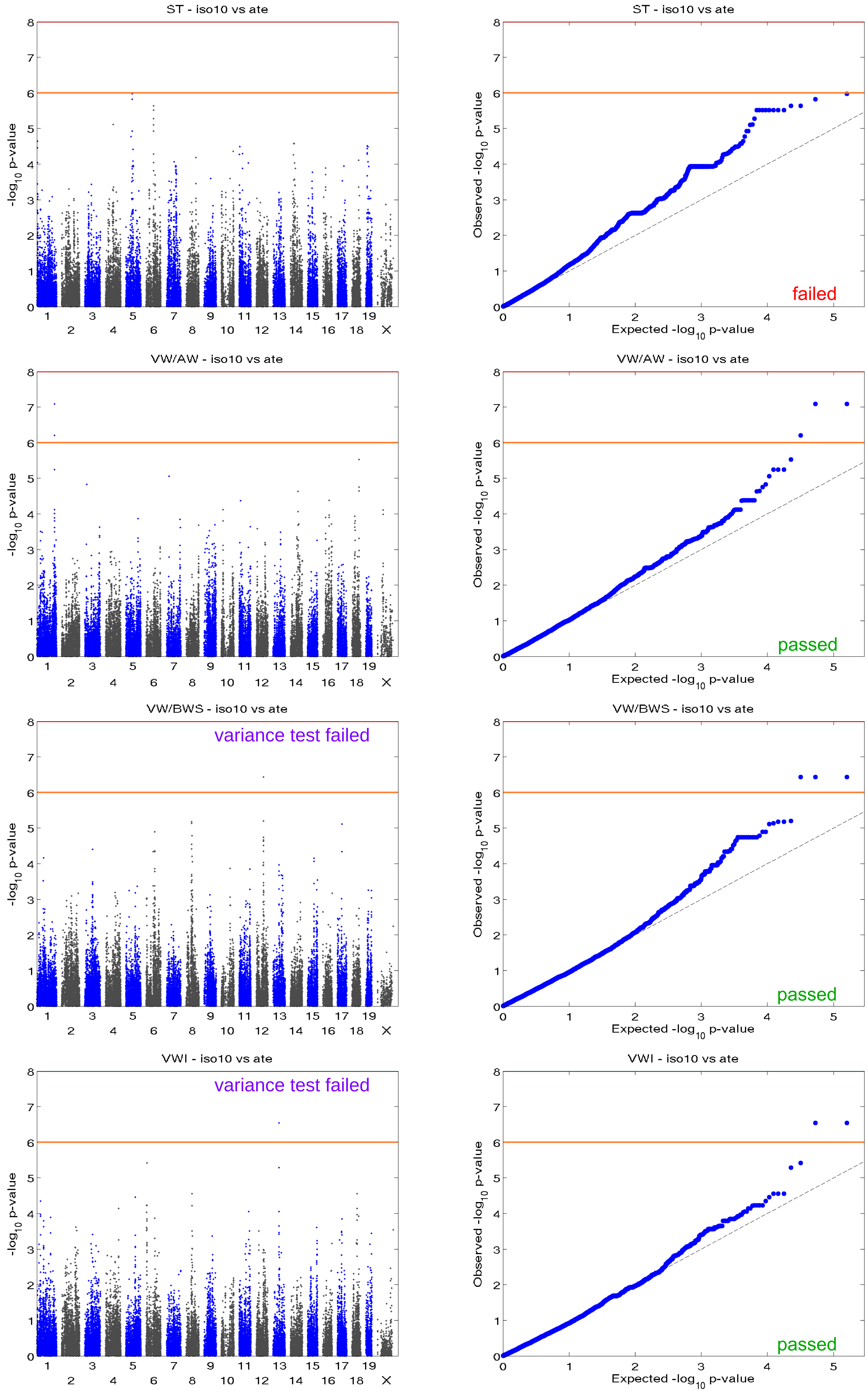
ST - iso10 vs ate
-log10 p-value
1 2 3 4 5 6 7 8 9 10 11 12 13 14 15 16 17 18 19 X
ST - iso10 vs ate
Observed -log10 p-value
Expected -log10 p-value
failed
VW/AW - iso10 vs ate
-log10 p-value
VW/AW - iso10 vs ate
Observed -log10 p-value
Expected -log10 p-value
passed
VW/BWS - iso10 vs ate
variance test failed
-log10 p-value
VW/BWS - iso10 vs ate
Observed -log10 p-value
Expected -log10 p-value
passed
VWI - iso10 vs ate
variance test failed
-log10 p-value
VWI - iso10 vs ate
Observed -log10 p-value
Expected -log10 p-value
passed

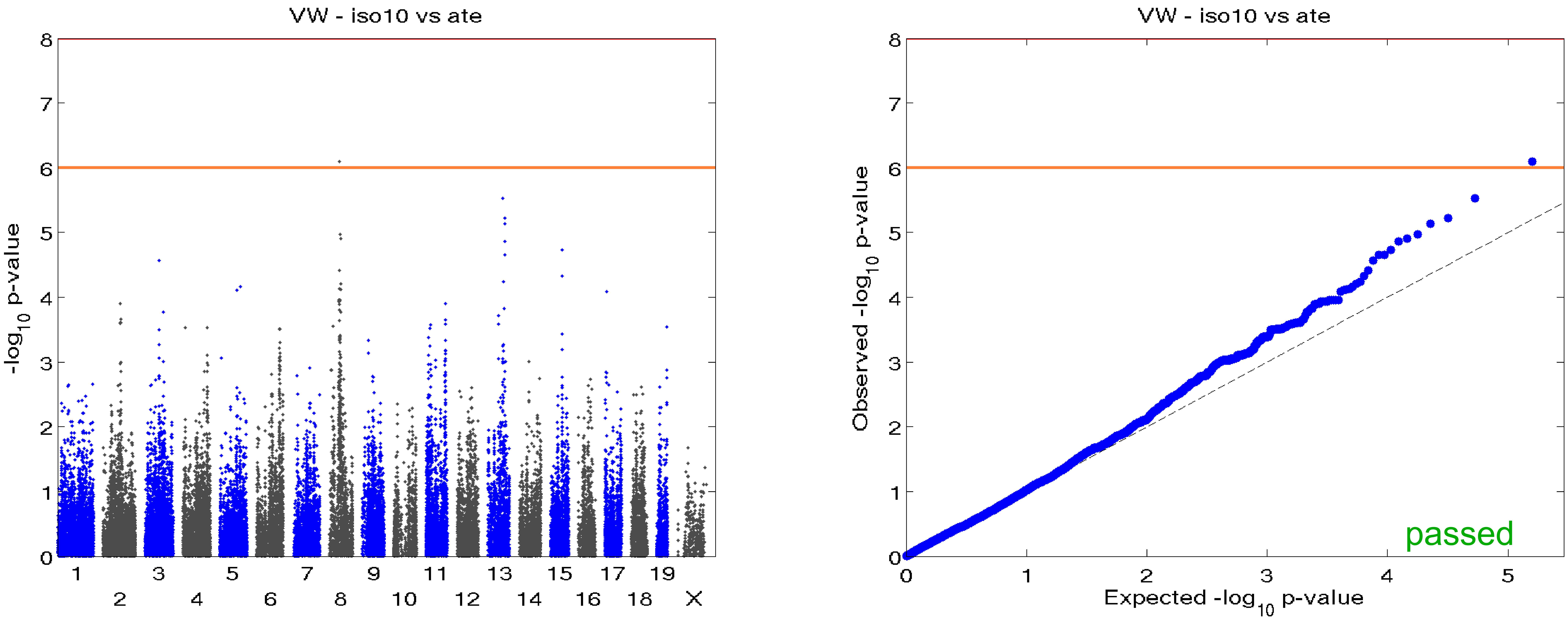

VW - iso10 vs ate
-log10 p-value
1 2 3 4 5 6 7 8 9 10 11 12 13 14 15 16 17 18 19 X
VW - iso10 vs ate
Observed -log10 p-value
Expected -log10 p-value
passed
