# Supplementary material for: Mapping Genetic Variants Associated with Beta-Adrenergic Responses in Inbred Mice
Source: PLoS One. 2012 Jul 31;7(7):e41032. doi: 10.1371/journal.pone.0041032 (PMC3409184; doi:10.1371/journal.pone.0041032)
Supplement: Figure S8 — Manhattan and QQ-plots for the effects of ate vs iso10 treatments on 25 traits. QQ-plot-based quality control is indicated as “passed” or failed”. Phenotypes for which any of the differences between an individual trait value and its matching mean strain value exceeded 3 SD are labelled as “var test failed”. (PDF) [file pone.0041032.s008.pdf]

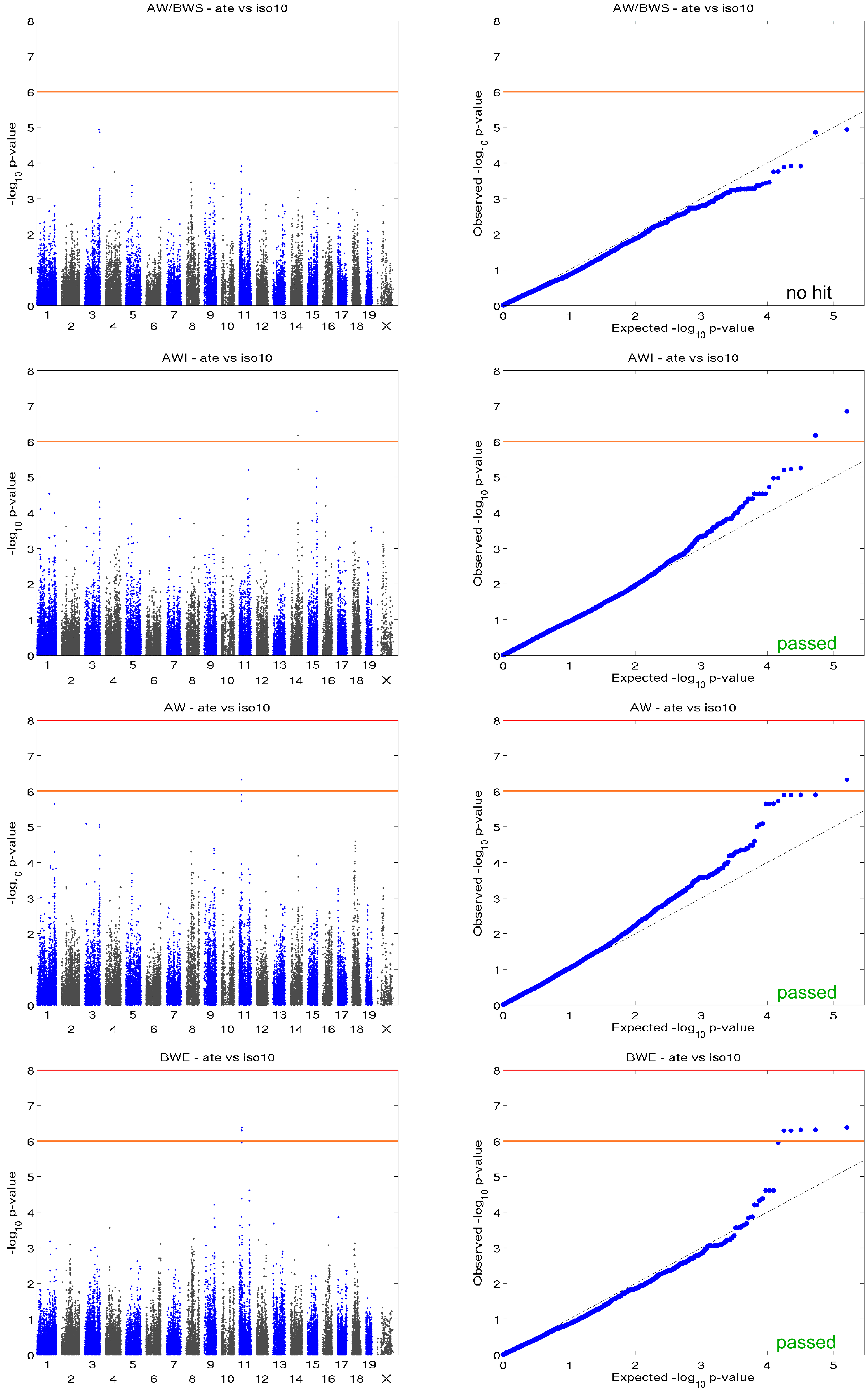
AW/BWS - ate vs iso10
-log10 p-value
AW/BWS - ate vs iso10
Observed -log10 p-value
Expected -log10 p-value
no hit
AWI - ate vs iso10
-log10 p-value
AWI - ate vs iso10
Observed -log10 p-value
Expected -log10 p-value
passed
AW - ate vs iso10
-log10 p-value
AW - ate vs iso10
Observed -log10 p-value
Expected -log10 p-value
passed
BWE - ate vs iso10
-log10 p-value
BWE - ate vs iso10
Observed -log10 p-value
Expected -log10 p-value
passed

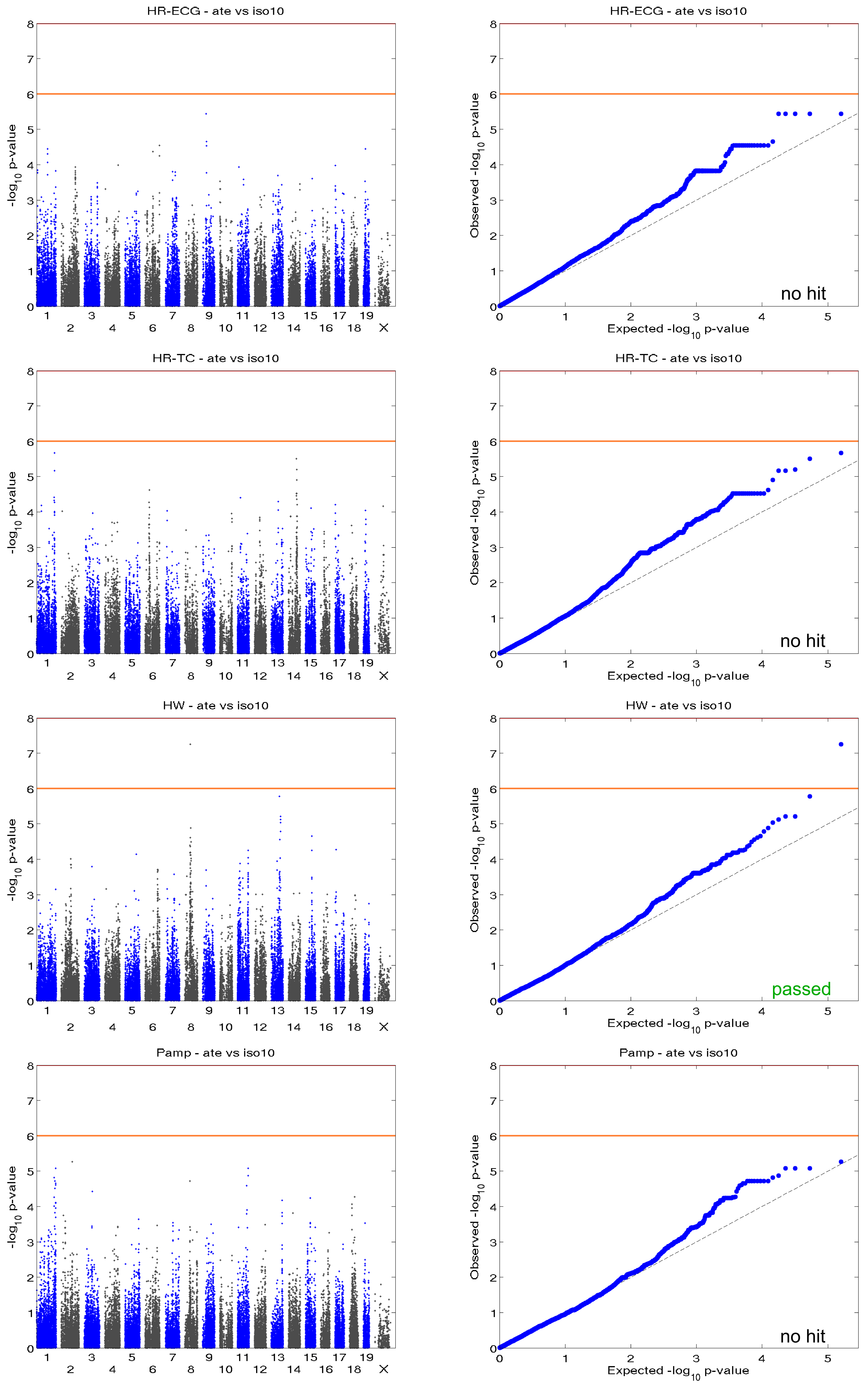
HR-ECG - ate vs iso10
HR-TC - ate vs iso10
HW - ate vs iso10
Pamp - ate vs iso10
-log10 p-value
Observed -log10 p-value
Expected -log10 p-value
1 2 3 4 5 6 7 8 9 10 11 12 13 14 15 16 17 18 19 X
no hit
no hit
passed
no hit

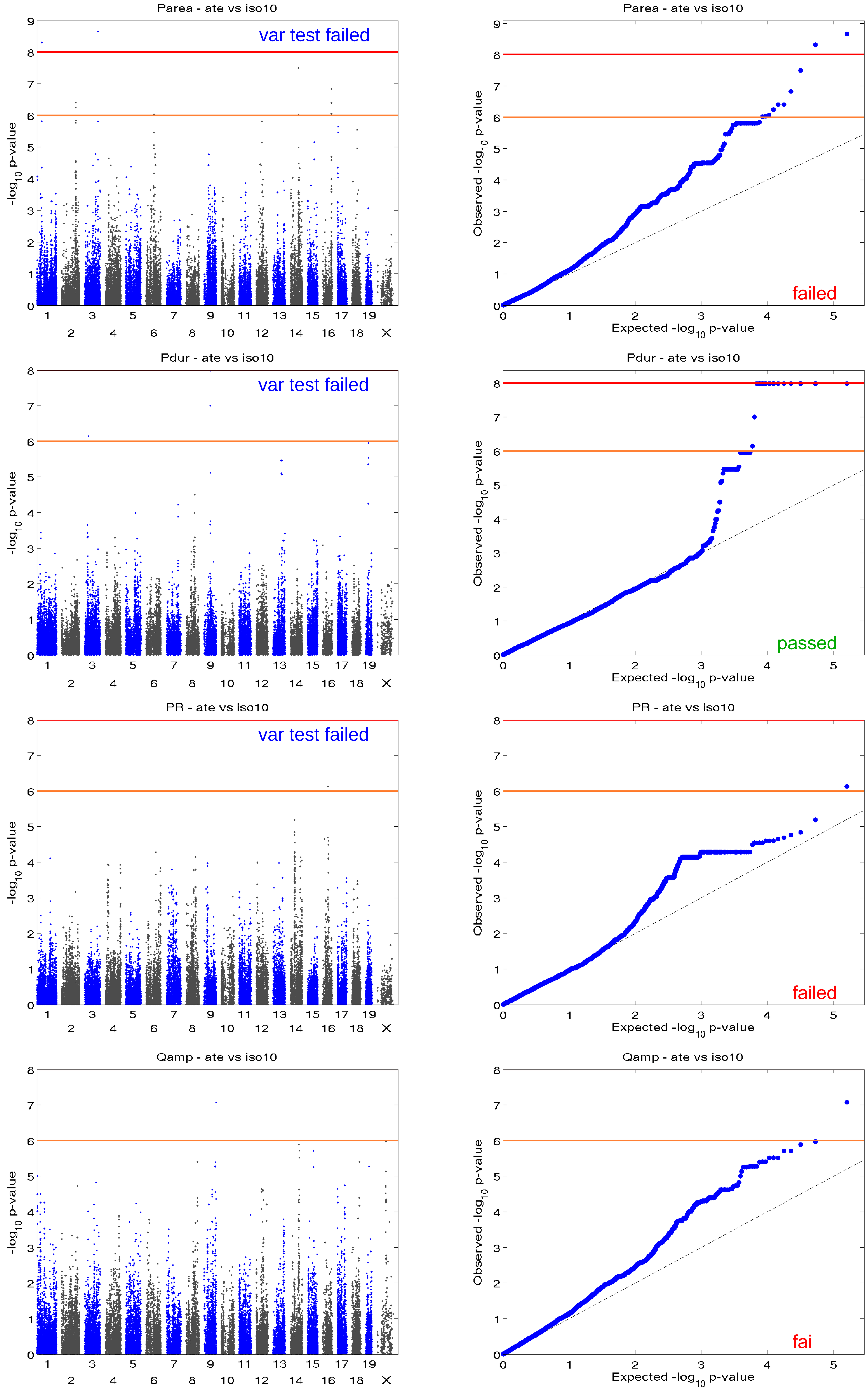
Parea - ate vs iso10
var test failed
-log10 p-value
Parea - ate vs iso10
Observed -log10 p-value
Expected -log10 p-value
failed
Pdur - ate vs iso10
var test failed
-log10 p-value
Pdur - ate vs iso10
Observed -log10 p-value
Expected -log10 p-value
passed
PR - ate vs iso10
var test failed
-log10 p-value
PR - ate vs iso10
Observed -log10 p-value
Expected -log10 p-value
failed
Qamp - ate vs iso10
-log10 p-value
Qamp - ate vs iso10
Observed -log10 p-value
Expected -log10 p-value
fai

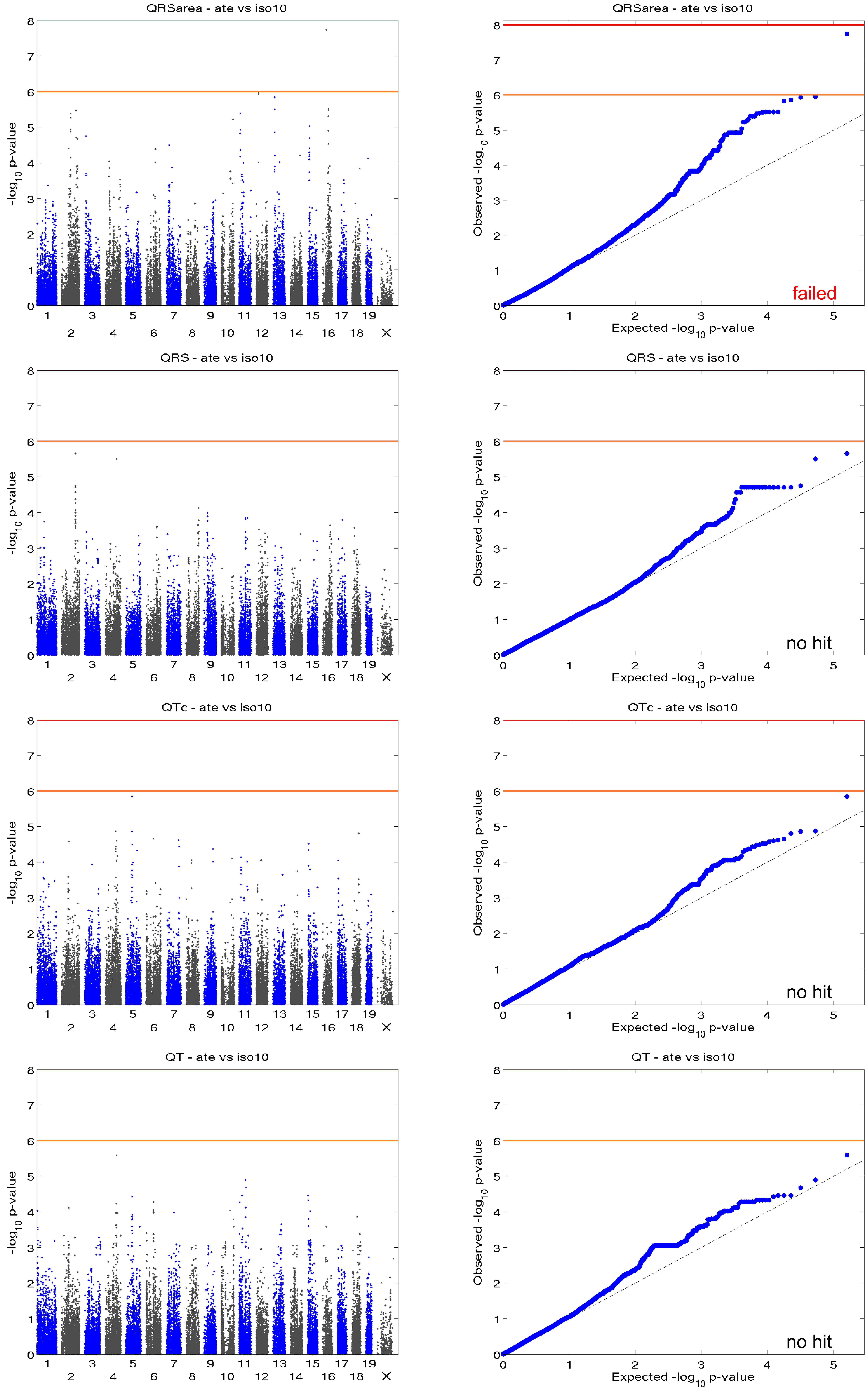
QRSarea - ate vs iso10
-log10 p-value
QRSarea - ate vs iso10
Observed -log10 p-value
Expected -log10 p-value
failed
QRS - ate vs iso10
-log10 p-value
QRS - ate vs iso10
Observed -log10 p-value
Expected -log10 p-value
no hit
QTc - ate vs iso10
-log10 p-value
QTc - ate vs iso10
Observed -log10 p-value
Expected -log10 p-value
no hit
QT - ate vs iso10
-log10 p-value
QT - ate vs iso10
Observed -log10 p-value
Expected -log10 p-value
no hit

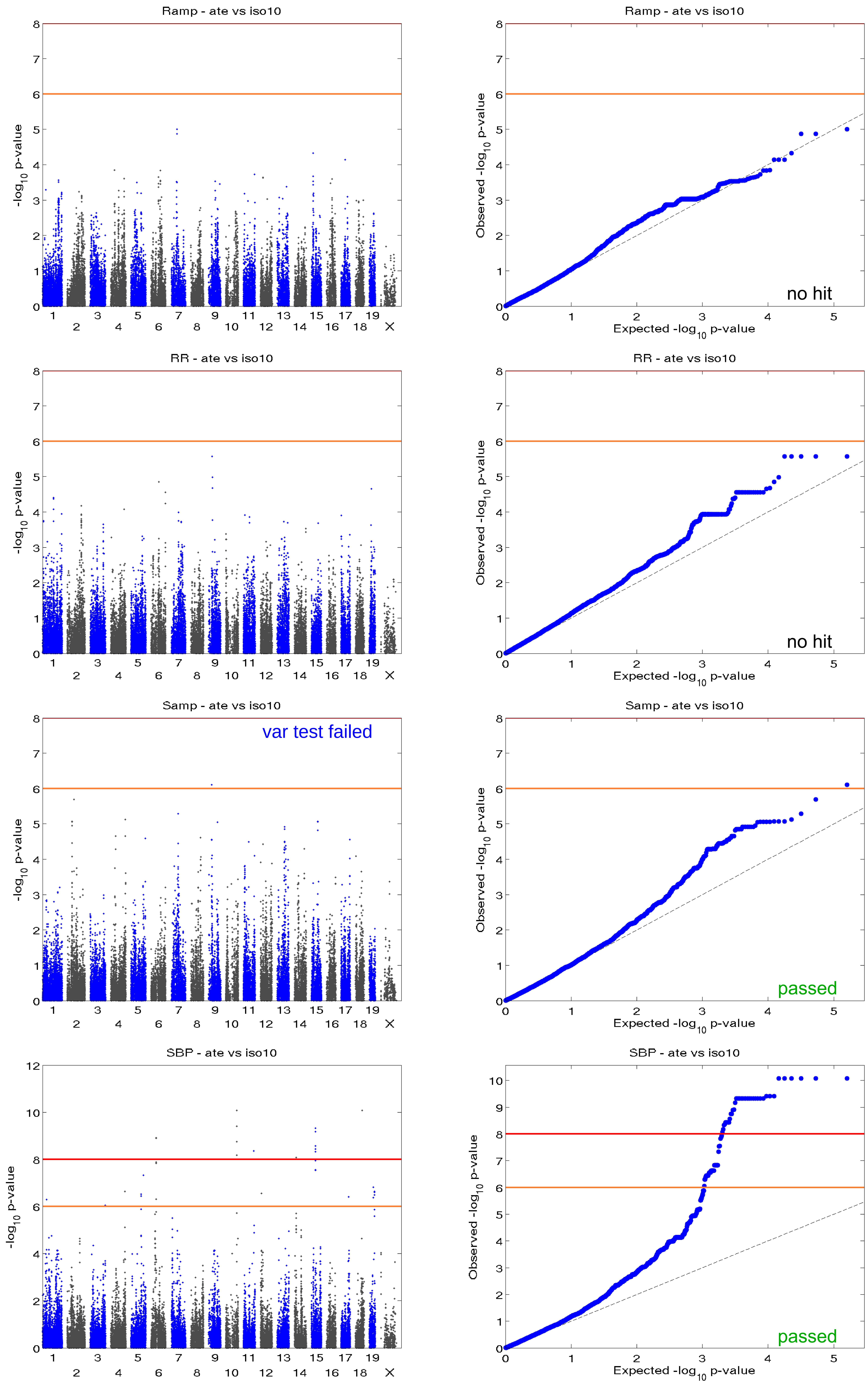

Ramp - ate vs iso10
-log10 p-value
Ramp - ate vs iso10
Observed -log10 p-value
Expected -log10 p-value
no hit
RR - ate vs iso10
-log10 p-value
RR - ate vs iso10
Observed -log10 p-value
Expected -log10 p-value
no hit
Samp - ate vs iso10
var test failed
-log10 p-value
Samp - ate vs iso10
Observed -log10 p-value
Expected -log10 p-value
passed
SBP - ate vs iso10
-log10 p-value
SBP - ate vs iso10
Observed -log10 p-value
Expected -log10 p-value
passed
1 2 3 4 5 6 7 8 9 10 11 12 13 14 15 16 17 18 19 X

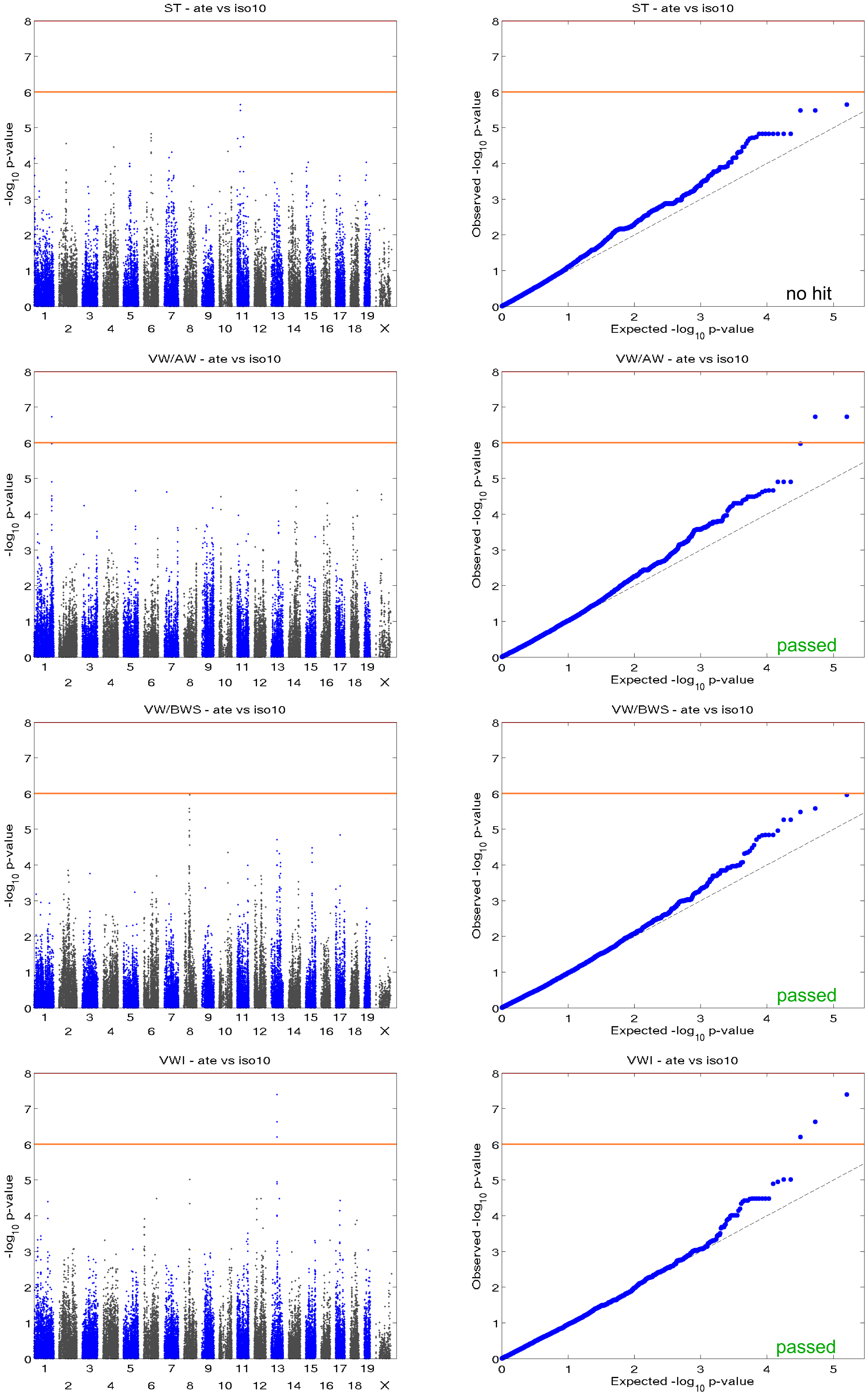
ST - ate vs iso10
-log10 p-value
ST - ate vs iso10
Observed -log10 p-value
Expected -log10 p-value
no hit
VW/AW - ate vs iso10
-log10 p-value
VW/AW - ate vs iso10
Observed -log10 p-value
Expected -log10 p-value
passed
VW/BWS - ate vs iso10
-log10 p-value
VW/BWS - ate vs iso10
Observed -log10 p-value
Expected -log10 p-value
passed
VWI - ate vs iso10
-log10 p-value
VWI - ate vs iso10
Observed -log10 p-value
Expected -log10 p-value
passed

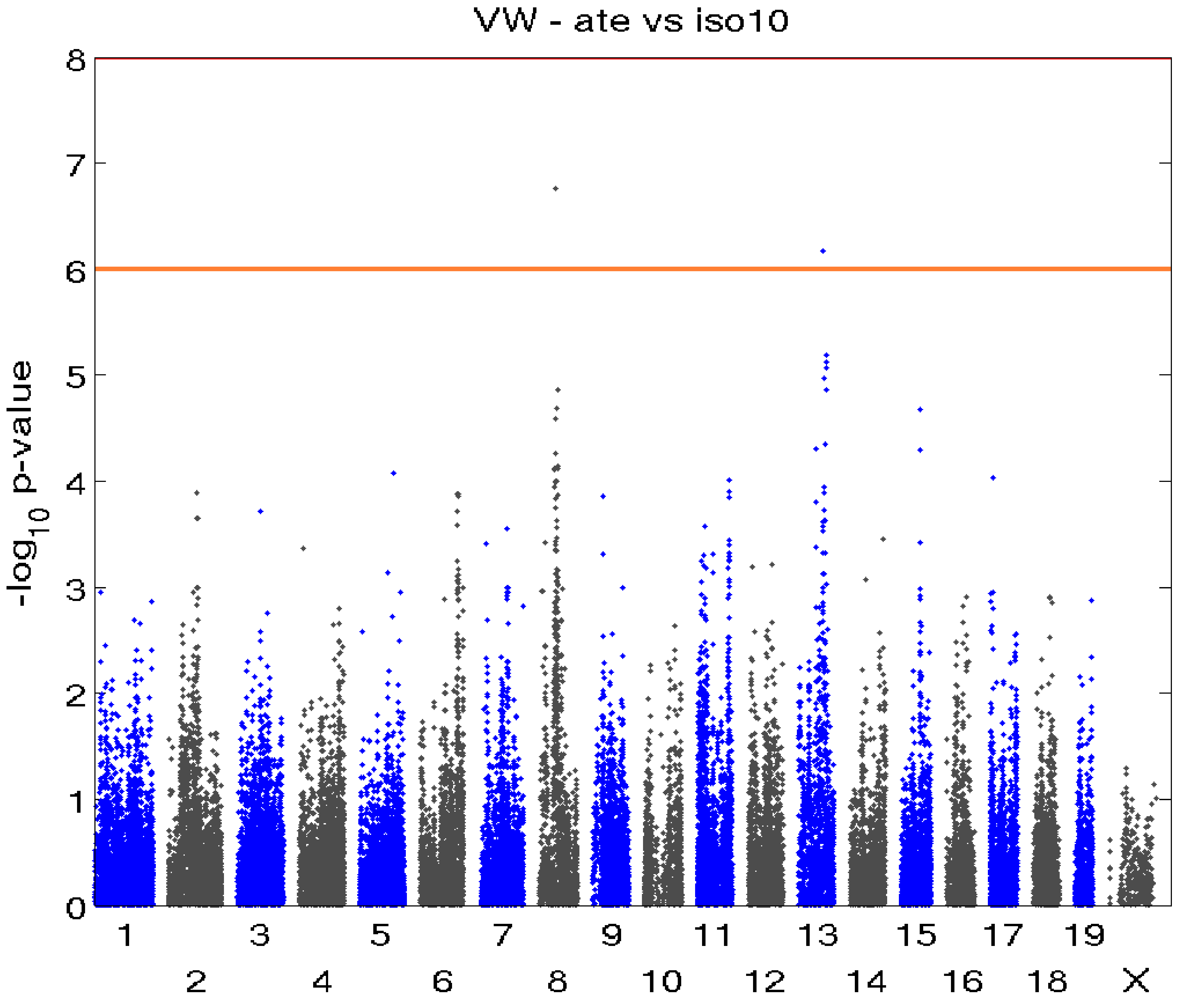
VW - ate vs iso10
-log10 p-value
8
7
6
5
4
3
2
1
0
1 2 3 4 5 6 7 8 9 10 11 12 13 14 15 16 17 18 19 X

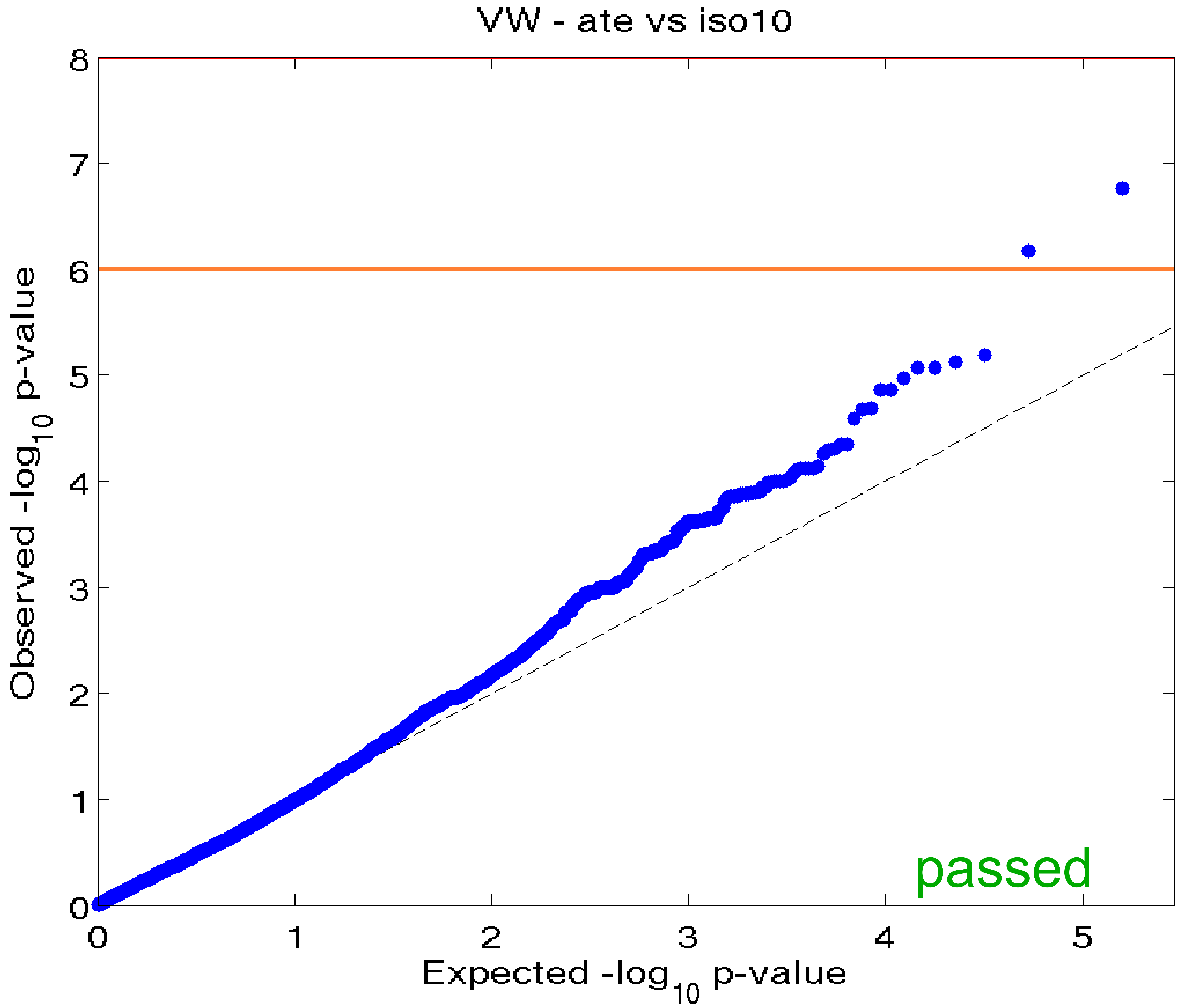
VW - ate vs iso10
Observed -log10 p-value
Expected -log10 p-value
0 1 2 3 4 5
0 1 2 3 4 5 6 7 8
passed
