# Supplementary figures and images for: Mapping Genetic Variants Associated with Beta-Adrenergic Responses in Inbred Mice
Source: PLoS One. 2012 Jul 31;7(7):e41032. doi: 10.1371/journal.pone.0041032 (PMC3409184; doi:10.1371/journal.pone.0041032)

**Expression value histogram**

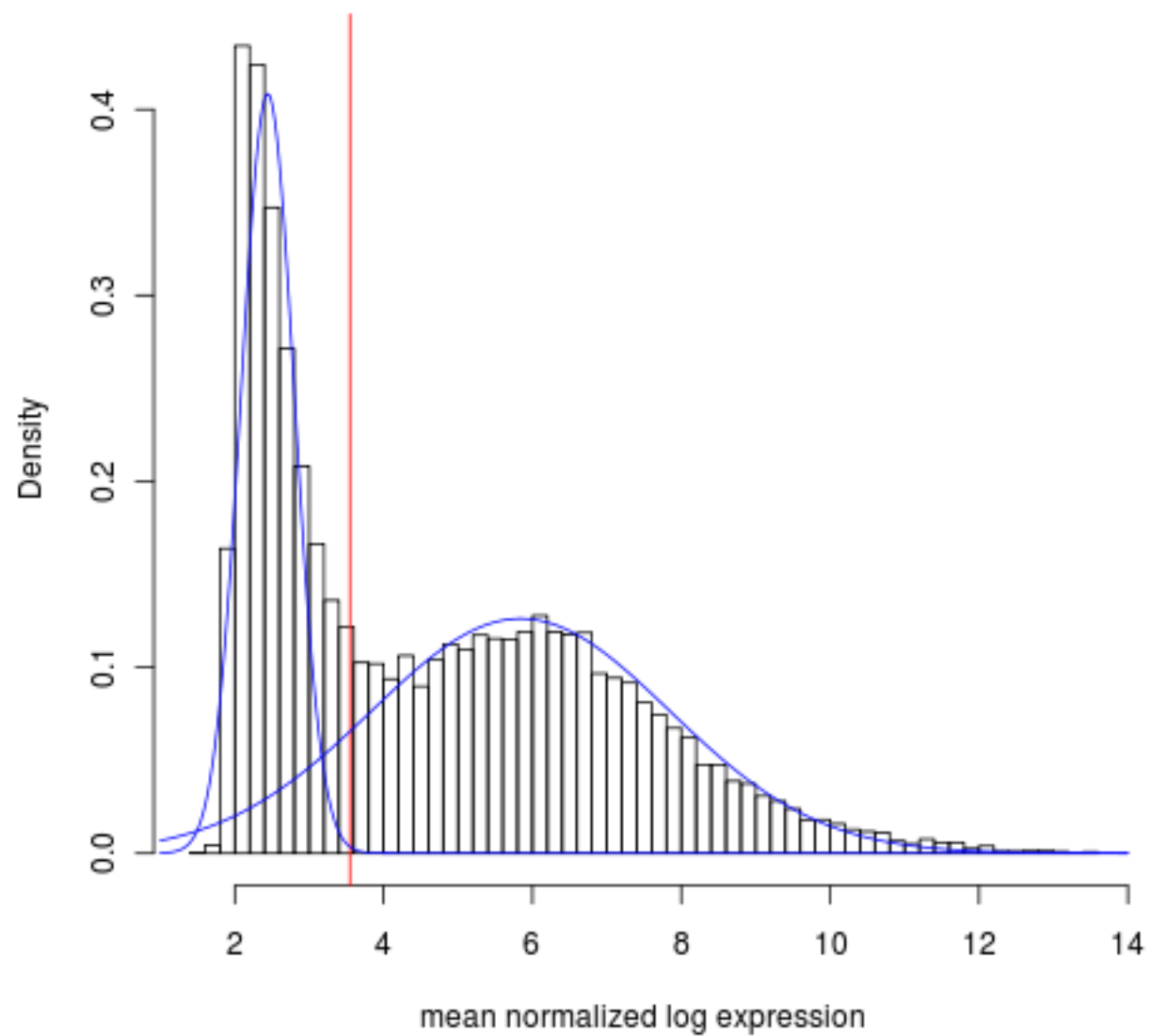

Supplement: Figure S9 — Distribution of cardiac mRNA expression levels as measured by microarrays. The mixture of two Gaussian functions was fitted to the log expression values. The first component, peaking at around 2, fits the non-expressed genes, whereas the second component, peaking at around 6.5, fits the expressed genes. Non-expressed genes are those which belong to the first component with at least 95% confidence, i.e., those genes that have a log expression value less than 2.33 (red line). (PDF) [file pone.0041032.s009.pdf]

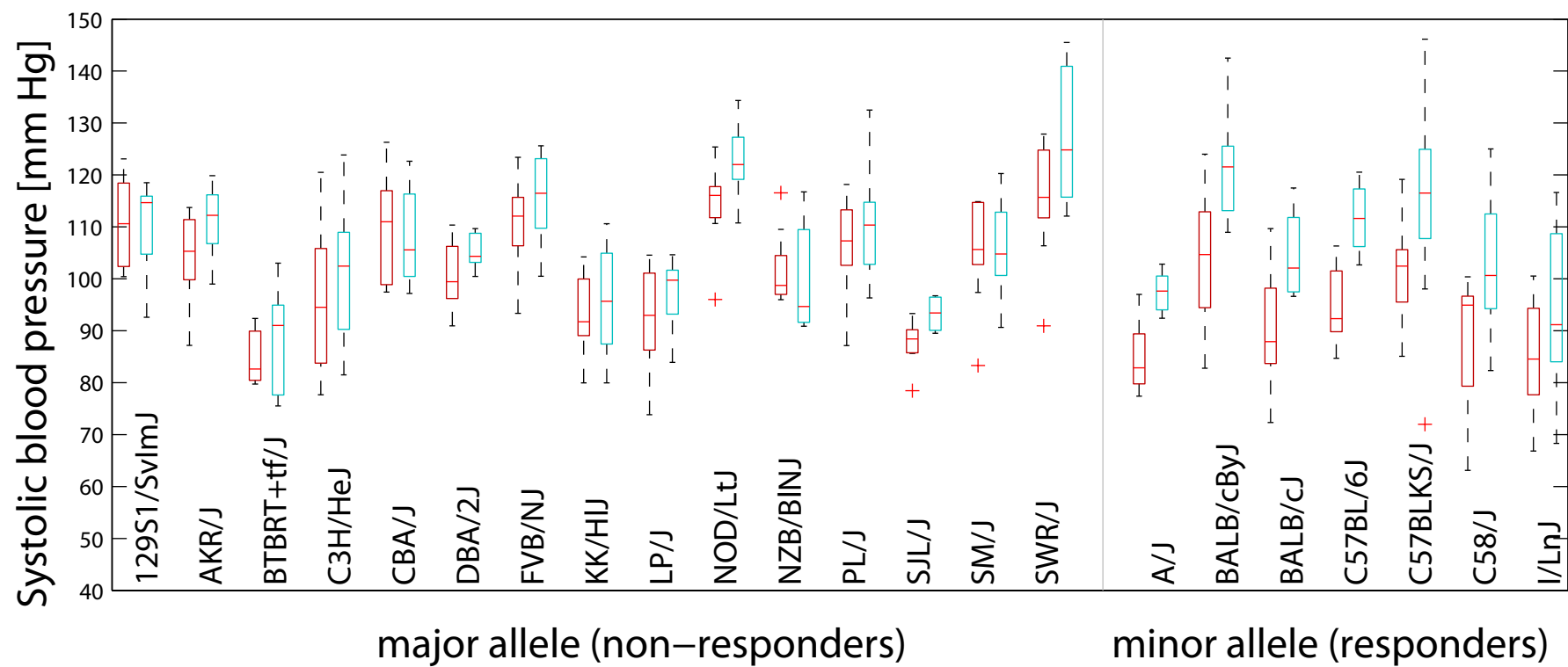

Supplement: Figure S10 — Boxplots showing the SBP values of 22 strains treated with ate (in red) and iso10 (in blue). Strains are segregated according to their genotype at marker rs29354390 in Trhrde (locus AE6). (PDF) [file pone.0041032.s010.pdf]

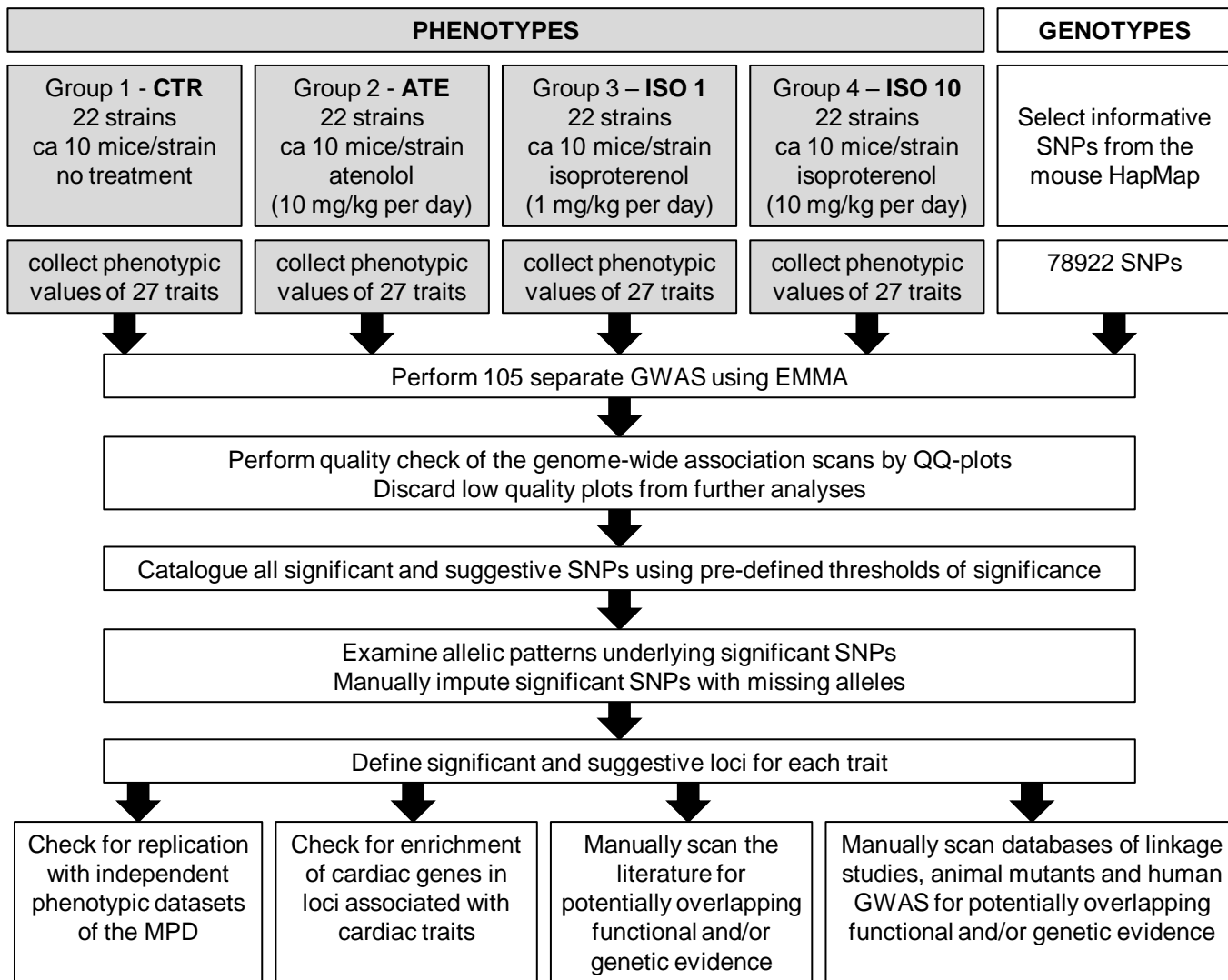

Supplement: Figure S11 — Analysis flowchart. This flowchart summarizes the analysis of the 27 phenotypes measured across the 22 inbred strains. Details referring to the boxes shaded in grey have been published previously [9]. These phenotypes are also freely available through the Mouse Phenome Database (project Maurer1; http://phenome.jax.org/). Analyses of the drug responses were performed following a similar scheme, except that the values were not physically measured in individual mice but approximated, as detailed in the main text. (PDF) [file pone.0041032.s011.pdf]
